# Multiscale Investigation of the Mechanism and Selectivity of CO_2_ Hydrogenation over Rh(111)

**DOI:** 10.1021/acscatal.3c05939

**Published:** 2024-03-28

**Authors:** Shijia Sun, Michael D. Higham, Xingfan Zhang, C. Richard A. Catlow

**Affiliations:** † Kathleen Lonsdale Materials Chemistry, Department of Chemistry, University College London, London WC1H 0AJ, United Kingdom; ‡ Research Complex at Harwell, Rutherford Appleton Laboratory, Harwell, Oxon OX11 0FA, United Kingdom; § School of Chemistry, Cardiff University, Park Place, Cardiff CF10 1AT, United Kingdom

**Keywords:** CO_2_ hydrogenation, density functional theory, kinetic Monte Carlo, reaction pathways, product selectivity, rhodium catalyst, temperature effect

## Abstract

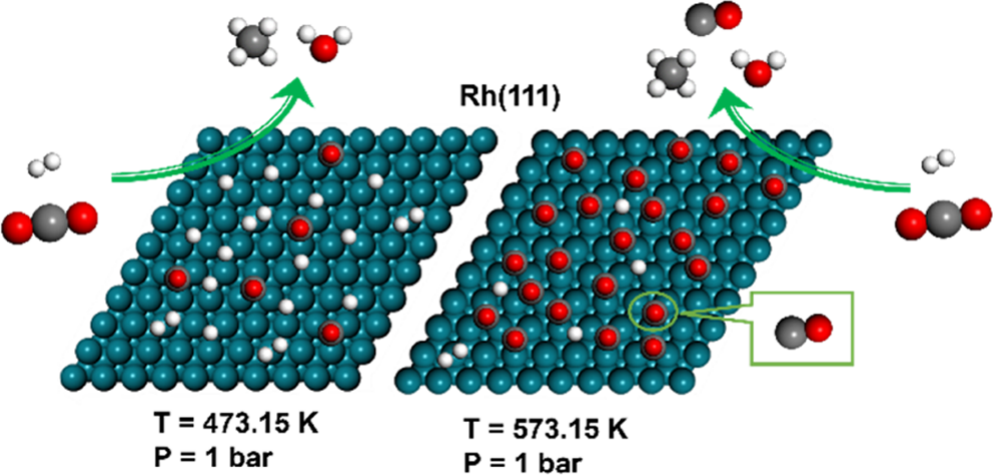

CO_2_ hydrogenation over Rh catalysts comprises multiple reaction pathways, presenting a wide range of possible intermediates and end products, with selectivity toward either CO or methane being of particular interest. We investigate in detail the reaction mechanism of CO_2_ hydrogenation to the single-carbon (C1) products on the Rh(111) facet by performing periodic density functional theory (DFT) calculations and kinetic Monte Carlo (kMC) simulations, which account for the adsorbate interactions through a cluster expansion approach. We observe that Rh readily facilitates the dissociation of hydrogen, thus contributing to the subsequent hydrogenation processes. The reverse water–gas shift (RWGS) reaction occurs via three different reaction pathways, with CO hydrogenation to the COH intermediate being a key step for CO_2_ methanation. The effects of temperature, pressure, and the composition ratio of the gas reactant feed are considered. Temperature plays a pivotal role in determining the surface coverage and adsorbate composition, with competitive adsorption between CO and H species influencing the product distribution. The observed adlayer configurations indicate that the adsorbed CO species are separated by adsorbed H atoms, with a high ratio of H to CO coverage on the Rh(111) surface being essential to promote CO_2_ methanation.

## Introduction

Energy-efficient catalytic CO_2_ conversion using renewable energy has attracted considerable attention as a potentially feasible means to mitigate CO_2_ emissions and produce commodity fuels and chemicals.^1−3^ It is thermodynamically feasible to hydrogenate CO_2_ to produce hydrocarbons (olefins, liquid hydrocarbons, and aromatics) and oxygenates (alcohols and dimethyl ether).^4,5^ One of the most important products is methane (CH_4_), which can be injected into existing natural gas infrastructure for distribution and storage, and for long-term chemical storage of electricity produced from renewable sources.^6,7^ In addition, methane can also be used as a feedstock material for the production of chemicals and fuels, including alkenes, gasoline, and aromatic compounds.^8−10^

Methane can be obtained via the well-known Sabatier reaction,^11^ a highly exothermic process that nonetheless requires very active catalysts to alleviate the high kinetic barriers arising from the eight-electron reduction of CO_2_ involved in the reaction process.^12,13^ Transition metals including Ni,^14−17^ Rh,^18−20^ Ru,^21−24^ and Pd^25−27^ have been used as catalysts for CO_2_ methanation, with Ni-based catalysts in particular exhibiting excellent CO_2_ hydrogenation activity at elevated temperatures, with high CH_4_ selectivity, although conversion rates are much lower at lower temperatures.^11^ In contrast, Rh-based catalysts show almost 100% CH_4_ selectivity and extremely high production rates even at lower temperatures.^28,29^ Supported Rh catalysts are normally used in experimental studies^30^ and the low-index Rh(111) facet is usually selected to explore the role of the Rh in catalytic reactions,^31−36^ since the Rh(111) surface is the most stable facet and therefore accounts for the largest surface area fraction in synthesized Rh particles.^37−39^ However, the mechanistic routes for CO_2_ hydrogenation are multiple and complex, with the precise nature of the intermediates remaining poorly understood.^5,12,40^

There are two categories of reaction mechanism proposed for CO_2_ methanation: dissociative, whereby C–O bond cleavage takes place before hydrogenation; and associative, in which hydrogenation takes place before C–O bond cleavage. In the former case, CO_2_ undergoes dissociative adsorption, resulting in the formation of CO* and O* species co-adsorbed on the surface, followed by CO* dissociation to O* and C* species, which subsequently undergo the hydrogenation to methane.^41,42^ In the latter case, CO_2_* and CO* can be directly hydrogenated to H_*X*_CO_Y_* and H_*X*_CO*_Y_*H* species, with subsequent C–O bond cleavage yielding CH_x_* intermediates for further hydrogenation to methane.^43−48^

Several experimental and computational studies have demonstrated the feasibility of dissociation of chemisorbed CO_2_ into CO species over Rh catalysts.^19,28,49−52^ Somorjai and co-workers found that CO_2_ appeared to dissociate to CO upon adsorption on Rh(111) and (100) surfaces, as indicated by the identical ordering and desorption characteristics of these two molecules.^51^ In addition, by combining scanning tunneling microscopy (STM), X-ray photoelectron spectroscopy (XPS) at near-ambient pressure (NAP), and computational techniques, Park and co-workers observed the cleavage of the O–CO bond on Rh(111) surfaces at room temperature.^53^ However, it has been found that the subsequent CO* dissociation on Rh catalysts is much less significant than for Ni and Ru catalysts.^54−57^ Yates et al. have shown that the Rh(111) facet is inactive for CO* dissociation below 870 K at low pressures, by means of isotopic exchange measurements.^58^ In addition, Solymosi et al. concluded that the adsorbed CO* could undergo dissociation to a limited extent on a supported Rh catalyst above 473 K at high pressures, which was attributed to the influence of the support.^54,59^ Under hydrogenating conditions, the adsorbed CO* can either desorb to the gas phase, with the remaining O* species being hydrogenated to water to complete the cycle for the reverse water–gas shift (RWGS) mechanism,^60^ or interact with co-adsorbed H* to form intermediate complexes.^61^ Jacquemin et al.^62^ concluded that adsorbed CO_2_ can undergo dissociation on a Rh/γ-Al_2_O_3_ catalyst, with subsequent reaction of CO* with H_2_, as revealed by *in situ* DRIFTS experiments. Karelovic and Ruiz studied the reaction mechanisms for CO_2_ hydrogenation over the supported Rh catalysts at low temperatures and proposed that CO is an important intermediate, with the CO* dissociation barrier being comparable to that of the overall reaction.^18,19^ Recently, several theoretical studies of the reaction mechanism for Rh-catalyzed CO_2_ methanation have been published; Kwon and co-workers^63^ applied DFT techniques to study the reaction pathways for CO_2_ hydrogenation on Rh(111), demonstrating that Rh can facilitate the direct dissociation of CO_2_ and that the lowest-energy reaction pathway for CO* hydrogenation to methane was via the formation and dissociation of HCO*, with HCOH* formation and dissociation as a plausible alternative. Similarly, DFT calculations were used to investigate the rate-determining step for CO_2_ methanation on the Rh(100) surface, which showed that hydrogen can assist the dissociation of CO*, via hydrogenation to CHO* and its subsequent dissociation to CH* and O*.^61^ In addition, *ab initio* molecular dynamics was applied to study CO activation on Rh surfaces and concluded that CO* more readily undergoes hydrogenation than dissociation.^64^ Furthermore, it was found that the strong Rh–CO interaction can impede CO hydrogenation, thus slowing down the overall process.^33^ The exact role of the intermediate species generated during the reaction process has, however, not been conclusively identified; they may be spectators (having only a minor influence on the mechanistic path), or key reaction intermediates (playing an important role in the reaction mechanism). As a result, further fundamental studies of the reaction mechanism for CO_2_ methanation over the Rh-based catalysts are necessary.

Density Functional Theory (DFT), combined with kinetic Monte Carlo (kMC) simulations, are powerful tools for exploring reaction mechanisms under realistic conditions, which can complement *operando* experimental techniques, and provide a full mechanistic description of CO_2_ conversion on the catalyst.^65−69^ In this study, we apply a multiscale approach to investigate the mechanistic pathways of CO_2_ hydrogenation on the Rh(111) surface, first by calculating activation and reaction energies for all elementary processes from DFT simulations. Secondly, we implement the DFT-calculated energies within the kMC method and are therefore able to identify the most feasible reaction pathways and product selectivity. The simulations incorporate interaction energies for the two-body terms used in the cluster expansion model, along with the rate constants for 52 reversible surface reactions, 8 reversible adsorption processes (involving H_2_ dissociative adsorption), as well as H atom diffusion process. We investigate the lattice configurations under realistic conditions, as well as the effect of temperature, pressure, and the composition ratio of the gaseous reactant mixtures on the distribution of products over the Rh catalysts.

## Computational Methods

### Plane-Wave DFT Calculations

Plane-wave DFT calculations were performed using the Vienna Ab initio Simulation Package (VASP) code^70,71^ in order to explore the reaction mechanisms for CO_2_ hydrogenation over the Rh(111) surface. Inner electrons were treated as projector-augmented waves (PAW),^72^ and the valence states were expanded in plane waves with a cutoff energy of 450 eV. Table S1 reports a comparison between the adsorption energies calculated with cutoff energy of 450 and 550 eV, with no significant difference being observed. Hence, a cutoff energy of 450 eV was deemed to be sufficient for the expansion of the valence states in plane waves. The Perdew–Burke–Ernzerhof (PBE) exchange–correlation functional was used throughout the study,^73^ and a dispersion correction was applied using the D3 scheme,^74^ in order to account for weak van der Waals interactions. The adsorption energies of the species accounting for vibrational zero-point energies have been calculated with PBE and PBE+D3 methods and compared (Table S1), which confirmed the importance of dispersion correction for molecular adsorption processes. The optimized bulk Rh lattice parameter was determined to be 3.83 Å, in good agreement with the experimental value of 3.79 Å.^75^

The slab model used for the Rh(111) facet consisted of six layers, separated by 18 Å of vacuum in the *z*-direction, to avoid spurious interactions between surfaces in adjacent periodic cells. For the purposes of modeling adsorption and reaction processes, a p(3 × 3) supercell was used for the Rh(111) surface. A Monkhorst–Pack k-point sampling scheme was determined commensurately with the slab supercell dimensions,^76^ with a *k*-grid of dimensions (3 × 3 × 1) applied. During structural optimization, the top four Rh layers were allowed to relax, while the bottom two were fixed at their optimized bulk lattice positions. A dipole correction was applied for all surface calculations to eliminate any spurious electrostatic interactions arising from the asymmetric relaxation of the surface slab. The six-layer slab model was determined to be suitable from test calculations exploring the relationship between the surface energy and the number of slab layers. Further details can be found in Section S2 in the Supporting Information.

Structural optimizations were regarded as being sufficiently well-converged when all ionic forces were minimized to within 0.01 eV Å^–1^. The SCF energy convergence threshold for electronic structure optimization was set to 10^–5^ eV. To explore the elementary processes involved in the reaction mechanism for CO_2_ hydrogenation, the activation energies for each process were calculated by performing Climbing Image Nudged Elastic Band (CI-NEB)^77,78^ and Improved Dimer Method (IDM)^79,80^ calculations, with atomic forces converged to within 0.03 eV Å^–1^. Vibrational analysis was used to confirm that the obtained transition state represented a true saddle point, indicated by the presence of a single imaginary vibrational frequency, corresponding to the unstable mode.

The activation energy (*E*_a_) and reaction energy (*E*_r_) for the elementary steps were obtained from the following:
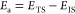
1
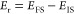
2where *E*_IS_, *E*_TS_, and *E*_FS_ are the energies of the optimized initial state, transition state, and final state for each step in the reaction mechanism.

The adsorption energy (*E*_ads_) was determined from the following:
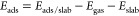
3where *E*_ads/slab_ and *E*_slab_ are the calculated energies of the optimized surfaces with and without adsorbate, respectively, and *E*_gas_ is the energy of the optimized gas-phase adsorbate.

The zero-point energy (ZPE) was calculated from the vibrational frequency according to the following:
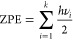
4where *h* is Planck’s constant and ν_*i*_ is the vibrational frequency.

### kMC Simulations

In our study, kMC simulations were performed using the Graph-Theoretical kinetic Monte Carlo (GT-kMC)^81,82^ approach, as implemented in the Zacros code,^83^ which has been successfully applied to investigate heterogeneous catalysis reaction on metal surfaces.^67−69,81^ For example, kMC simulations were applied to show the different reaction orders with respect to O coverage at high and low temperatures for CO oxidation on the Pd(111) surface.^67^ To account for the impact of lateral interactions, the cluster expansion model has been used to describe the contributions of single- and multibody adsorbates on the surface, allowing the determination of spatial correlations and coverage-dependent activation barriers. The impact of lateral interaction on activation barriers for elementary processes is parametrized in terms of a Brønsted–Evans–Polanyi (BEP) relationship, allowing activation barriers to be adjusted dynamically with surface coverage,^82^ which was applied in our simulations. For all elementary processes, a proximity factor of ω = 0.5 was applied. The impact of the choice of ω value is discussed in Section S4 in the Supporting Information, and simulations showed that reasonable variations in this parameter have no significant impact on the product distribution. Further information on the cluster expansion model can also be found in Sections S3 and S4 in the Supporting Information. Furthermore, since processes such as adsorption, desorption, and diffusion, typically have rate constants many orders of magnitude higher than those of rate-limiting surface reactions, they will typically be sampled much more frequently in a multiple time-scale-disparate simulation,^84^ thus hindering kMC time progression. Hence, Zacros version 2.0 implements a temporal acceleration scheme developed by Chatterjee and Voter,^85,86^ which automatically scales all fast quasi-equilibrated processes. This achieves dynamic detection of time-scale separation and dynamic scaling of the kinetic constants to accelerate the simulation. Hence, our simulations have the advantage of balancing the occurrence frequencies of both fast (like CO_2_ adsorption, H_2_ dissociative adsorption, and H atom diffusion) and slow elementary events (like CO_2_ or CO hydrogenation or dissociation), reducing the computational expense required to achieve useful kMC time progression. The rate constants for the elementary processes were estimated from the Arrhenius expression, including the DFT-calculated activation energies and the pre-exponential factors, resulting from the partition functions based on the DFT predicted vibrational frequencies. The corresponding mathematical expressions can be found in Section S4. All of the reaction steps are treated as reversible processes, including the adsorption and desorption processes. Further details of the general methodology applied can be found in previously published articles.^82,87^

**Figure 1:**
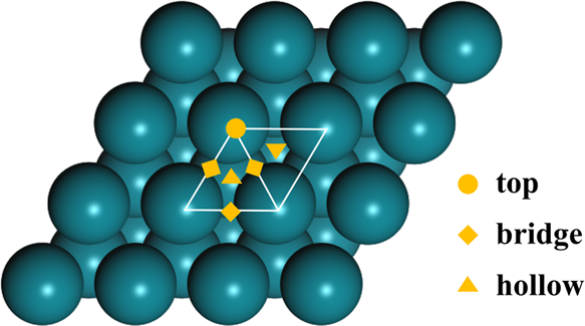
Top view of the Rh(111) surface (teal spheres represent Rh atoms). The defined adsorption sites used in the kMC simulation are indicated by yellow icons. The white parallelogram represents the surface unit cell.

The reaction network comprises 52 reversible surface reactions, 8 reversible adsorption processes (including H_2_ dissociative adsorption), as well as an H atom diffusion process over the Rh(111) surface. The lattice model consists of three kinds of active sites, including one top site, three bridge sites, and two hollow sites (with fcc and hcp hollow sites being essentially equivalent) within the Rh(111) surface unit cell, as shown in Figure 1, comprising a total of 15 000 sites on the 50 × 50 kMC lattice. The model comprises 8 gaseous species and 25 surface species, which can occupy one, two, or three neighboring adsorption sites as determined by DFT calculations. The cluster expansion model considers the lateral interactions for the most relevant co-adsorbed species at the nearest neighboring adsorption sites, comprising a total of 57 pairwise interactions, as shown in Table S3–1; the evaluation of additional lateral interactions between CO and the remaining species are provided in Section S3 in the Supporting Information, and validate the cluster expansion model applied. The input gas consisted of a mixture of H_2_ and CO_2_ in a ratio of 4:1 over the clean Rh(111) surface at the temperatures of 473.15 and 573.15 K under a pressure of 1 bar, allowing us to consider the effect of temperature on the activity and selectivity. In addition, several kMC simulations were performed using different seeds to minimize errors.

## Results and Discussion

### DFT Calculations

#### CO_2_ Adsorption

The adsorption energies and geometric parameters for different CO_2_ adsorption modes over Rh(111) are shown in Table 1. Two distinct CO_2_ adsorption geometries were identified: a linear, physisorbed, CO_2_ species, and a bent, chemisorbed, CO_2_ species. CO_2_ adsorption is slightly exothermic, with ZPE-corrected adsorption energies of −0.23 and −0.33 eV for the physisorbed and chemisorbed CO_2_, respectively. This value is comparable to the calculated CO_2_ chemisorption energy (−0.39 eV) over the Rh(111) surface as reported by Kim et al.^53^ The optimized structure for physisorbed CO_2_ shows no significant changes compared to the geometry of gaseous CO_2_. However, for the chemisorbed CO_2_ species, a significant distortion of CO_2_ appears, with the O–C–O angle shrinking to 134.5°, and the C–O distance modestly lengthening, implying the weakening of the C=O bonds, corresponding to the activation of CO_2_ over Rh(111). CO_2_ chemisorption on the Rh(111) surface thus presents a bent geometry, with one O atom binding to a metal atom, and the C atom binding to the nearest metal atom, which is similar to the results obtained previously.^88−93^

**Table 1:** ZPE-Corrected Adsorption Energy (*E*_ads_) for Physisorbed and Chemisorbed CO_2_ over the Rh(111) Surface, with C–Rh Distance (*d*_(C–Rh)_), C–O Distance (*d*_(C–O)_), and O–C–O Angle (∠_(O–C–O)_), as well as the Bader Charge Difference (β) for the Physisorbed and Chemisorbed CO_2_

species	*E*_ads_ (eV)	*d*_(C–Rh)_ (Å)	*d*_(C–O)_ (Å)	∠_(O–C–O)_ (deg)	β (|e|)
Phys-CO_2_	–0.23	3.38	1.18	179.6	–0.05
Chem-CO_2_	–0.33	2.06	1.22, 1.28	134.5	–0.46

To investigate CO_2_ activation further, a Bader charge analysis was performed, showing that there is *a* charge transfer of 0.46 e^–^ from the Rh surface to chemisorbed CO_2_, while the value is only 0.05 e^–^ for physisorbed CO_2_. In other metallic catalysts, Higham et al. observed an increase in charge transfer of 0.70 e^–^ from the physisorbed to the chemisorbed CO_2_ on both the Cu(100) and (110) surfaces,^93^ although in this study the activated CO_2_ species was metastable, in contrast to the behavior reported in the present work for Rh. In addition, Mulliken charge analysis of the physisorbed and chemisorbed CO_2_ on the Pd(111), (110), and (100) surfaces was studied by Kowalec et al., which indicates only a limited extent of CO_2_ reduction for physisorbed CO_2_ (0.04, 0.10, and 0.11 e^–^ on the Pd (111), (100), and (110) surfaces), and a much greater extent of CO_2_ reduction for the chemisorbed CO_2_ on the three Pd surfaces.^92^ In contrast, the increase of the charge transfer from the physisorbed to the chemisorbed CO_2_ on metal carbide catalysts is much greater, as Quesne et al. observed that the valence electron count for chemisorbed CO_2_ increased by one electron on the low-index surfaces of TiC, VC, ZrC and NbC catalysts,^94^ implying a much greater extent of CO_2_ reduction for these catalysts. Hence, the Bader charge analysis for CO_2_ adsorption on Rh(111) can be interpreted as indicating that the chemisorbed CO_2_ is partially reduced, with the charge accumulation being mainly localized on the C atom. The partial CO_2_ reduction process can be interpreted in terms of charge transfer from the Rh surface to the C=O π* antibonding orbitals of CO_2_ species (thus accounting for the modest increase in C–O bond length), which facilitates CO_2_ activation, and therefore the subsequent reaction of the activated CO_2_ (i.e., dissociation or hydrogenation). Similarly, by employing the experimental methods of NAP-STM and NAP-XPS, Kim et al.^53^ have directly observed that the linear geometry of CO_2_ gas molecules evolves into a chemically active bent structure over the Rh(111) surface, with changes of local charge density at the CO_2_–Rh(111) interface for the cleavage of C=O bond, thus corroborating our computational results.

### H_2_ Dissociative Adsorption

In addition to CO_2_ adsorption and activation, H_2_ dissociative adsorption is a key prerequisite for any CO_2_ hydrogenation catalyst. Hence, it is necessary to establish the adsorption and dissociation behavior of H_2_ over the Rh catalyst surface. Previous studies have found that for H_2_ molecular adsorption, binding at the top site, oriented parallel to the metal surface is most stable, and that subsequent dissociation of H_2_ is more thermodynamically favorable than desorption on the Rh(100) surface.^95^ Additionally, *in situ* DRIFTS studies performed at 300 °C have shown that the dissociative adsorption of H_2_ readily proceeds on Rh catalysts.^96^

**Figure 2:**
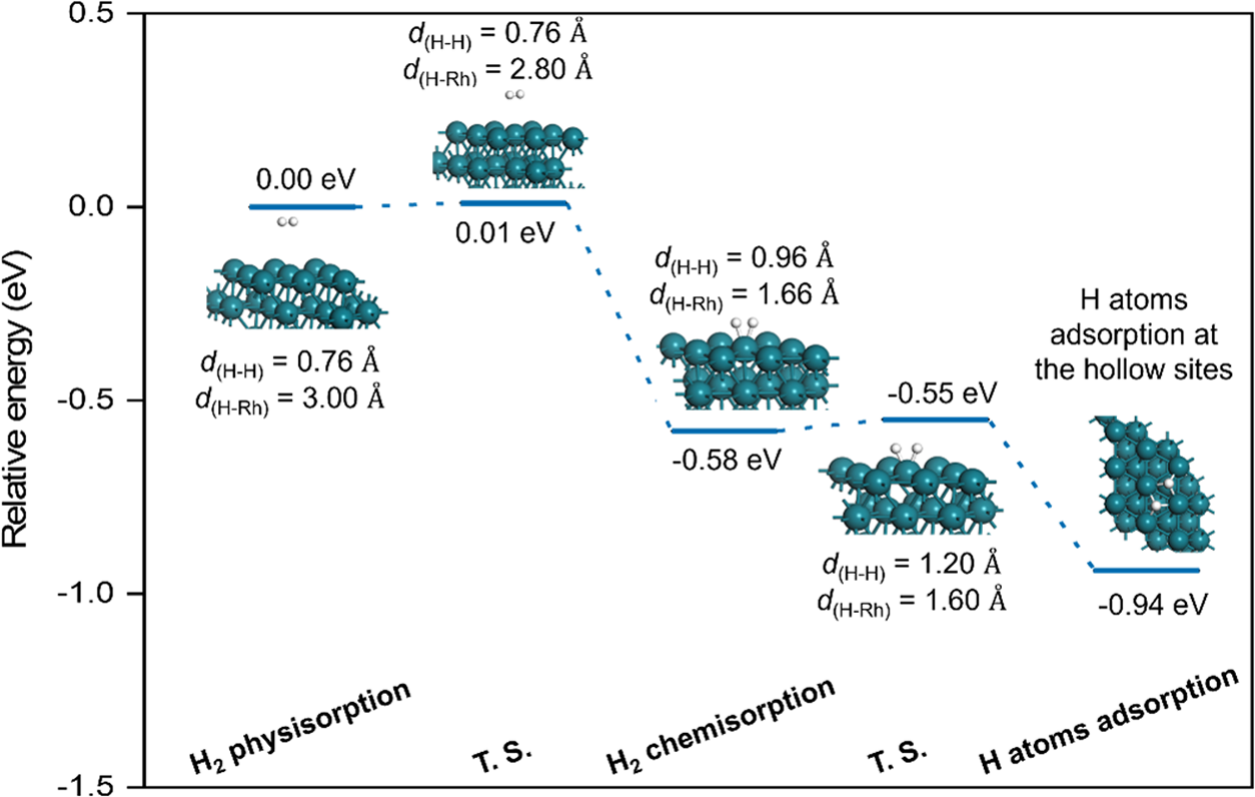
ZPE-corrected relative energies and the structures for the processes of H_2_ adsorption and dissociation. The transition states (T.S.) for H_2_ dissociative adsorption and H diffusion processes are also shown here. *d*_(H–H)_ and *d*_(H–Rh)_ represent the H–H distance and H–Rh distance, respectively. The teal and white spheres are the Rh and H atoms, respectively. The zero-energy state corresponds to physisorbed H_2_.

We found the ZPE-corrected physisorption energy of H_2_ to be −0.09 eV, with the molecule located 3.00 Å away from the Rh surface. However, the H–H bond is elongated from 0.76 to 0.96 Å when H_2_ chemisorbs at the top site of the Rh(111) surface, and the H–Rh distance is 1.66 Å, which is in agreement with the previously reported results.^97^ The calculated structures, ZPE-corrected relative energies, and geometric parameters for H_2_ adsorption and dissociation are reported in Figure 2. As noted, the adsorption of H_2_ at the top site results in considerable H–H bond elongation (*d*_(H–H)_ = 0.96 Å), suggesting that the Rh catalyst readily promotes H_2_ activation, with full dissociation; the transition from the physisorbed H_2_ to the chemisorbed H_2_ at the top site has a negligible activation energy of 0.01 eV. To better understand the electronic structures for H_2_ dissociative adsorption at the top site, Density of States (DOS) and Crystal Orbital Hamilton Population (COHP) methods were applied to analyze the change of the H–H bond from physisorption to chemisorption, as described in Sections S5 and S6 in the Supporting Information. In addition, H atoms were found to adsorb more exothermically at the hollow sites on the Rh(111) surface, and the activation energy for H diffusion from the top site to the hollow site is only 0.03 eV, suggesting that the elongated H_2_ molecule at the top site readily dissociates to yield two H* species on the adjacent hollow sites. These results, therefore, clearly show that the Rh(111) surface promotes H_2_ dissociative adsorption, thus facilitating the subsequent hydrogenation processes.

### Reaction Network for CO_2_ Hydrogenation

**Figure 3:**
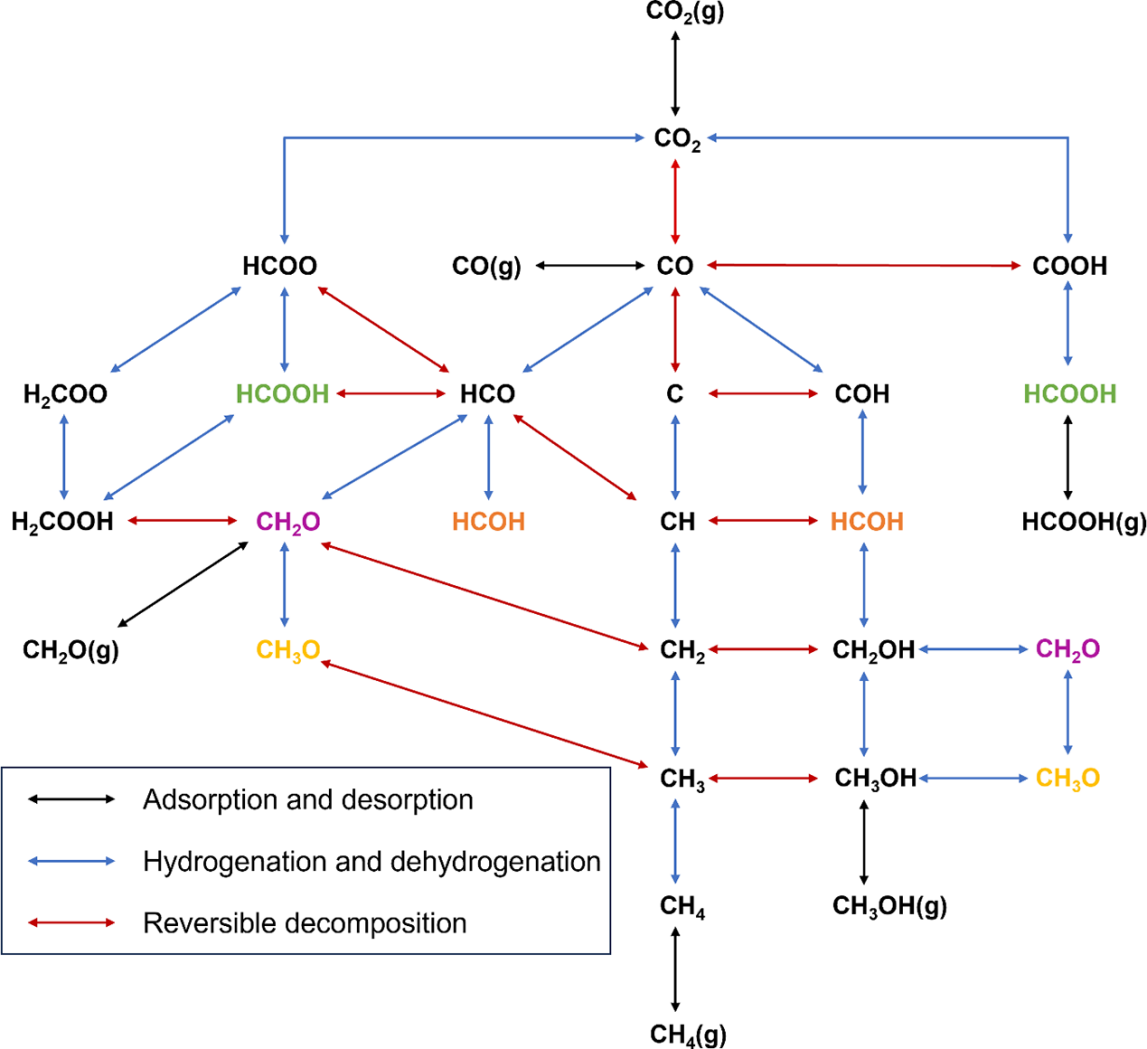
Reaction network for CO_2_ hydrogenation into C1 species, with possible products comprising gaseous CO, CH_4_, CH_3_OH, HCOOH, and CH_2_O. The black, blue, and red lines signify the adsorption and desorption processes, hydrogenation and dehydrogenation processes, and reversible decomposition processes, respectively. Species appearing in more than one mechanistic pathway are identified with colored labels for clarity (e.g., adsorbed HCOOH is denoted by text in green). All species are adsorbed on the Rh(111) surface, except for those marked with (g), which indicates gaseous entities.

For CO_2_ hydrogenation, there are multiple, often overlapping, reaction pathways leading to product formation. Hence, all possible elementary processes are considered to obtain a complete reaction network (Figure 3). The favored product results from the most kinetically feasible (i.e., least energy-demanding) pathway proceeding via the most stable intermediates. To provide further insight into the reaction mechanisms, the ZPE-corrected activation energies (*E*_a_) and reaction energies (*E*_r_) for all of the possible relevant reaction pathways are summarized in Table 2.

**Table 2:** Calculated ZPE-Corrected Activation Energies (*E*_a_) and Reaction Energies (*E*_r_) for the Elementary Steps

	elementary steps	ZPE-corrected *E*_a_ (eV)	ZPE-corrected *E*_r_ (eV)
R1	CO_2_ + * ↔ CO + O	0.45	–0.98
R2	CO + * ↔ C + O	2.74	0.93
R3	CO + H ↔ COH	1.27	0.71
R4	CO + H ↔ HCO	1.35	1.21
R5	COH + H ↔ HCOH	1.31	1.07
R6	COH ↔ C + OH	1.68	0.55
R7	HCO + H ↔ CH_2_O	0.70	0.51
R8	HCO + H ↔ HCOH	0.69	0.56
R9	HCO + * ↔ CH + O	1.17	–0.49
R10	CH_2_O + H ↔ CH_2_OH	0.75	0.27
R11	CH_2_O + H ↔ CH_3_O	0.74	0.37
R12	CH_2_O + * ↔ CH_2_ + O	0.95	–0.64
R13	HCOH + * ↔ CH + OH	0.47	–0.72
R14	HCOH + H ↔ CH_2_OH	0.59	0.21
R15	CH_2_OH ↔ CH_2_ + OH	0.75	–0.58
R16	CH_3_O + * ↔ CH_3_ + O	1.12	–0.64
R17	C + H ↔ CH	0.67	–0.20
R18	CH + H ↔ CH_2_	0.64	0.36
R19	CH_2_ + H ↔ CH_3_	0.63	0.38
R20	CH_3_ + H ↔ CH_4_	0.45	0.23
R21	CO_2_ + H ↔ COOH	0.72	–0.03
R22	COOH + * ↔ CO + OH	0.36	–0.62
R23	COOH + H ↔ HCOOH	1.34	0.51
R24	HCOOH + * ↔ HCO + OH	0.42	0.09
R25	HCOOH + H ↔ H_2_COOH	0.36	0.13
R26	H_2_COOH + * ↔ CH_2_O + OH	0.44	0.00
R27	CO_2_ + H ↔ HCOO	0.69	–0.13
R28	HCOO + * ↔ HCO + O	1.27	0.37
R29	HCOO + H ↔ HCOOH	0.87	0.61
R30	HCOO + H ↔ H_2_COO	2.24	1.56
R31	H_2_COO + H ↔ H_2_COOH	0.53	–0.35
*R*32	CH_2_OH + H ↔ CH_3_OH	0.73	0.15
R33	CH_3_O + H ↔ CH_3_OH	0.78	0.05
R34	CH_3_OH ↔ CH_3_+OH	1.67	–0.35
R35	O + H ↔ OH	0.80	0.33
R36	OH + H ↔ H_2_O	0.78	0.01
R37	OH + OH ↔ H_2_O + O	0.33	–0.32
R38	CO_2_ + OH ↔ COOH + O	0.10	–0.36
R39	CO_2_ + H_2_O ↔ HCOO + OH	1.49	–0.14
R40	CO_2_ + H_2_O ↔ COOH + OH	0.43	–0.04
R41	CO_2_ + OH ↔ HCOO + O	1.51	–0.46
R42	COOH + H ↔ COHOH	0.70	0.51
R43	COHOH ↔ COH + OH	0.73	–0.43
R44	CO + H_2_O ↔ COH + OH	1.01	0.69
R45	CO + CO ↔ CO_2_ + C	2.92	1.90
R46	CO + COOH ↔ CO_2_ + COH	0.74	0.73
R47	CO + HCOO ↔ CO_2_ + COH	0.94	0.83
R48	CO + OH ↔ O + HCO	1.87	0.88
R49	CO + H_2_O ↔ OH + HCO	1.63	1.20
R50	HCOO + HCO ↔ HCOOH + CO	0.35	–0.61
R51	HCOO + HCO ↔ H_2_COO + CO	0.95	0.35
R52	HCOOH + HCO ↔ H_2_COOH + CO	0.42	–0.62
A1	CH_4_ ↔ CH_4_(g) + *	0.20	0.20
A2	CH_3_OH ↔ CH_3_OH(g) + *	0.67	0.67
A3	CO ↔ CO(g) + *	2.28	2.28
A4	CH_2_O ↔ CH_2_O(g) + *	1.19	1.19
A5	HCOOH ↔ HCOOH(g) + *	0.79	0.79
A6	H_2_O ↔ H_2_O(g) + *	0.53	0.53
A7	CO_2_(g) + * ↔ CO_2_	0.00	–0.33
A8	H_2_(g) + * ↔ H + H	0.01	–0.58
D1	H(top)+ * ↔ H(hollow) + *	0.03	–0.36

### Reverse Water–Gas Shift (RWGS)

**Figure 4:**
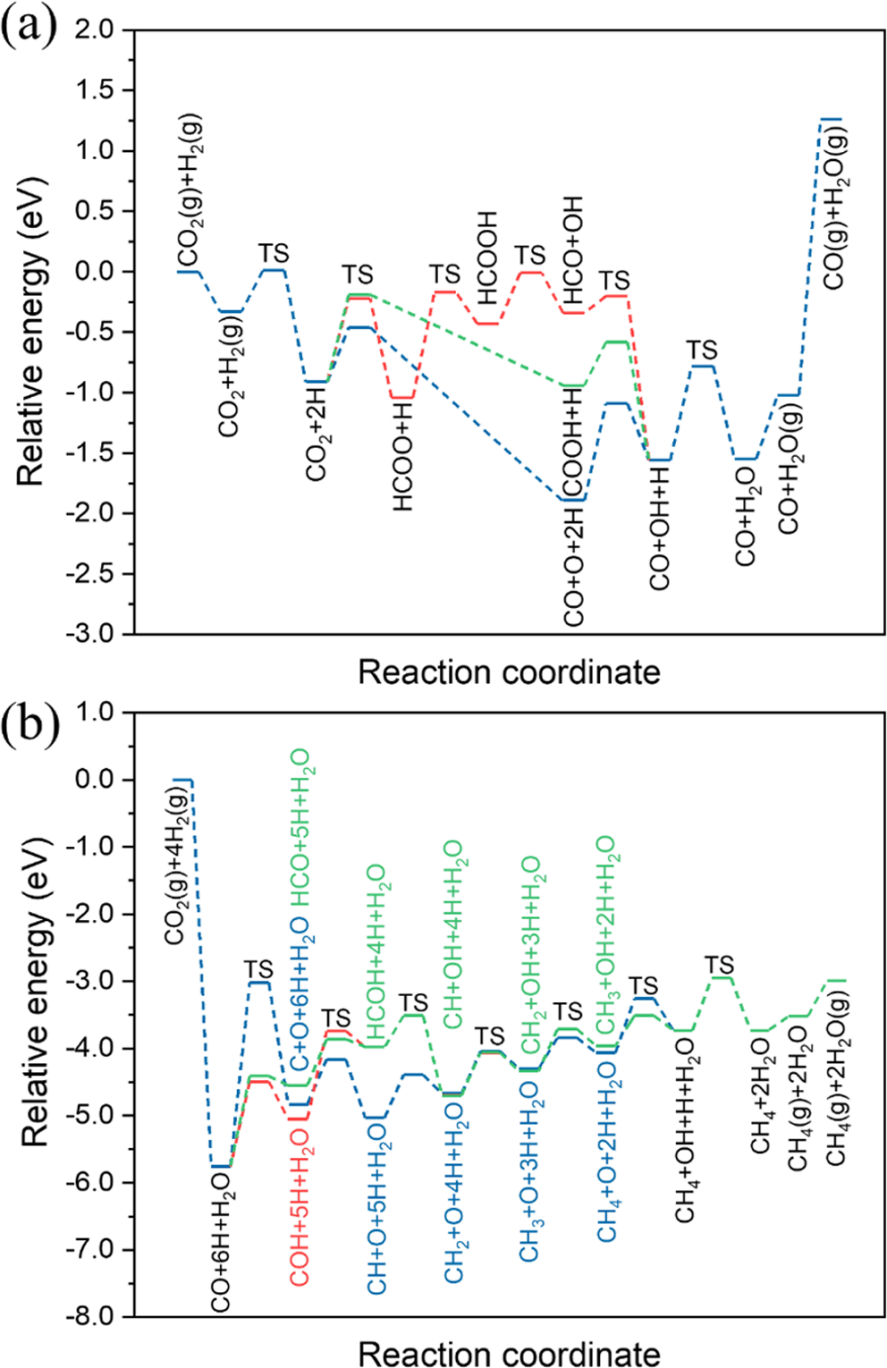
(a) Energy profiles for the RWGS reaction, including redox mechanism (blue line), formate mechanism (red line), and carboxyl mechanism (green line). (b) Energy profiles for CO_2_ methanation, including the pathways through the carbon (blue line), COH (red line), and HCO (green line). Transition states (TS) are labeled accordingly.

As shown in Figure 4, three different pathways for the RWGS reaction are considered: the redox, formate, and carboxyl mechanisms. We will address each of these mechanisms in turn, starting from adsorbed CO_2_.

For the redox mechanism, CO_2_ undergoes direct dissociation to yield co-adsorbed CO and O, with this process being exothermic and having only a moderate activation barrier (*E*_a_ = 0.45 eV and *E*_r_ = −0.98 eV). The surface O atom resulting from CO_2_ dissociation can react with the co-adsorbed H to produce OH, with this process having an activation energy of 0.80 eV and endothermic reaction energy of 0.33 eV. Subsequently, water can be produced by two pathways in all three mechanisms. The first is the direct hydrogenation of OH (*E*_a_ = 0.78 eV and *E*_r_ = +0.01 eV) to form H_2_O. The second is the reaction of two co-adsorbed OH species (*E*_a_ = 0.33 eV and *E*_r_ = −0.32 eV) to form H_2_O and adsorbed O, affording a lower activation barrier; hence, it is possible that OH disproportionation is the predominant mechanism by which water is formed.

For the formate mechanism, CO_2_ must first undergo hydrogenation, which can proceed via direct CO_2_ hydrogenation by co-adsorbed H, with this process being slightly exothermic and having a moderate activation barrier (*E*_a_ = 0.69 eV and *E*_r_ = −0.13 eV), although the activation barrier is greater than for direct CO_2_ dissociation as discussed in the previous paragraph. Alternatively, CO_2_ hydrogenation could proceed via hydrogenation by OH or H_2_O; however, the calculations reveal that both of these processes have much higher activation barriers (*E*_a_ = 1.51 eV and *E*_r_ = −0.46 eV for R41; *E*_a_ = 1.49 eV and *E*_r_ = −0.14 eV for R39 in Table 2). Hence, it is likely that most formate on the Rh(111) surface originates from direct CO_2_ hydrogenation. HCOO can then subsequently undergo further hydrogenation or dissociation; the calculations suggest that HCOO hydrogenation to HCOOH (*E*_a_ = 0.87 eV and *E*_r_ = +0.61 eV) is more kinetically accessible compared to both direct dissociation of HCOO into HCO and O (*E*_a_ = 1.27 eV and *E*_r_ = +0.37 eV), and H_2_COO formation via HCOO hydrogenation (*E*_a_ = 2.24 eV and *E*_r_ = +1.56 eV); although subsequent H_2_COO hydrogenation to H_2_COOH (*E*_a_ = 0.53 eV and *E*_r_ = −0.35 eV) has a lower activation energy, the H_2_COO species is likely to be kinetically inaccessible due to the high activation barrier for its formation. The mechanism can then proceed via the dissociation of HCOOH to HCO and OH (*E*_a_ = 0.42 eV and *E*_r_ = +0.09 eV), with the subsequent dissociation of HCO to produce CO having an even lower barrier and being highly exothermic (*E*_a_ = 0.14 eV and *E*_r_ = −1.21 eV). Notably, both of these processes have lower activation barriers than the HCOOH desorption energy (0.79 eV), suggesting that formic acid is likely to undergo further reactive processes, rather than being desorbed to the gas phase. Formic acid may also undergo hydrogenation to yield the H_2_COOH intermediate, although subsequent processes will probably result in the formation of formaldehyde, and subsequently methoxy, as will be discussed later. Hence, formic acid is likely to be a key intermediate in forming CO by the formate mechanism.

Finally, the carboxyl mechanism involves first the formation of COOH from hydrogenation of CO_2_. Direct hydrogenation by co-adsorbed H, like the corresponding process for formate formation, is slightly exothermic and has a moderate activation barrier (*E*_a_ = 0.72 eV and *E*_r_ = −0.03 eV). However, hydrogenation of CO_2_ to COOH via OH (*E*_a_ = 0.10 eV and *E*_r_ = −0.36 eV) or H_2_O (*E*_a_ = 0.43 eV and *E*_r_ = −0.04 eV) affords considerably lower activation barriers, potentially making the carboxyl RWGS pathway competitive with the redox mechanism already discussed. The mechanism proceeds via the dissociation of COOH to yield CO and OH, which has a modest activation barrier and is appreciably exothermic (*E*_a_ = 0.36 eV and *E*_r_ = −0.62 eV). By contrast, COOH hydrogenation to HCOOH is highly activated and endothermic (*E*_a_ = 1.34 eV and *E*_r_ = +0.51 eV), thus rendering the HCOOH dissociation mechanism to yield CO described above inaccessible from COOH.

In summary, the calculations suggest that the redox mechanism is the most likely RWGS pathway, in agreement with experimental results,^51,53^ although the calculations also suggest that CO_2_ hydrogenation to COOH via OH or H_2_O may render the carboxyl pathway a viable alternative. For all of the possible RWGS mechanisms described, it should be noted that the resulting CO is strongly bound to the Rh(111) surface, with an adsorption energy of −2.28 eV. The DFT-calculated adsorption energy at a temperature of 0 K and a coverage of 1/9 ML is more exothermic than the experimental value of −1.47 eV, which was determined at a temperature of 500 K and a coverage of 1/4 ML.^98^ This reflects the universal impact that surface coverage and temperature have on adsorption energies. The impact of surface coverage on the adsorption of CO on the Rh surface was corroborated by experimental investigations of the heat of CO adsorption on a reduced 3% Rh/Al_2_O_3_ varying with coverage, which varied from 195 kJ/mol at low coverage (θ = 0) to 103 kJ/mol at high coverage (θ = 1) for the linear CO species.^99^ Furthermore, the general tendency of the PBE functional to overestimate heats of adsorption on metal surfaces is well known,^100−102^ with adsorbates typically overbound by about 0.6 eV per adsorbate.^103^ Hence, the calculated adsorption energy is qualitatively consistent with the strong binding of CO on Rh(111), and quantitatively consistent with similar theoretical studies, as well as being commensurate with the experimentally determined values within the confines and limitations of the models and methods applied.^51,104−106^ The impact of CO binding energy on the reaction mechanisms and kMC product distribution will be discussed in more detail below and in Section S7 in the Supporting Information. Hence, the highly exothermic adsorption of CO on Rh(111) will therefore largely preclude its evolution to the gas phase, with CO undergoing either further dissociation or hydrogenation, to yield methanol or methane, which will be discussed in the following section.

### Pathways for Methane Formation

For methane formation, we propose three mechanistic pathways starting from the intermediate CO produced from the RWGS reaction. They proceed through carbon, COH, and HCO, respectively (Figure 4b). The carbon hydrogenation pathway involves surface C species formed via CO dissociation (*E*_a_ = 2.74 eV and *E*_r_ = +0.93 eV) and COH dissociation (*E*_a_ = 1.68 eV and *E*_r_ = +0.55 eV); both of these processes have very high activation barriers, suggesting that surface atomic C species are unlikely to be formed or play a significant role in the overall methanation mechanism. Similarly, the process involving CH formation via HCO dissociation (*E*_a_ = 1.17 eV and *E*_r_ = −0.49 eV) is also highly activated. The subsequent hydrogenation processes for surface C species, however, all have moderate activation barriers and are either modestly exo- or endothermic, proceeding via hydrogenation to CH (*E*_a_ = 0.67 eV and *E*_r_ = −0.20 eV), CH_2_ (*E*_a_ = 0.64 eV and *E*_r_ = +0.36 eV), CH_3_ (*E*_a_ = 0.63 eV and *E*_r_ = +0.38 eV), and finally CH_4_ (*E*_a_ = 0.45 eV and *E*_r_ = +0.23 eV). We note that while the calculations suggest that the surface C and CH species are unlikely to be formed via CO or HCO dissociation, the CH, CH_2_, and CH_3_ species may well be formed as a result of other processes, and their subsequent conversion to methane is likely to be accessible under typical conditions, as shown by the series of processes detailed above.

For the COH hydrogenation pathway, COH resulting from CO hydrogenation can undergo subsequent hydrogenation processes before C–O bond cleavage taking place to enable methane formation. While the direct hydrogenation of CO to COH is highly activated and moderately endothermic (*E*_a_ = 1.27 eV and *E*_r_ = +0.71 eV), it is more feasible that COH species are formed via the COOH hydrogenation to COHOH (*E*_a_ = 0.70 eV and *E*_r_ = +0.51 eV), which subsequently dissociates to COH (*E*_a_ = 0.73 eV and *E*_r_ = −0.43 eV). Other alternative pathways to COH formation, such as the interactions of CO with H_2_O (*E*_a_ = 1.01 eV and *E*_r_ = +0.69 eV), COOH (*E*_a_ = 0.74 eV and *E*_r_ = +0.73 eV), or HCOO (*E*_a_ = 0.94 eV and *E*_r_ = +0.83 eV), have higher activation barriers. The subsequent hydrogenation of COH to HCOH, however, has a higher activation barrier and is highly endothermic (*E*_a_ = 1.31 eV and *E*_r_ = +1.07 eV). The resulting HCOH intermediate could then either dissociate to CH (*E*_a_ = 0.47 eV and *E*_r_ = −0.72 eV) or undergo further hydrogenation to CH_2_OH (*E*_a_ = 0.59 eV and *E*_r_ = +0.21 eV), with both of these processes having much lower activation barriers. In the event of HCOH dissociation, CH can be sequentially hydrogenated to the final product CH_4_, as has already been discussed within the context of the surface carbon mechanism for methane formation. If CH_2_OH is formed, the intermediate can then undergo dissociation to CH_2_ (*E*_a_ = 0.75 eV and *E*_r_ = −0.58 eV), again followed by the further hydrogenation to the ultimate product CH_4_; hydrogenation of CH_2_OH to methanol will be discussed separately later in this work.

For the HCO pathway, HCO formation, as discussed previously, is likely to occur more readily via HCOOH decomposition (*E*_a_ = 0.42 eV and *E*_r_ = +0.09 eV), compared to the much less accessible direct CO hydrogenation (*E*_a_ = 1.35 eV and *E*_r_ = +1.21 eV). We also consider the interactions of CO with OH or H_2_O to yield HCO (CO+OH, *E*_a_ = 1.87 eV and *E*_r_ = +0.88 eV; CO+H_2_O, *E*_a_ = 1.63 eV and *E*_r_ = +1.20 eV), which are highly activated and endothermic. HCO can then undergo hydrogenation to formaldehyde (CH_2_O, *E*_a_ = 0.70 eV and *E*_r_ = +0.51 eV), which can then undergo hydrogenation to methoxy (CH_3_O, *E*_a_ = 0.74 eV and *E*_r_ = +0.37 eV), and CH_2_OH (*E*_a_ = 0.75 eV and *E*_r_ = +0.27 eV). Methoxy can then undergo dissociation to yield CH_3_ (*E*_a_ = 1.12 eV and *E*_r_ = −0.64 eV), and finally hydrogenation to CH_4_. For CH_2_OH species, the subsequent processes have been discussed for the COH hydrogenation mechanism. If CH_2_O undergoes dissociation (*E*_a_ = 0.95 eV and *E*_r_ = −0.64 eV), the resulting CH_2_ can then undergo further hydrogenation to the ultimate product CH_4_, in a manner analogous to that already discussed for the carbon pathway. While the calculated activation barriers for HCO hydrogenation and CH_3_O dissociation are higher than those for surface C hydrogenation, the relative ease of formation of the HCO intermediates means that its subsequent hydrogenation is likely to be of greater importance for the overall methanation mechanism. For example, HCOH resulting from HCO hydrogenation (*E*_a_ = 0.69 eV and *E*_r_ = +0.56 eV), is moderately activated and endothermic; and subsequent progress from HCOH to methane formation has been discussed within the context of the COH hydrogenation mechanism. In addition, HCO can react with other important co-adsorbed intermediates. HCO interacts with HCOO to yield HCOOH (*E*_a_ = 0.35 eV and *E*_r_ = −0.61 eV) with a lower activation energy, compared with the formation of H_2_COO (*E*_a_ = 0.95 eV and *E*_r_ = +0.35 eV). The further process involving HCOOH reacting with HCO to H_2_COOH (*E*_a_ = 0.42 eV and *E*_r_ = −0.62 eV), has a moderate activation energy, which is also the case for HCOOH direct hydrogenation to H_2_COOH (*E*_a_ = 0.36 eV and *E*_r_ = +0.13 eV). H_2_COOH subsequently dissociates thermoneutrally to yield CH_2_O (*E*_a_ = 0.44 eV and *E*_r_ = 0.00 eV) with a lower activation energy, and further processes from CH_2_O to methane formation have been shown above.

In summary, the DFT results suggest that Rh(111) can facilitate the H_2_ dissociative adsorption to surface atomic H species, which can react with activated CO_2_ and CO for further hydrogenation. The RWGS reaction appears to occur predominantly via the redox mechanism (i.e., the dissociation of the adsorbed CO_2_ to the adsorbed CO). However, the most favorable pathway for methane formation appears to be via the HCOO and HCOOH intermediates, with CO_2_ hydrogenation to HCOO having an energy barrier of 0.69 eV and reaction energy of −0.13 eV, which is then followed by further hydrogenation to HCOOH (*E*_a_ = 0.87 eV and *E*_r_ = +0.61 eV). HCOOH dissociation to yield HCO is slightly endothermic and has a modest activation barrier (*E*_a_ = 0.42 eV and *E*_r_ = +0.09 eV). The adsorbed HCO can subsequently undergo hydrogenation to HCOH (*E*_a_ = 0.69 eV and *E*_r_ = +0.56 eV), followed by its dissociation to CH (*E*_a_ = 0.47 eV and *E*_r_ = −0.72 eV), which can be hydrogenated to the ultimate product methane. It should be noted that the intermediate HCO can either hydrogenate to HCOH or dissociate to CO. Given the numerous competing pathways, the extent to which other mechanisms contribute to methane formation remains unclear. Kinetic Monte Carlo techniques, however, can elucidate many subtleties in complex reaction mechanisms that are not obvious after initial analysis of the DFT results. Hence, we will return to this topic and discuss the competition between these mechanistic pathways as revealed by the kinetic Monte Carlo simulations in the corresponding section later.

### Methanol Formation, Desorption, and Decomposition

As discussed in the preceding section, many of the intermediates relevant to the various mechanistic pathways for CO_2_ methanation are common to methanol formation. Methanol formation via the hydrogenation of CH_2_OH is only modestly endothermic and has a moderate activation energy (CH_3_OH, *E*_a_ = 0.73 eV and *E*_r_ = +0.15 eV). Similarly, methanol can also result from CH_3_O hydrogenation with a comparable reaction energy and activation barrier (*E*_a_ = 0.78 eV and *E*_r_ = +0.05 eV). Hence, it is likely that elementary processes leading to the formation of methanol on the Rh(111) surface are feasible. As such, it is of interest to consider next the fate of any methanol molecules that may be formed.

Clearly, there are two possibilities: either methanol can desorb to the gas phase, or undergo some dissociation process. While methanol desorption is endothermic by 0.67 eV, the dissociation of methanol to CH_2_OH (i.e., the reverse of the process for CH_2_OH hydrogenation discussed in the preceding paragraph), is exothermic by −0.15 eV and has a lower activation energy, 0.58 eV. Conversely, methanol dissociation to CH_3_ and OH is highly activated (*E*_a_ = 1.67 eV and *E*_r_ = −0.35 eV). Hence, it is highly likely that methanol decomposition to CH_2_OH will out-compete the desorption of methanol to the gas phase. As discussed in the previous section pertaining to the HCO and COH mechanisms for methane formation, the dissociation of CH_2_OH to CH_2_ and OH is exothermic, by −0.58 eV, although this process has a slightly higher activation barrier of 0.75 eV compared to reforming methanol (*E*_a_ = 0.73 eV). Hence, it appears likely that over longer time scales, CH_2_OH dissociation to CH_2_ will predominate, since methanol desorption is more activated than methanol dissociation to CH_2_OH, and the CH_2_OH dissociation process is more exothermic (by −0.58 eV) to yield CH_2_, which undergoes further hydrogenation to the final product CH_4_. Furthermore, the alternative dissociation of CH_2_OH to HCOH has a lower activation energy of 0.38 eV, which is exothermic by −0.21 eV. HCOH can then dissociate to CH with an activation energy of 0.47 eV, and the subsequent activation energies for CH successive hydrogenation are low (no greater than 0.64 eV for CH successive hydrogenation to CH_4_, which is lower than the effective activation barrier of 1.20 eV for reforming CH_2_OH from CH), and the corresponding reaction energies are all endothermic, suggesting that, as noted above, CH can undergo successive hydrogenation to CH_4_. Unlike methanol, methane can easily desorb to the gas phase, since the desorption energy (0.20 eV) is lower than the activation barriers for the reverse of the methane formation processes already discussed. Hence, the computational results presented above offer a potential explanation for why methane is observed to be a significant CO_2_ hydrogenation product instead of methanol over the Rh surface, based on the reaction mechanisms explored herein.

### kMC simulations

It is clear from the discussion above that the product distribution is controlled by a complex balance between activation, reaction, and desorption energies. To explore further the distribution of products and reaction mechanism, we performed kMC simulations including all elementary steps under the experimental operating conditions. The kMC simulations involve a gas mixture consisting of H_2_ and CO_2_ in a 4:1 ratio over the clean Rh(111) surface at two different temperatures: first at 473.15 K and a pressure of 1 bar, corresponding to typical experimental conditions,^52,107^ and second at 573.15 K, to investigate the impact of temperature on product selectivity.

#### Gas Products and Reaction Mechanism

**Figure 5:**
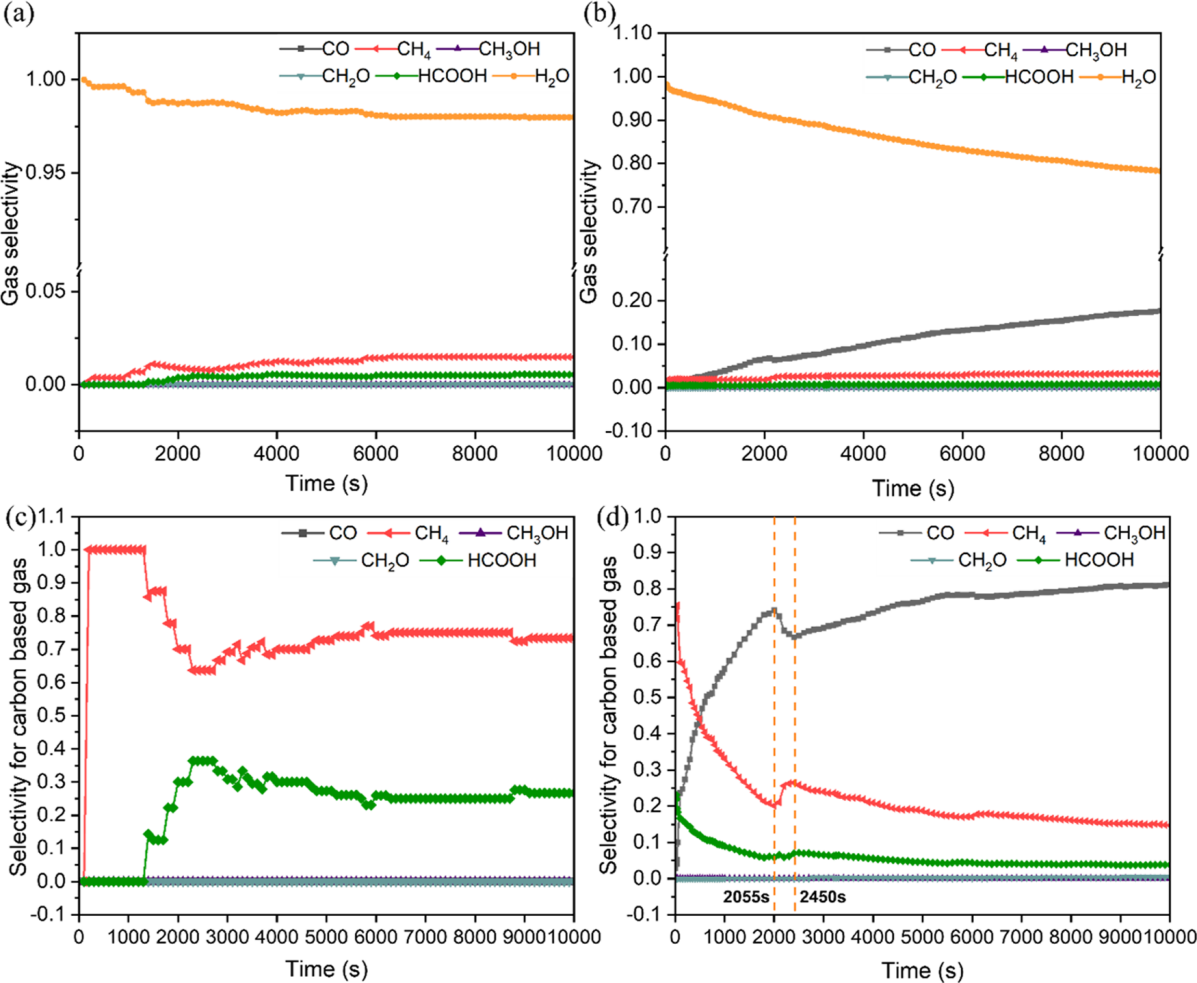
Time evolution of the gas selectivity from the CO_2_ hydrogenation to C1 products under the temperatures of (a) 473.15 K and (b) 573.15 K, with a pressure of 1 bar (*P*_H_2__ = 0.8 bar, *P*_CO_2__ = 0.2 bar). Time evolution of the selectivity for carbon-based gas under the temperatures of (c) 473.15 K and (d) 573.15 K, with a pressure of 1 bar (*P*_H_2__ = 0.8 bar, *P*_CO_2__ = 0.2 bar).

The results show that under these conditions, gaseous H_2_O, CH_4_, and HCOOH are evolved as products, with H_2_O predominating (Figure 5a,b). The evolution of significant quantities of H_2_O without a corresponding amount of C-containing products can be rationalized by the strong binding of CO to the Rh(111) surface, and thus the high CO desorption energy, which is supported by the high coverage of CO on the adlayer configurations in Figure S8. Hence, under these conditions, the RWGS reaction predominates.

**Figure 6:**
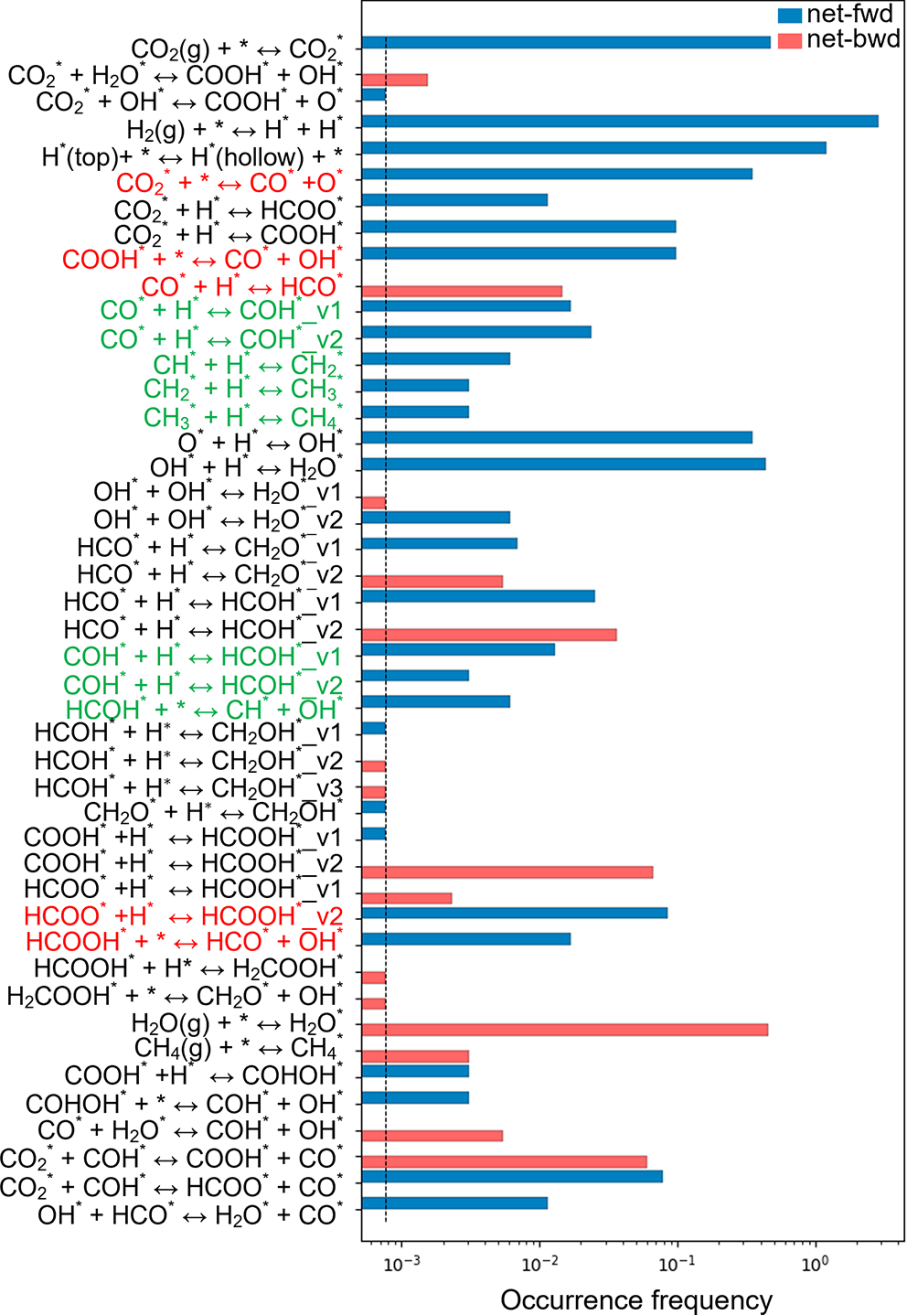
Occurrence frequency of the elementary steps (excluding events with zero frequency) during the time interval of 0–1300 s at a temperature of 473.15 K and a pressure of 1 bar (*P*_H_2__ = 0.8 bar, *P*_CO_2__ = 0.2 bar). Net rates of the reversible events are calculated by subtracting the reverse rates from the forward rates. The positive net rates are denoted as “net-fwd”, while the negative ones are labeled as “net-bwd”. Pathways for the RWGS reaction are highlighted in red, while pathways leading to methane formation are marked in green. Labels v1 and v2 represent the sets of neighboring sites with different types of site connectivity defined in kMC simulations, on which the same elementary process takes place.

The selectivity for evolved carbon-containing gases is shown in Figure 5c,d. At 473.15 K, the selectivity to methane is initially 100% and reaches the steady-state value of 75.9% around 5900 s. While the selectivity to methane begins to decrease from a kMC time of 1300 s, gaseous HCOOH emerges concurrently, with no gaseous CO being evolved at any point. To explore the gas product distribution in more detail, we plotted the occurrence frequency of all of the elementary steps between the time intervals 0–1300 s (Figure 6) and 1300–2600 s (Figure S9). Figure 6 shows that all three pathways identified are potentially feasible means to produce adsorbed CO, including direct dissociation of CO_2_, and the carboxyl and formate mechanisms (via formic acid), in agreement with the rationalization of the DFT simulations discussed previously. The resulting adsorbed CO predominantly undergoes hydrogenation, first to COH and subsequently to HCOH, rather than desorbing to the gas phase; while the DFT calculations show that the CO hydrogenation process to yield COH has a high activation barrier (+1.27 eV) and is moderately endothermic (+0.71 eV), the barrier is lower than that for HCO formation (+1.35 eV), which is considerably more endothermic (+1.21 eV). Furthermore, the activation barrier for COH formation is lower than both the activation barriers for reverting back to CO_2_ (+1.43 eV) and CO desorption (+2.28 eV). Hence, the process statistics and persistent CO surface coverage reported from the kMC simulation are consistent with the DFT calculations, despite the high activation barrier for COH formation. Furthermore, the process statistics show that formic acid produced via the formate route tends to dissociate to HCO and OH, with most HCO in turn dissociating to yield CO, and some forming HCOH or formaldehyde. Hence, the CO_2_ direct dissociation mechanism, and formate and carboxyl mechanisms, all ultimately converge at the formation of adsorbed CO and HCOH species. The process statistics also show that most of the HCOH formed dissociates to yield CH, which can be sequentially hydrogenated to the product methane. Hence, adsorbed CO is the central intermediate for CO_2_ methanation, leading to further hydrogenation to COH and HCOH, being pivotal steps in methane production.

**Figure 7:**
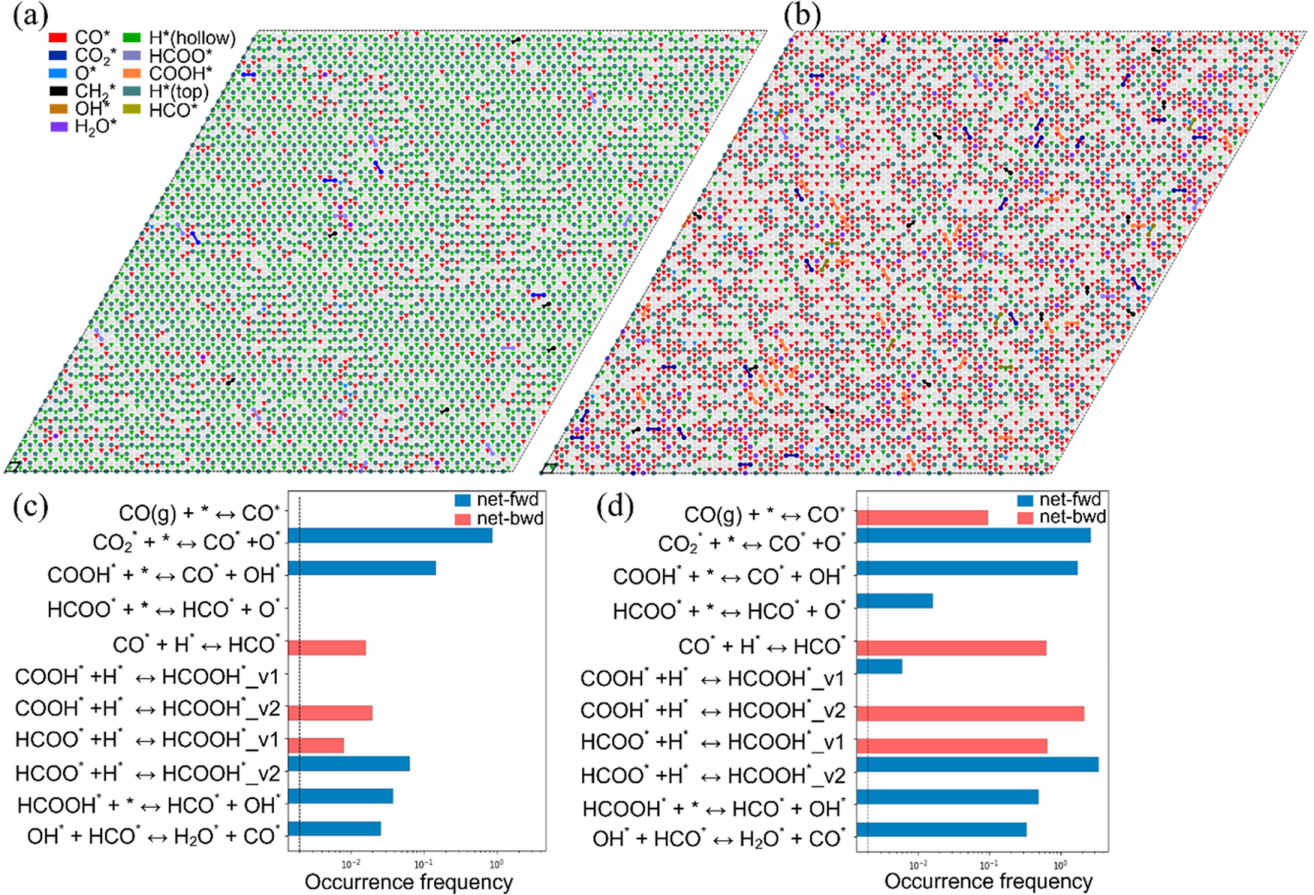
Adlayer configurations for the 50 × 50 lattice at a time of 500 s under the different temperatures: (a) *T* = 473.15 K, (b) *T* = 573.15 K. The occurrence frequency of the elementary steps (excluding events with zero frequency) over the time interval of 0–500 s under the different temperatures: (c) *T* = 473.15 K, (d) *T* = 573.15 K. The partial pressures for H_2_ and CO_2_ are 0.8 and 0.2 bar, respectively. Net-fwd and net-bwd are defined as earlier mentioned, as well as the labels v1 and v2.

The emergence of HCOOH evolution as illustrated in the kMC product distribution after ∼1300 s (Figure 5c) can be understood in terms of CO coverage. By comparing the event frequency between both time intervals (i.e., before and after 1300 s), it can be seen that desorption of gaseous HCOOH after 1300 s correlates with a greater formation and subsequent dissociation of HCO (as shown in Figure S9), which results in a higher surface coverage of CO species. The adlayer configurations in Figure S8 also show that more CO species occupy the surface at the steady-state time of 5900 s, compared with that at 500 s in Figure 7. Hence, at longer kMC simulation times, larger numbers of surface CO species can ultimately block the surface sites, thus preventing further HCOOH decomposition on the surface; HCOOH therefore desorbs to the gas phase when the surface coverage of CO is sufficiently high. This is furthermore supported by the exploration of the effect of increasing the ratio of H_2_/CO_2_ gas mixtures, which will be discussed in more detail later. Experimental results also reported that the presence of CO in the gas stream markedly inhibited the HCOOH decomposition reaction.^108^ Moreover, the process of HCOO hydrogenation to HCOOH becomes more pronounced at the time interval of 1300–2600 s (Figure S9), compared with that during 0–1300 s (Figure 6). Hence, formic acid slowly desorbs to the gas phase after 1300 s with a moderate desorption energy of 0.79 eV.

As discussed in the previous section, it is likely that the DFT-calculated CO adsorption energy represents an overestimation; hence, it is instructive to test the sensitivity of the kMC product selectivity with respect to CO binding energy. Additional simulations were performed with less exothermic CO adsorption energies to assess the impact of possible overestimation of the binding energy, as detailed in Section S7 of Supporting Information. These simulations reveal that the methane formation mechanism remains largely unchanged, while CO desorption is somewhat accelerated, leading to a higher fraction of CO being desorbed to the gas phase, as would be expected. Hence, the additional simulations validate the key features of the model applied in this study.

#### Temperature Effects

The impact of temperature on reaction mechanism and selectivity has also been considered by performing the kMC simulations at two different temperatures, 473.15 and 573.15 K, both under a pressure of 1 bar. Increasing temperature results in the production of gaseous CO as shown in Figure 5, in agreement with previous experiment,^109^ implying that elevated temperatures are required to facilitate CO desorption from the surface. Methane selectivity decreases within the time interval of 0–2055 s, whereas the selectivity of CO increases noticeably. To gain further insight into the product distribution at elevated temperatures, the adlayer configurations during the reaction process are visualized and compared at the temperatures of 473.15 and 573.15 K. Figure 7 shows the adlayer configurations after running the kMC simulation for 500 s. At 473.15 K, the lattice is predominantly covered with adsorbed H atoms, while adsorbed CO species tend to form islands, presenting a high ratio of H to CO coverages on the lattice. In contrast, higher concentrations of adsorbed CO species are observed on the surface at 573.15 K, and the H atoms adsorbed at the top sites are surrounded by at least two adsorbed CO molecules. This arrangement can hinder the H diffusion from the top sites to the hollow sites, further preventing the H_2_ dissociation at the top sites.

Our findings are in good agreement with experimental observations, which indicate that CO species adsorbed at the metal sites limit H_2_ dissociation.^110^ This is further supported by the finding that the partial kinetic order of CO at relatively high CO concentrations is negative.^54^ The elevated temperature accelerates the formation of CO, resulting in an enhanced presence of CO species, both on the surface and in the gas phase. The higher coverage of the adsorbed CO species on the Rh surface can impede the hydrogen dissociation and adsorption, thereby hindering further CO hydrogenation processes, leading to CO accumulation on the surface and its slow desorption to the gas phase. Figure 7 shows that the elevated temperature accelerates CO formation via CO_2_ and COOH dissociation pathways (with one way to obtain COOH being HCOOH dehydrogenation). The most favorable pathway under elevated temperature is HCO dehydrogenation, along with HCO derived from the promoted dissociation of HCOO and HCOOH intermediates. The prohibitive CO coverage under reaction conditions has also been reported to account for the lower activity of smaller Rh particles in CO hydrogenation.^37^ It was also reported that CO_2_ methanation appeared to be inhibited by CO on Rh/γ-Al_2_O_3_ catalyst.^28^ Overall, an increased coverage of adsorbed CO species on the Rh surface can be detrimental to hydrogen dissociative adsorption, which is an essential prerequisite for CO_2_ methanation.

#### Impact of H:CO Ratio

**Figure 8:**
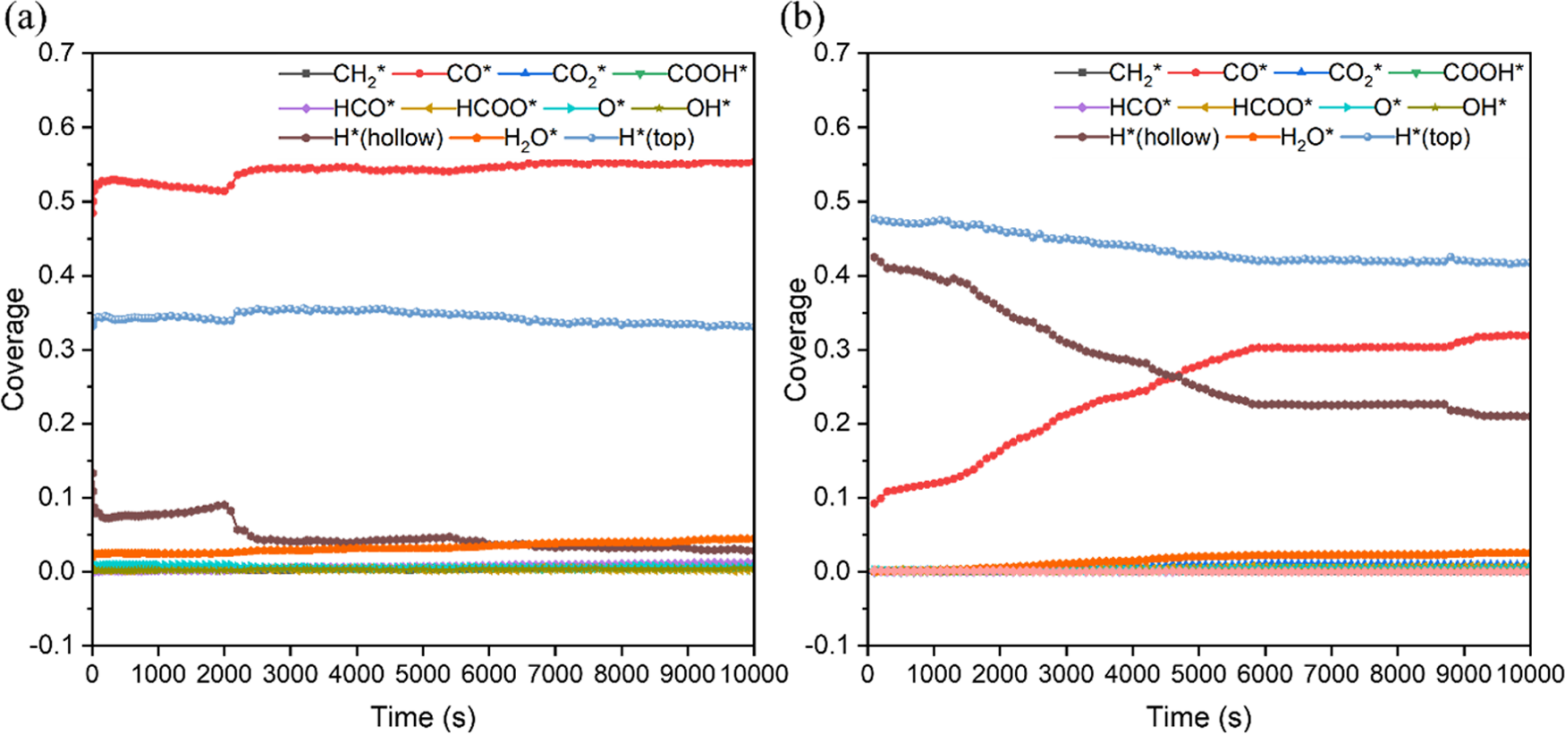
Evolution of the coverages of surface species over time at the temperatures of (a) 573.15 K and (b) 473.15 K, with a pressure of 1 bar (*P*_H_2__ = 0.8 bar, *P*_CO_2__ = 0.2 bar). Herein, the coverage is defined as the fraction of a specific surface species over the total species, independent of the number of the sites, since the different types of active sites are occupied by the multidentate species.

The coverages of H and CO species can influence the pathways controlling the selectivity of methane and CO, which is evident from the occurrence frequencies of all of the elementary events obtained from the kMC simulations. Figure 5d shows an increase in methane selectivity accompanied by a decrease in CO selectivity during the period of 2055 and 2450 s at 573.15 K. To analyze the selectivity trend, event frequencies for the time interval of 1660–2055 and 2055–2450 s (representing the opposite selectivity trends) are compared and shown in Figure S10. This can be attributed to the difference in the occurrence frequency for CO hydrogenation to COH, which occurs more frequently between 2055 and 2450 s. As discussed above, CH species result from the dissociation of the adsorbed HCOH, the formation of which in turn depends on the rate of COH formation. Hence, the higher rate of methane formation between 2055 and 2450 s is correlated with the increasing frequency of COH formation. Indeed, between 1660 and 2055 s, the net rate (defined as the forward rate minus the reverse rate) for the HCOH dissociation is close to the dashed vertical line (corresponding to the occurrence of a single event in the entire duration of the simulation), meaning that only a few events occur. However, from 2055 to 2450 s, the dissociation of HCOH occurs more frequently, as the concentration of HCOH species increases due to the higher COH formation frequency. Meanwhile, Figure 8a shows a decline in hydrogen coverage at hollow sites from 2055 to 2450 s at 573.15 K. Conversely, the coverage of the adsorbed CO increases, which is shown by the adlayer configurations, with a higher substantial ratio of H to CO coverages at 2055 s than 2450 s (Figure S11). Hence, the relative coverages of the H and CO species on the Rh(111) surface can play a decisive role in the CO hydrogenation to COH, with higher COH formation frequencies being observed for optimal H:CO surface coverages. The H:CO surface coverage ratio can also explain the different selectivities observed at different temperatures. The ratio of hydrogen adsorbed at the hollow sites and the adsorbed CO is 0.15 at the initial state and decreases to 0.06 at the steady state at 573.15 K (Figure 8a). However, at 473.15 K, the H:CO coverage ratio is significantly different, starting at 4.62 and sharply declining to 0.75 at the steady state (Figure 8b), which is considerably greater than the H:CO coverage ratio at steady state at 573.15 K. Hence, CO_2_ methanation is favored at 473.15 K, and gaseous CO is produced at 573.15 K, due to the higher surface H coverage at the steady state at 473.15 K (Figure S8). Additionally, the production of gaseous H_2_O under 573.15 K decreases over time (Figure 5b). This is because the H_2_O species can desorb to the gas phase once they are formed via RWGS reaction, while the evolution of gaseous CO occurs until the surface coverage of CO is sufficiently high due to the high desorption energy of CO. Along with CO species accumulated on the surface slowly desorb to the gas phase, gaseous H_2_O constitutes a decreasing fraction of the total gaseous species.

Based on the effects of the relative coverages of H and CO species discussed above, we considered the effect of the gaseous H_2_ to CO_2_ ratio by performing simulations with 3:1, 4:1, and 9:1 H_2_/CO_2_ gas mixtures under typical experimental conditions of 473.15 K and 1 bar. Under these conditions, methane and formic acid were the only carbon-contained species evolving to the gas phase in these simulations. The selectivity to methane was promoted with a higher H_2_ to CO_2_ ratio in the mixture, which is in agreement with previously reported thermodynamic analysis.^111,112^ This trend is confirmed, as shown in Table 3, which demonstrates that as the H_2_/CO_2_ gas mixture ratio increases, the steady-state coverage of the CO species decreases, whereas the steady-state coverage of the H species adsorbed at the hollow sites increases (θ_CO_ = 0.410, θ_H_ = 0.129 for 3:1 H_2_/CO_2_ mixture, θ_CO_ = 0.319, θ_H_ = 0.210 for 4:1 H_2_/CO_2_ mixture, and θ_CO_ = 0.180, θ_H_ = 0.348 for 9:1 H_2_/CO_2_ mixture).

**Table 3:** Selectivity of the Carbon-Based Gas Products, and the Coverage of the Surface Species over the Rh(111) Surface under Various Reaction Conditions^a^

	*T* = 473.15 K, *P*_H_2__ = 0.75 bar, *P*_CO_2__ = 0.25 bar	*T* = 473.15 K, *P*_H_2__ = 0.8 bar, *P*_CO_2__ = 0.2 bar	*T* = 473.15 K, *P*_H_2__ = 0.9 bar, *P*_CO_2__ = 0.1 bar
CH_4_ selectivity	0.654	0.759	0.882
HCOOH selectivity	0.346	0.241	0.118
θ_CO_	0.410	0.319	0.180
θ_CO_2__	0.021	0.009	0.006
θ_COOH_	0.001	0.007	0.004
θ_HCOO_	0.009	0.005	0.004
θ_O_	0.004	0.004	0.001
θ_H_ (hollow)	0.129	0.210	0.348
θ_OH_	0.001	0.002	0.001
θ_H_2_O_	0.047	0.026	0.014
θ_H_ (top)	0.367	0.417	0.440

a Coverage is defined as earlier mentioned.

In addition, Table S12 shows that higher pressures can enhance methane selectivity at a given reaction temperature with a higher ratio of H/CO coverage, in agreement with previous reports.^111−113^ Furthermore, the rates of CO_2_ hydrogenation, expressed as the turnover frequency (TOF), were obtained through simulations at varied temperatures with a pressure of 1 bar and a 4:1 H_2_/CO_2_ gas mixture. The apparent activation energy for the overall process was derived via the Arrhenius equation. Figure S13 shows that the calculated apparent activation energy is 19.94 kcal/mol between temperatures of 473.15 and 573.15 K, which is comparable with experimental measurements. Bell and co-workers^107^ obtained an apparent activation energy of 16.6 kcal/mol for CO_2_ hydrogenation on Rh/SiO_2_ catalyst under a fixed pressure of 608 Torr for H_2_ and 152 Torr for CO_2_. In addition, Bell, Somorjai, and co-workers^114^ reported a value of 17.0 kcal/mol by investigating methane formation for both the bare Rh surface and titania-promoted Rh surface at atmospheric pressure with a gaseous H_2_/CO_2_ ratio of 3. Meanwhile, other studies reported 16.2, 17.3, and 19.4 kcal/mol for the apparent activation energy of CO_2_ hydrogenation over Rh catalyst supported by alumina, silica, and titania, respectively,^115^ confirming that our calculated activation energy is in close agreement with experiments.^52,107,114,115^

### Comparison of CO_2_ Hydrogenation Performance over Rh and Cu Catalysts

Our computational results show that CO_2_ chemisorption on the Rh(111) surface is exothermic, with −0.33 eV of adsorption energy, which contrasts with the adsorption energies reported for the same species over different metal surfaces. DFT calculations by Higham et al.^93^ showed that the bent CO_2_ adsorbate is only metastable on low-index Cu surfaces, with endothermic adsorption energies of 0.10 and 0.05 eV reported for Cu(110) and (100) surfaces, respectively, whereas no such adsorption mode was identified for the Cu(111) facet. Kowalec et al.^92^ similarly reported a chemisorption energy of 0.09 eV for the bent CO_2_ species on Pd(111), corroborating experiments that demonstrated the absence of CO_2_ chemisorption on this surface facet. The difference in stability of the bent CO_2_ species on different metal surfaces may lie in the different stabilities of the surfaces; the calculated surface energies for Cu(111) and Pd(111) are 1.29 J/m^2^^116^ and 1.72 J/m^2^,^92^ respectively, which are lower than our calculated value of 2.85 J/m^2^ for Rh(111). Higher surface energies may promote stronger adsorption interactions due to the inherent instability of the surface facet, resulting in exothermic chemisorption of CO_2_ on the Rh(111) surface.

The combined DFT and kMC simulation results presented predict that methane is a significant CO_2_ hydrogenation product instead of methanol over the Rh surface. In terms of reaction mechanisms explored, the adsorbed CO is an essential intermediate, which facilitates further hydrogenation into COH. HCOH derived from COH hydrogenation can be dissociated into CH, which undergoes further hydrogenation to yield methane. In contrast, Cu catalysts are reported as the dominant active constituent for effective synthesis of methanol from CO_2_ hydrogenation and have been extensively studied.^117,118^ For unsupported Cu catalysts, methanol desorption is less activated than methanol dissociation, as shown by DFT calculations of CO_2_ hydrogenation on Cu(100) and (110) surfaces.^93^ Furthermore, the mechanisms of methanol synthesis on Cu catalysts have been discussed via either formate pathway^119,120^ or carboxyl pathway.^121,122^ Yang et al.^123^ proposed CO produced by the fast RWGS reaction did not undergo subsequent hydrogenation to methanol over Cu catalysts, but instead simply accumulated as a product, which was demonstrated by both experiments and calculations. The desorption energy of 0.79 eV for CO from Cu(111) also indicates that CO potentially undergoes desorption.^124^ In the kinetic regime of CO_2_ hydrogenation, an inverse kinetic isotope effect of H/D substitution on Cu/ZnO/Al_2_O_3_ catalyst was observed, which is stronger for methanol synthesis than for CO formation, suggesting that the two reactions do not share a common intermediate.^125^ Hence, methanol synthesis on Cu catalysts can be achieved without CO subsequent hydrogenation and dissociation, whereas CO is a significant intermediate for methane formation on Rh catalysts, which is indicated by the kMC results in the present work. In contrast, previous studies focusing on CO_2_ hydrogenation over Cu(100)^83^ suggested that the key process involved CO_2_ hydrogenation to formate, leading to the formation of formic acid and methanol, with few CO_2_ species undergoing dissociation into CO; COH derived from CO hydrogenation was found to be unstable on Cu(100) surface, and consequently minimal HCOH was expected to be present, unlike that for Rh(111) where HCOH is a key intermediate leading to the formation of CH species, and ultimately methane. In addition, HCOOH readily desorbs from Cu(100) and is only weakly bound at low surface coverages, whereas for Rh(111), HCOOH dissociates to HCO, which undergoes further dehydration to CO, thus limiting methanol production via HCOOH hydrogenation and favoring methane formation via HCOH as discussed above.

## Summary and Conclusions

Our DFT simulations have shown that the Rh catalyst promotes CO_2_ activation and dissociation, as well as H_2_ dissociation, as demonstrated by the geometric and electronic-structure analysis of the adsorption structures for CO_2_ and H_2_ molecules, along with low activation energies for H_2_ dissociation. The RWGS reaction can proceed via three possible mechanisms: the redox mechanism, the carboxyl mechanism, and the formate mechanism, via formic acid. Analysis of the DFT results suggests that methane formation is favored by CO_2_ direct hydrogenation to formate, with subsequent hydrogenation to formic acid, which dissociates to HCO. Subsequently, the dissociation of HCOH derived from HCO hydrogenation can yield CH, which undergoes further hydrogenation to yield the final product methane. However, kMC simulations demonstrate that adsorbed CO is a crucial intermediate for methane formation, undergoing hydrogenation into COH and subsequently HCOH, which itself then subsequently undergoes dissociation to CH and then hydrogenation to methane formation as indicated by DFT simulations.

The reaction temperature was found to have a profound effect on the reaction mechanism and product selectivity. Higher methane production via the Sabatier reaction was observed to take place on the surfaces with a higher H/CO ratio at 473.15 K, with no gaseous CO production observed; however, the evolution of gaseous CO starts to occur at 573.15 K, as evident from the adlayer configurations under these conditions. This finding highlights the crucial role of the H/CO ratio in controlling the product distribution in CO_2_ hydrogenation over Rh-based catalysts. The elevated temperature accelerates CO formation via the three mechanisms. Subsequently, the higher coverage of the adsorbed CO species on the Rh surface can impede hydrogen dissociation and adsorption, thereby hindering further CO hydrogenation processes. This mechanism leads to CO accumulation on the surface and eventually slow desorption to the gas phase at the elevated temperature. Hence, a higher ratio of H to CO coverage on the Rh(111) surface enhances methane formation, with the key steps being CO hydrogenation to COH and the dissociation of HCOH. The contrast with the CO_2_ hydrogenation over copper, where the selectivity toward methanol is observed, can be largely attributed to lower CO surface coverages, the instability of the COH intermediate, and thus the minimal presence of the HCOH species (a key intermediate for the Sabatier reaction, as illustrated by the present work); instead, copper favors direct hydrogenation to formate, leading to methanol formation.

In summary, the present work not only provides new insights into the mechanism of CO_2_ methanation on Rh(111) surfaces but also illustrates the value of combining different computational techniques to provide a multiscale analysis of reaction mechanisms. While DFT simulations can provide valuable insights from reaction profiles with static energies, kMC simulations can provide a deeper insight into the exploration of the reaction mechanism with a statistical and dynamical method based on the DFT-calculated reaction profile, elucidating mechanistic subtleties arising from competing processes and intermediates.

## Supporting Information Available

Further details on ZPE-corrected adsorption energies, surface energies, determining of rate constants, surface lateral interactions, electronic analysis (including density of states and crystal orbital Hamilton population), impact of the magnitude of CO adsorption energy, adlayer configurations, event frequency, pressure effects, apparent activation energy, and configurations used in cluster expansion model (PDF)
