# Supplementary material for: Multiscale Investigation of the Mechanism and Selectivity of CO2 Hydrogenation over Rh(111)
Source: ACS Catal. 2024 Mar 28;14(8):5503–19. doi: 10.1021/acscatal.3c05939 (PMC11036393; doi:10.1021/acscatal.3c05939)
Supplement: Supplementary file 1 — cs3c05939_si_001.pdf [file cs3c05939_si_001.pdf]

Supporting Information for

Multiscale investigation of the mechanism and selectivity of CO_2 hydrogenation over Rh(111)

Shijia Sun[1], Michael D Higham[1, 2], Xingfan Zhang[1], C. Richard A. Catlow[1, 2, 3, *]

[1]*Kathleen Lonsdale Materials Chemistry, Department of Chemistry, University College London, London WC1H 0AJ, United Kingdom.*

[2]*Research Complex at Harwell, Rutherford Appleton Laboratory, Harwell, Oxon, OX11 0FA, United Kingdom.*

[3]*School of Chemistry, Cardiff University, Park Place, Cardiff CF10 1AT, United Kingdom.*

[*]*c.r.a.catlow@ucl.ac.uk*

Table of Contents

S1. Zero point energy corrected adsorption energy 2

S2. Surface energy 3

S3. Cluster expansion Hamiltonian 5

S4. Rate constants 11

S5. Density of States 14

S6. Crystal Orbital Hamilton Population 15

S7. Impact of the magnitude of CO adsorption energy 16

S8. Steady-state adlayer configurations 22

S9. Event frequency at 473.15 K 23

S10. Event frequency at 573.15 K 24

S11. Adlayer configurations 26

S12. Impact of pressure on selectivity 27

S13. Apparent activation energy 28

S14. Configurations used in cluster expansion model 29

References 36

S1. Zero point energy corrected adsorption energy

Table S1. The ZPE-corrected adsorption energies for all the adsorbed species calculated using the PBE and PBE+D3 methods, with cut-off energies of 450 eV and 550 eV. Adsorption sites are also indicated.

	E_{cut} = 450 eV		E_{cut} = 550 eV	
Species	ZPE-corrected E_{ads}/eV (PBE)	ZPE-corrected E_{ads}/eV (PBE+D3)	ZPE-corrected E_{ads}/eV (PBE+D3)	Adsorption site
C	-7.51	-7.68	-7.69	hollow
CH	-6.67	-6.85	-6.86	hollow
CH_2	-4.10	-4.35	-4.34	top, hollow
CH_3	-1.69	-2.03	-2.02	hollow
CH_4	0.00	-0.20	-0.20	top
CO	-1.99	-2.28	-2.28	hollow
CO_2	0.10	-0.33	-0.34	top, top, bridge
COH	-4.70	-4.95	-4.97	hollow
COOH	-2.42	-2.74	-2.76	top, bridge, top
CH_2OH	-1.71	-2.09	-2.09	top, bridge, top
CH_3OH	-0.23	-0.67	-0.66	top, hollow
HCO	-2.35	-2.62	-2.63	top, bridge, hollow
HCOH	-3.87	-3.37	-3.38	top, bridge, hollow
HCOO	-2.79	-3.15	-3.15	top, bridge, top
HCOOH	-0.40	-0.79	-0.80	top, bridge, top
H_2CO	-0.77	-1.19	-1.20	top, bridge, hollow
H_2COO	0.47	0.06	0.05	top, hollow, bridge
H_2COOH	-2.00	-2.45	-2.45	top, hollow, bridge
H_3CO	-1.99	-2.34	-2.32	bridge
O	-5.00	-5.37	-5.37	hollow
H	-2.63	-2.72	-2.72	hollow
OH	-2.74	-2.92	-2.92	bridge
H_2O	-0.23	-0.53	-0.53	top

S2. Surface energy

The surface energies (E_{surf}) of different layers of slab models were calculated using both the PBE and PBE+D3 methods. **Figure S2** shows that surface energy stops fluctuating for slabs consisting of at least six layers for both approaches. Moreover, the calculated surface energy of the Rh(111) facet in this work is presented in **Table S2**, compared with the results from previous computational and experimental studies. Hence, the 6-layer slab model was applied to the following simulations.

The energy penalty for the surface generated by cleaving a bulk (E_{cleave}) is given by the following:

$$E_{cleave} = \frac{E_{slab}^{unrelaxed} - N \cdot E_{bulk}}{2A} \tag{1}$$

where $E_{slab}^{unrelaxed}$ and E_{bulk} are the DFT total energy of an unrelated surface slab model and bulk energy of an atom, respectively. The N and A are the number of atoms in the slab model and the surface area of the slab model, respectively.

In addition, the energy of geometry relaxation (E_{relax}) is calculated by the difference in total DFT energy of the optimised slab $E_{slab}^{relaxed}$ and $E_{slab}^{unrelaxed}$, as shown in the equation (2):

$$E_{relax} = \frac{E_{slab}^{relaxed} - E_{slab}^{unrelaxed}}{A} \tag{2}$$

where the denominator is A only, since it is a single-sided surface model with bottom layers constrained.

Subsequently, the surface energy (E_{surf}) can be obtained by the following equation:

$$E_{surf} = E_{cleave} + E_{relax} \tag{3}$$

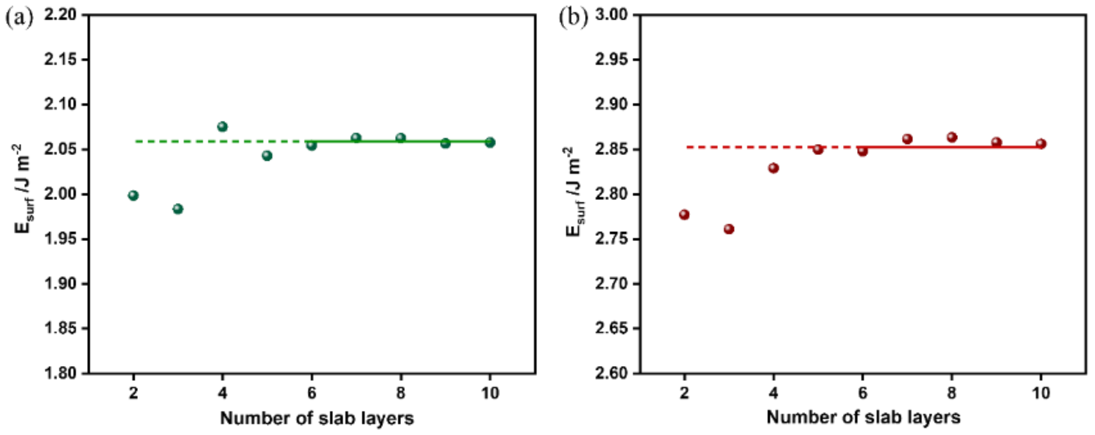

Figure S2. The calculated surface energy (E_{surf}) of Rh(111) with PBE (a) and PBE+D3 (b) methods as a function of the number of slab layers. The average surface energy (solid horizontal line) was taken from more than 6 layers, to illustrate the extent of fluctuations of surface energy.

Table S2. The calculated surface energy of Rh(111) in this work, compared with the results from previous computational and experimental studies.

**Method**	E_{surf} **/J m^{-2}**	**Ref.**
PBE	2.05	This work
PBE+D3	2.85	This work
LDA	2.54	Phys. Rev. B, 1993, 48, 1877[1]
PW91	1.99	Phys. Rev. B, 2007, 76, 233413[2]
LMTO-ASA	2.78	Phys. Rev. B, 1992, 46, 7157[3]
FP-LMTO	2.53	Phys. Rev. B, 1992, 46, 4816[4]
MEAM	2.59	Chinese Phys. 2004, 13, 1082[5]
MEAM	2.60	Phys. Rev. B, 1992, 46, 2727[6]
Experimental results	2.70	Cohesion in Metals (North-Holland, Amsterdam, 1988)[7]
Experimental results	2.66	Surf. Sci., 1977, 62, 267[8]

S3. Cluster expansion Hamiltonian

The Zacros code used in the present work provides the definition of cluster expansion Hamiltonian in terms of interactions between co-adsorbed species, which is by defining the site connectivity between adsorbates, and the corresponding cluster energy. The total energy of the adlayer configuration can be adjusted as a function of surface coverage dynamically during kMC simulations, by detecting surface adsorption patterns automatically.

The total energy (E_i) of the lattice configuration i can be expressed as:

$$E_i = \sum_{n=1}^{k} N_n \cdot CE_n \tag{4}$$

where N_n and CE_n are the number of times for the appearance of the cluster n, and the energy of cluster n, respectively. The k parameter is the total number of clusters included in the system. The values for two-body interactions are listed in **Table S3-1**.

Table S3-1. The ZPE-corrected lateral interaction energies for most relevant co-adsorbed species on the Rh(111) surface.

Co-adsorbed species	Lateral interaction energy/eV	Co-adsorbed species	Lateral interaction energy/eV
H-H	0.22	CH_3-H	0.28
CO-O	0.27	CO_2-H	0.26
C-O	0.26	CO-OH	0.01
CO-H	0.08	COOH-H	0.07
COH-H	0.06	HCO-OH	0.18
C-OH	0.18	HCOOH-H	0.57
HCO-H	0.15	H_2CO-OH	-0.02
CH-O	0.19	HCO-O	0.15
H_2CO-H	0.15	HCOO-H	0.02
CH_2-O	0.26	H_2COO-H	-0.13
CH-OH	0.23	CH_2OH-H	0.05
HCOH-H	0.00	H_3CO-H	0.02
CH_2-OH	0.24	CH_3-OH	0.48
CH_3-O	0.33	O-H	0.34
C-H	0.13	OH-H	0.05
CH-H	0.07	OH-OH	-0.05
CH_2-H	0.02	H_2O-O	-0.15

CO_2-OH	-0.03	COOH-O	-0.04
CO_2-H_2O	-0.16	HCOO-OH	0.09
COOH-OH	-0.08	COH-OH	0.13
CO-H_2O	-0.19	CO-CO	0.19
CO_2-C	0.41	CO_2-COH	-0.29
COOH-CO	-0.05	HCOO-CO	0.008
HCOO-HCO	0.001	HCOOH-CO	0.25
H_2COO-CO	-0.007	HCOOH-HCO	0.53
H_2COOH-CO	-0.07	CH_2O-HCO	0.12
CH_3OH-CO	-0.006	CH_2OH-CO	-0.07
CH_3O-HCO	-0.05	CH_3OH-CO	-0.25
CH_2OH-HCO	-0.09		

In the present work, clusters corresponding to the reactant and product co-adsorbed species have been defined in **Table S3-1**, comprising the interactions of CO with CO, O, H, OH, H_2O, CH_3O, COOH, H_2COO, H_2COOH, HCOO, HCOOH, CH_2OH, CH_3OH. The kMC results show that under the investigated conditions, adlayer configurations typically consist of high CO coverage; hence, we additionally consider the interactions between CO and the remaining adsorbates, and investigate the impact of the additional interactions on the results. The values of the lateral interaction energies of CO with the other species are summarised in **Table S3-2**.

Table S3-2. ZPE-corrected lateral interaction energies for co-adsorbed CO with remaining species on the Rh(111) surface.

**Co-adsorbed species**	**Lateral interaction energy/eV**	**Co-adsorbed species**	**Lateral interaction energy/eV**
CO-C	0.224	CO-COH	-0.002
CO-CH	0.147	CO-HCO	0.114
CO-CH_2	0.336	CO-HCOH	-0.135
CO-CH_3	0.319	CO-H_2CO	-0.080
CO-CO_2	0.186		

Following kMC simulations including the additional interactions as detailed in **Table S3-2**, we observe the same selectivity trends and similar surface coverages as those obtained without the supplements of these lateral interactions, as shown in **Table S3-3**. No gaseous CO evolution emerges at the steady state. The percentage of HCOOH in gas products increases after including more interactions, which may be due to the slightly increased CO coverage, as discussed in the analysis of the gaseous HCOOH evolution in the manuscript. In addition.

Figure S3-1 shows the event frequency from the beginning of the simulation until the emergence of the steady state for kMC simulations including the supplementary lateral interactions detailed in **Table S3-2**. Reaction mechanisms for RWGS (highlighted in red) and methane formation (highlighted in green) are consistent with the analysis in the manuscript. Hence, it can be seen that the inclusion of the additional clusters corresponding to lateral interactions between CO and all other species does not have significant impact on the reaction mechanism and product distribution, and thus the findings of the present work.

Table S3-3. Selectivity of the carbon-based gaseous products, and the coverage of the surface species over the Rh(111) surface with and without the interactions of CO with the other species. Operating conditions: T=473.15 K, P_{H_2}=0.8 bar, P_{CO_2}=0.2 bar.

	**No additional interactions**	**Additional interactions**
CH_4 selectivity	0.759	0.667
CO selectivity	\	\
HCOOH selectivity	0.241	0.333
θ_{CO}	0.319	0.324
θ_{CO_2}	0.009	0.003
θ_{COOH}	0.007	0.010
θ_{HCO}	0.002	0.001
θ_{HCOO}	0.005	0.008
θ_{COH}	0.000	0.000
θ_{CH_2}	0.002	0.002
θ_{O}	0.004	0.004
θ_{H}(hollow)	0.210	0.206
θ_{OH}	0.002	0.001
θ_{H_2O}	0.026	0.027
θ_{H}(top)	0.417	0.414

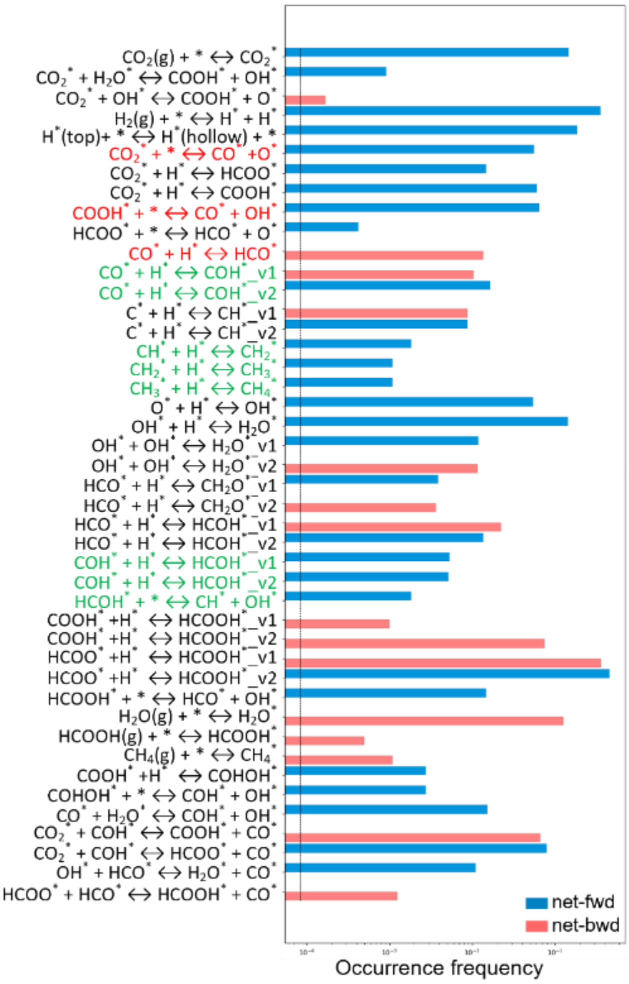

Figure S3-1. Occurrence frequency of the elementary steps (excluding events with zero frequency) including the supplements of lateral interactions from the beginning of the simulation to the steady state at a temperature of 473.15 K and a pressure of 1 bar (P_{H_2}=0.8 bar, P_{CO_2}=0.2 bar).

Furthermore, to justify the cluster expansion approach applied in the present work, we employed configuration comprising a range of different CO coverages. DFT calculations were performed using the p(3 × 3) slab model with varying number of CO species. **Figure 3-2** shows the energy difference between that determined as a sum of cluster energies, and that calculated explicitly from the DFT. The energy difference is less than 0.02 eV per CO adsorbate for higher CO coverage, demonstrating that the two-body cluster expansion approach implemented in the present work is sufficient. As shown in **Figure 3-3**, compact CO adsorption configurations (all CO molecules adsorbed at the neighboring sites) at lower coverages imply more CO-CO pairwise interactions at the neighboring sites than sparse CO configurations, leading to an over-estimation of repulsive interactions between CO molecules. Hence, the energy difference becomes positive and larger at lower CO coverage, which can compensate the over-binding energy of CO to some extent. At higher CO coverage, some CO molecules adopt adsorption configurations at bridge sites for after DFT optimization, reducing CO-CO pairwise interactions at the neighboring hollow sites. Thus, the adlayer energy determined as a sum of

cluster energies is under-estimated when including fewer CO-CO pairwise interactions than expected. In addition, H atoms will move to keep away from each other when adding more CO molecules at the adjacent sites, thus the DFT optimized configurations will reduce the repulsion between H atoms and become more stable, which accounts for the positive energy difference. In general, the cluster expansion model closely matches the DFT calculation results, although some DFT optimized configurations have disordered adsorption states.

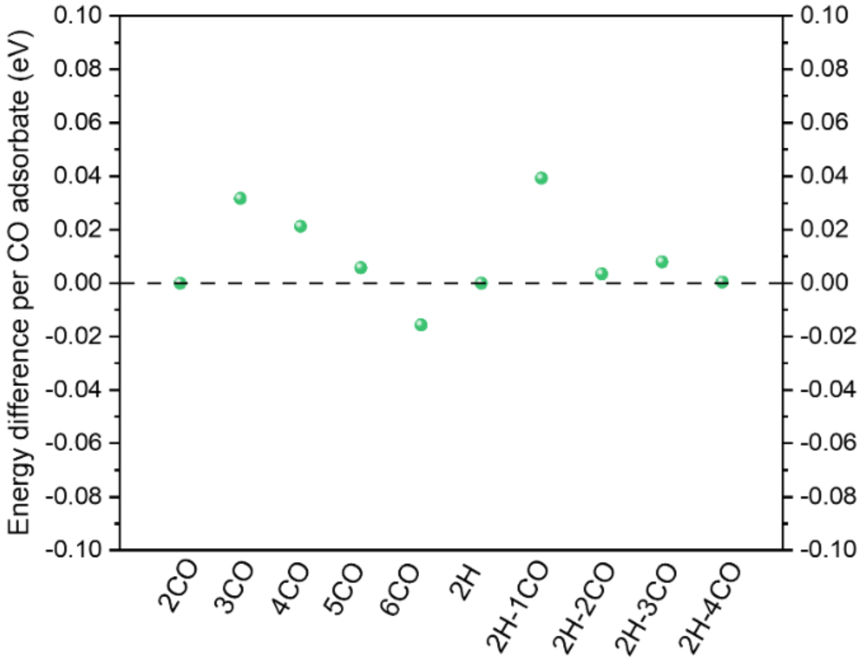

Figure S3-2. Energy difference for the configurations between cluster expansion model predicted energy and DFT calculated energy. Positive values indicate that the sum of cluster energies overestimates repulsive interaction energies, leading to less stable adlayer configurations compared to the explicit DFT calculation results. Negative values indicate the opposite.

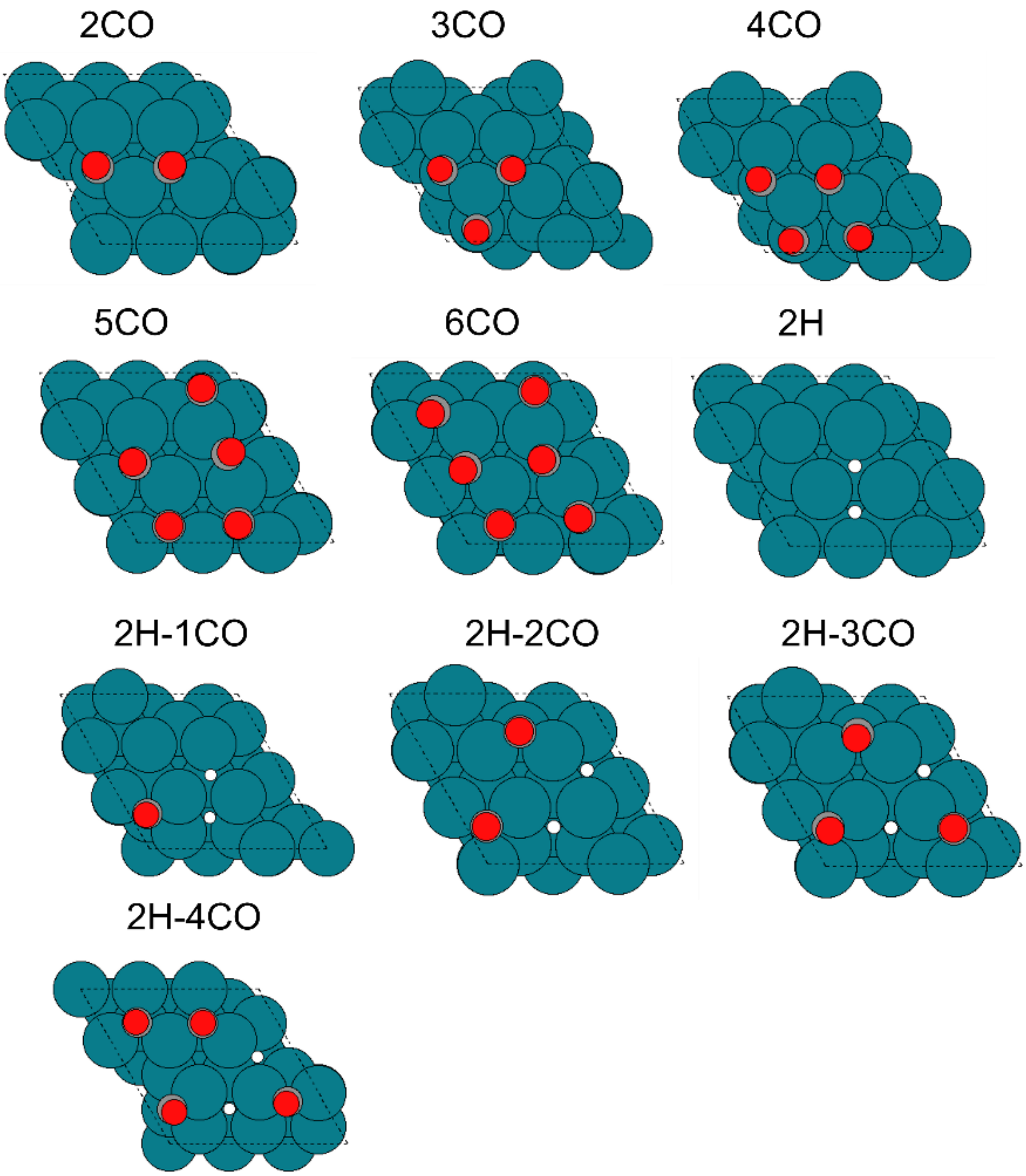

Figure S3-3. DFT optimised configurations used to demonstrate the reliability of cluster expansion model.

S4. Rate constants

The reaction kinetics were determined by implementing DFT-calculated activation energies for all events in the kMC model. For the surface reactions, the Zacros code determines the forward rate constant k_{fwd} from the Arrhenius equation:

$$k_{fwd} = \frac{q_{vib}^{TS}}{q_{vib}^{R}} \frac{k_B T}{h} \exp\left(-\frac{E_{fwd}}{k_B T}\right) \tag{5}$$

where q_{vib}^{TS} and q_{vib}^{R} are the vibration partition functions of the transition states and reactants, respectively. k_B is the Boltzmann constant. T, temperature, h, the Planck constant, and E_{fwd}, the activation barrier in the forward reaction step.

For the backward step, the backward rate constant k_{bwd} follows the equation:

$$k_{bwd} = \frac{q_{vib}^{TS}}{q_{vib}^{P}} \frac{k_B T}{h} \exp\left(-\frac{E_{bwd}}{k_B T}\right) \tag{6}$$

where q_{vib}^{P} is the vibration partition function of the products, and E_{bwd}, the activation barrier in the backward reaction step, E_{fwd}-$E_{bwd} = \Delta E$.

For the surface reactions involving gaseous species (such as Eley-Rideal (ER) reactions) or the activated adsorption process, the rotational and translational partition function of the gas species should be considered:

$$k_{fwd} = \frac{q_{vib}^{TS}}{q_{vib}^{sur} q_{vib}^{gas} q_{rot}^{gas} q_{trans}^{gas}} \frac{pA}{\sqrt{2\pi m k_B T}} \exp\left(-\frac{E_{fwd}}{k_B T}\right) \tag{7}$$

p is the pressure; A is the effective area of the reaction site and m is the mass. Adsorption of CO_2 and dissociative adsorptions of H_2 can be considered as ER reactions.

For the non-activated adsorption, $E_{fwd} = 0$, the rate constant is calculated by the following expression:

$$k_{ads} = \frac{pA}{\sqrt{2\pi m k_B T}} \tag{8}$$

For the activated desorption, the rate constant should be calculated by:

$$k_{des} = \frac{q_{vib}^{TS}}{q_{vib}^{sur}} \frac{k_B T}{h} \exp\left(-\frac{E_{bwd}}{k_B T}\right) \tag{9}$$

In addition, the rate constant for the non-activated desorption can be expressed by:

$$k_{des} = \frac{q_{vib}^{gas} q_{rot}^{gas} q_{trans}^{gas}}{q_{vib}^{sur}} \frac{k_B T}{h} \exp\left(\frac{\Delta E_{ads}}{k_B T}\right) \tag{10}$$

where ΔE_{ads} is the adsorption energy of the gas species over the catalyst surface.

Note that the partition functions are calculated using the harmonic approximation:

$$q_{vib} = \prod_i \frac{\exp\left(-\frac{h\nu_i}{2k_BT}\right)}{1-\exp\left(-\frac{h\nu_i}{k_BT}\right)} \quad (11)$$

$$q_{tran} = \frac{2\pi m k_B T A}{h^2} \quad (12)$$

For linear molecules:

$$q_{rot} = \frac{8\pi^2 I k_B T}{\sigma h^2} \quad (13)$$

For non-linear molecules:

$$q_{rot} = \frac{(\pi I_a I_b I_c)^{\frac{1}{2}}}{\sigma} \frac{8\pi^2 I k_B T^{\frac{3}{2}}}{\sigma h^2} \quad (14)$$

where I is the moment of inertia, σ is the symmetry number of the molecule.

The activation barriers obtained from DFT calculations correspond to the zero-coverage configurations, which need to be corrected to account for lateral interactions. To account for coverage effects, it is possible to explicitly construct a cluster expansion model including interactions between transition states and various adsorbates, which can constitute an accurate description of the coverage effects on activation barriers. However, due to the large number of species and elementary processes (61 reversible reaction steps) included in our reaction model, this procedure would be resource-intensive to implement. Hence, in practice, we applied the Brønsted-Evans-Polanyi (BEP) linear scaling relation to account for the impact of adsorbate lateral interaction on activation barriers, although fitting the ab initio calculated activation energies to a BEP scaling relation would introduce error, this still provides the qualitative understanding of the influence of lateral interactions on activation barriers[9].

Accounting for the effects of lateral interactions, the difference between forward and reverse activation energy is equal to the reaction energy, $\Delta E(\theta)$, which is expressed by:

$$\Delta E(\theta) = E_{fwd}(\theta) - E_{bwd}(\theta) \quad (15)$$

$\Delta E(\theta)$ can be obtained from the cluster expansion Hamiltonian model. $E_{fwd}(\theta)$ and $E_{bwd}(\theta)$ are the forward and backward activation energies at that specific configuration denoted by θ, respectively. They can be parameterized via the BEP relations.

$$E_{fwd}(\theta) = max(0, \Delta E(\theta), E_{fwd}(0) + \omega \cdot (\Delta E(\theta) - \Delta E(0)) \quad (16)$$

$$E_{bwd}(\theta) = max(0, -\Delta E(\theta), E_{bwd}(0) - (1-\omega) \cdot (\Delta E(\theta) - \Delta E(0)) \quad (17)$$

where $E_{fwd}(0)$ and $E_{bwd}(0)$ are the forward and backward activation energies at the zero-coverage limit. ω[10] is the proximity factor ranging from 0.0 for an initial-state-like transition state, to 1.0 for a finial-state-like transition state. Here, assuming that the transition state has both initial and final state character, we chose ω = 0.5 for all elementary processes in our reaction model. $\Delta E(0)$is the reaction energy at the zero-coverage limit, expressed by:

$$\Delta E(0) = E_{fwd}(0) - E_{bwd}(0) \quad (18)$$

We selected a proximity factor ω = 0.5 for all elementary steps; this is a reasonable initial selection given that proximity factor is a measure of how closely the transition state resembles the initial or final state configurations. Moreover, it would be computationally demanding to attempt to obtain accurate and strongly justified selections of proximity factors for all elementary processes. As such, in order to test the sensitivity of the kMC simulations to the choice of proximity factor ω, a further simulation with different proximity factors was performed. The ω values shown in **Table S4** were taken from Deimel et al[11]; late transition sates have ω > 0.5, and tend to have higher activation barriers, whilst for early transition states, ω < 0.5, with these processes tending to have lower activation barriers, as can be seen from **Table 2** in the main text.

Table S4. Proximity factor ω values for elementary steps[11]

**Elementary steps**	**ω**
CO + * ↔ C + O	0.792
CO + H ↔ HCO	0.562
C + H ↔ CH	0.363
CH + H ↔ CH_2	0.239
CH_2 + H ↔ CH_3	0.243
CH_3 + H ↔ CH_4	0.211
O + H ↔ OH	0.563
OH + H ↔ H_2O	0.350
Adsorption	0.000
Diffusion	0.500

Further kMC simulations performed at 473 K and 1 bar, with a ratio of 4:1 for H_2/CO_2, shows that CO desorption from the surface is slightly enhanced, with a gaseous CO selectivity of 0.077 being observed; thus, the change of ω value from 0.50 to 0.00 for the CO adsorption step can account for the higher CO desorption rate at high CO coverages. In addition, the simulation performed with the adjusted ω values as shown in **Table S4** showed no difference in turnover frequency (TOF), with a value of 0.010 s^{-1} obtained. Hence, the additional simulations show that there is no significant difference in catalytic performance and a similar reaction mechanism using the revised proximity factors obtained by Deimel et al. As such, the choice to apply a proximity factor of 0.5 to all processes can be considered a reasonable approximation in our study.

S5. Density of States

The DOS[11] analysis can provide an in-depth understanding of the orbital interactions. The results of DOS analysis for H_2 adsorption results are shown in **Figure S5**. The chemisorption of H_2 at the top site is more stable due to the appearance of the lower energy peak (E = -8 eV) in the Rh(s), Rh (d) and H(s) orbitals, when compared with the H_2 physisorption. Moreover, the interaction of the two H atoms decreases, since the energy peak of H-H separates into a higher energy peak for the H_2 chemisorption at the top site with elongation of H-H bond as it undergoes dissociation.

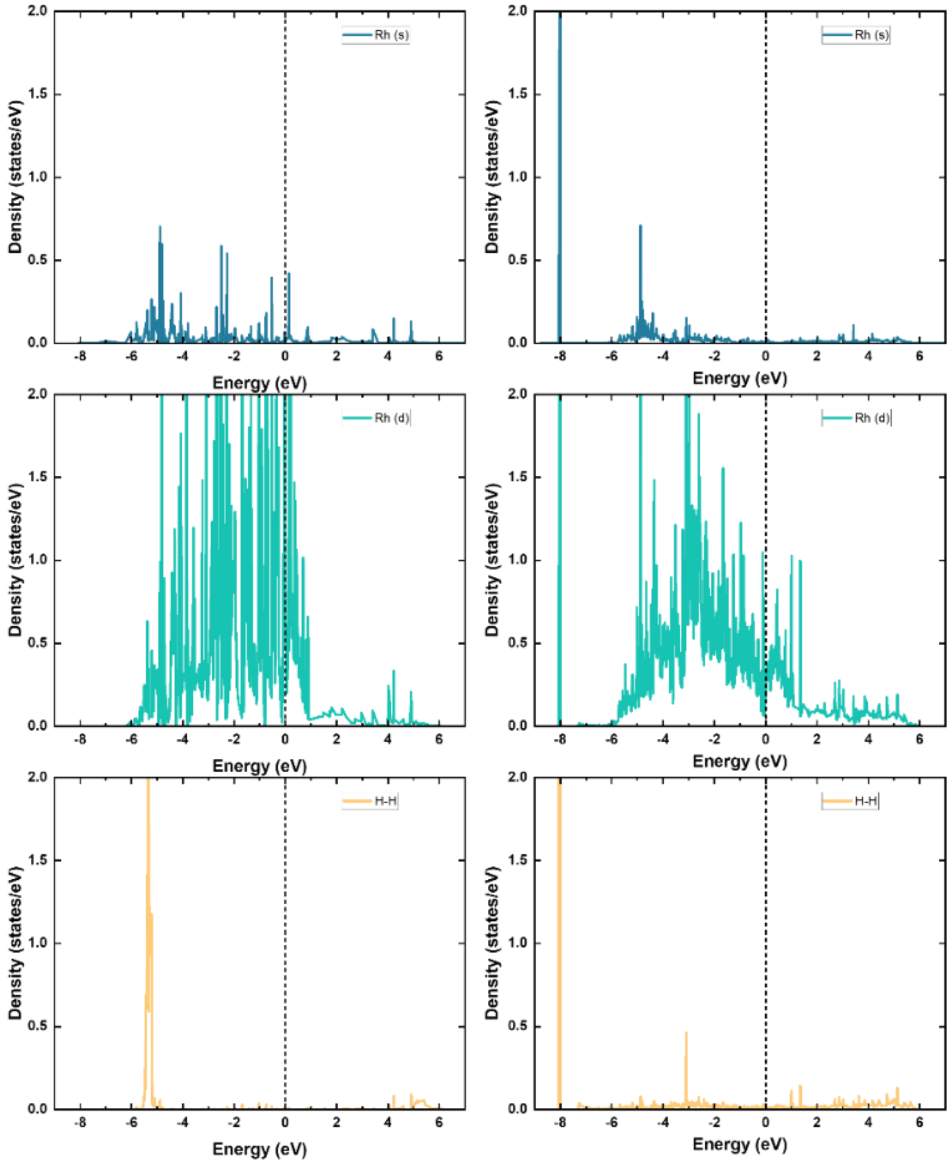

Figure S5. Analysis of the projected density of states (pDOS) for H_2 physisorption (left) and H_2 chemisorption at the top site (right). The results of the pDOS for Rh(s) orbital (top), Rh(d) orbital (middle) and H(s)-H(s) orbital (bottom).

S6. Crystal Orbital Hamilton Population

The COHP[12-14] analysis can extract chemical bonding information from the calculated electron density, such as bonding and antibonding characteristics. To explore the orbital contributions in detail, the COHP analysis was also used to characterize the interactions between the orbitals of Rh atom and H atoms. The integrated Crystal orbital Hamilton population (ICOHP) for H-H is -1.46, while ICOHP for Rh-H is -0.81. Hence, the H-H interaction is stronger than the Rh-H interaction. In addition, the results of the integrated projected Crystal orbital Hamilton population (IpCOHP) for the Rh(*s*)-H(*s*) and Rh(*d*)-H(*s*) orbital interaction are -0.16 and -0.65, respectively. Hence, the Rh(*d*)-H(*s*) orbital interaction contributes more to the bond strength between the Rh and H atoms, compared with the interaction of the Rh(*s*)-H(*s*) orbitals. The results of COHP analysis are reported in **Figure S6**.

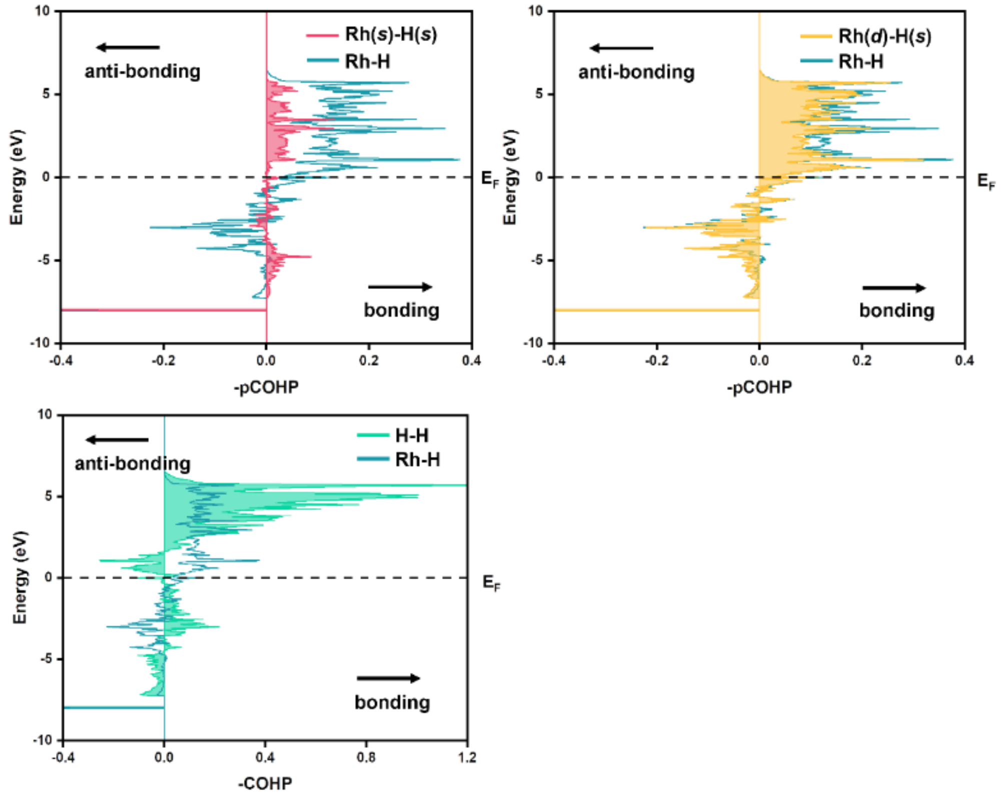

Figure S6. The analysis of projected Crystal orbital Hamilton population (pCOHP) for elongated H_2 chemisorbed at the top site. The analysis of pCOHP for interactions between the Rh(*s*) and H(*s*) orbitals (as shown in red), Rh(*d*) and H(*s*) orbitals (as shown in yellow). The analysis of COHP for bonding of Rh-H (as shown in blue) and H-H (as shown in green) atoms.

S7. Impact of the magnitude of CO adsorption energy

It is well-established that PBE functional can lead to the over-binding of CO on transition metal surfaces. Experimental adsorption energy of CO over Rh(111) surface has been reported as a value of -1.45 eV[15, 16]. To investigate the influence of the magnitude of CO adsorption energy on the product distribution and reaction mechanism, a range of CO adsorption energies from our calculation to experimental values was selected, with the adsorption energies of -2.28 eV, -1.99 eV, -1.88 eV, and -1.45 eV, respectively. After implementing these adsorption energies as energetic input in the kMC simulations, we got some results as shown below.

Table S7 shows gaseous CO species accounts for a greater proportion of the gas phase product distribution in kMC simulations where the CO adsorption energy is smaller, i.e. less exothermic. When the CO binding energy is adjusted from -2.28 eV to -1.99 eV, the higher CO surface coverage and the evolution of CO to the gas phase observed indicate that more CO is being formed, as evidenced from the higher frequency of CO_2 dissociation (**Figure S7**). In addition, methane selectivity is slightly improved, which results from the emergence of HCOH formation from CH_2OH dehydrogenation, and higher frequencies of subsequent HCOH dissociation to CH, and its further hydrogenation to final product methane. CO desorption to the gas phase also accounts for the decreasing HCOOH selectivity. However, when deceasing CO binding energy to the value of -1.88 eV, the kMC simulation results show a higher fraction of CO in the gas phase products, comparable with methane selectivity, and thus the surface coverage of CO decreases. The event frequencies have not obviously changed compared with the results from the simulation of CO adsorption energy of -1.99 eV, except for the CO desorption rate. Furthermore, CO adsorption energy of -1.45 eV is sufficiently low to promote all adsorbed CO being evolved to the gas phase, leading to high gas selectivity of CO product. Meanwhile, no HCOOH was observed in the gas phase, which agrees well with the discussion above.

The kMC simulations show that the reaction mechanisms for CO formation are the same when the CO adsorption energy is adjusted to make CO binding weaker. For methane formation, hydrogen atoms for CO hydrogenation to COH can be provided by hydrogen molecules and water molecules. As shown in **Figure S7**, CO species interact increasingly with H atoms from hydrogen dissociation to produce COH, with the CO adsorption energy of -1.88 eV and -1.45 eV as kMC energetic inputs, respectively. Hence, changing the CO adsorption energy contributes to some additional CO desorption, and thus a greater fraction of CO in the gas phase, as would be expected, but otherwise has no significant effect on the reaction mechanism of CO and methane formation.

Table S7. Selectivity of the carbon-based gas products, and the coverage of the surface species over the Rh(111) surface with different CO adsorption energies. Operating conditions: T=473.15 K, P_{H_2}=0.8 bar, P_{CO_2}=0.2 bar.

	**E_{ads}(CO)= -2.28 eV**	**E_{ads}(CO)= -1.99 eV**	**E_{ads}(CO)= -1.88 eV**	**E_{ads}(CO)= -1.45 eV**
CH_4 selectivity	0.759	0.796	0.499	0.019
CO selectivity	\	0.137	0.454	0.981
HCOOH selectivity	0.241	0.067	0.047	\
θ_{CO}	0.319	0.363	0.239	0.000
θ_{CO_2}	0.009	0.014	0.023	0.000
θ_{COOH}	0.007	0.003	0.001	0.000
θ_{HCO}	0.002	0.001	0.000	0.000
θ_{HCOO}	0.005	0.005	0.002	0.006
θ_{COH}	0.000	0.012	0.044	0.000
θ_{CH_2}	0.002	0.014	0.035	0.007
θ_{O}	0.004	0.000	0.000	0.000
θ_{H}(hollow)	0.210	0.169	0.264	0.526
θ_{OH}	0.002	0.001	0.000	0.000
θ_{H_2O}	0.026	0.052	0.035	0.000
θ_{H}(top)	0.417	0.365	0.354	0.461

(a)

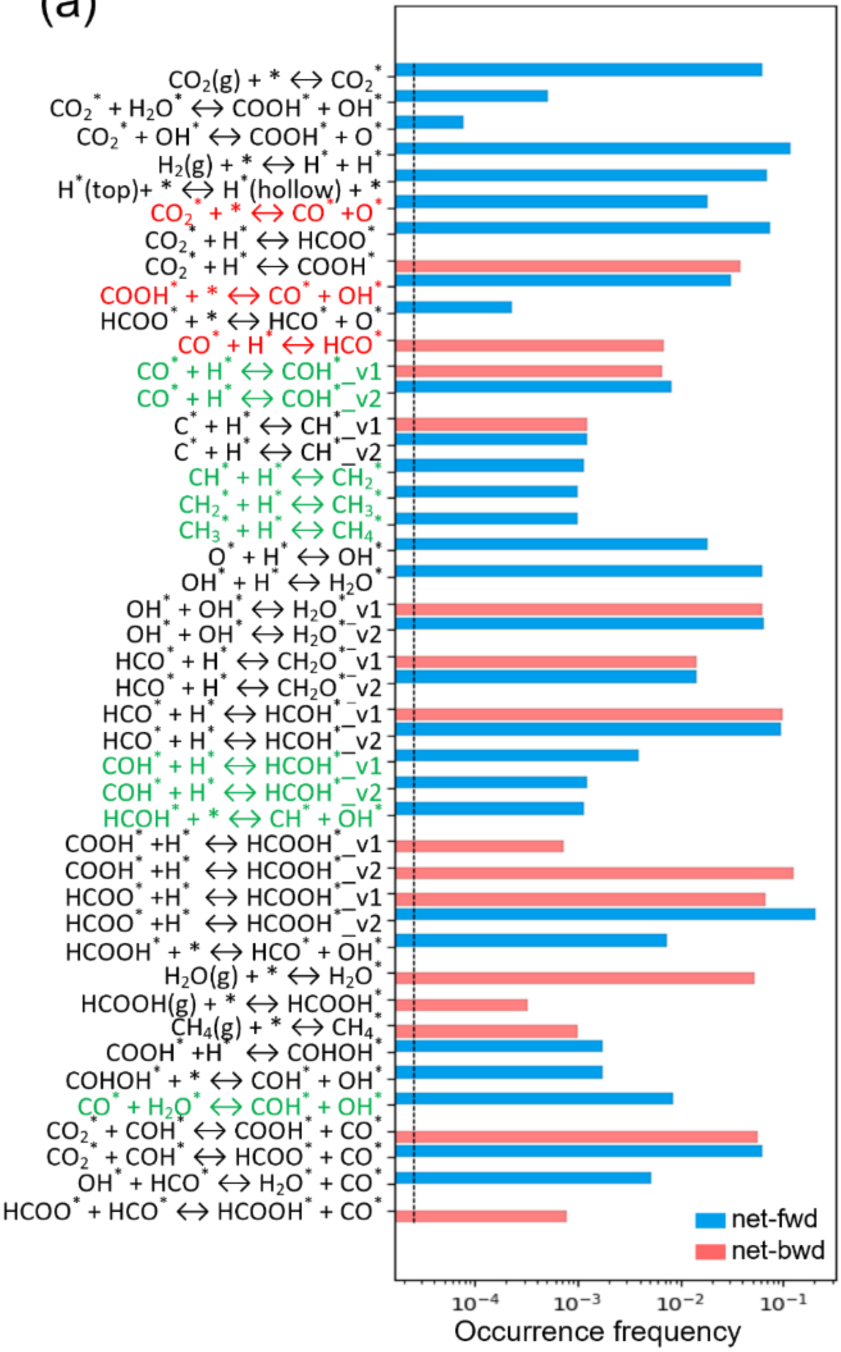

CO2(g) + * ↔ CO2*
CO2* + H2O* ↔ COOH* + OH*
CO2* + OH* ↔ COOH* + O*
H2(g) + * ↔ H* + H*
H*(top)+ * ↔ H*(hollow) + *
CO2* + * ↔ CO* +O*
CO2* + H* ↔ HCOO*
CO2* + H* ↔ COOH*
COOH* + * ↔ CO* + OH*
HCOO* + * ↔ HCO* + O*
CO* + H* ↔ HCO*
CO* + H* ↔ COH*_v1
CO* + H* ↔ COH*_v2
C* + H* ↔ CH*_v1
C* + H* ↔ CH*_v2
CH* + H* ↔ CH2*
CH2* + H* ↔ CH3*
CH3* + H* ↔ CH4*
O* + H* ↔ OH*
OH* + H* ↔ H2O*
OH* + OH* ↔ H2O*_v1
OH* + OH* ↔ H2O*_v2
HCO* + H* ↔ CH2O*_v1
HCO* + H* ↔ CH2O*_v2
HCO* + H* ↔ HCOH*_v1
HCO* + H* ↔ HCOH*_v2
COH* + H* ↔ HCOH*_v1
COH* + H* ↔ HCOH*_v2
HCOH* + * ↔ CH* + OH*
COOH* +H* ↔ HCOOH*_v1
COOH* +H* ↔ HCOOH*_v2
HCOO* +H* ↔ HCOOH*_v1
HCOO* +H* ↔ HCOOH*_v2
HCOOH* + * ↔ HCO* + OH*
H2O(g) + * ↔ H2O*
HCOOH(g) + * ↔ HCOOH*
CH4(g) + * ↔ CH4*
COOH* +H* ↔ COHOH*
COHOH* + * ↔ COH* + OH*
CO* + H2O* ↔ COH* + OH*
CO2* + COH* ↔ COOH* + CO*
CO2* + COH* ↔ HCOO* + CO*
OH* + HCO* ↔ H2O* + CO*
HCOO* + HCO* ↔ HCOOH* + CO*
net-fwd
net-bwd
10−4
10−3
10−2
10−1
Occurrence frequency

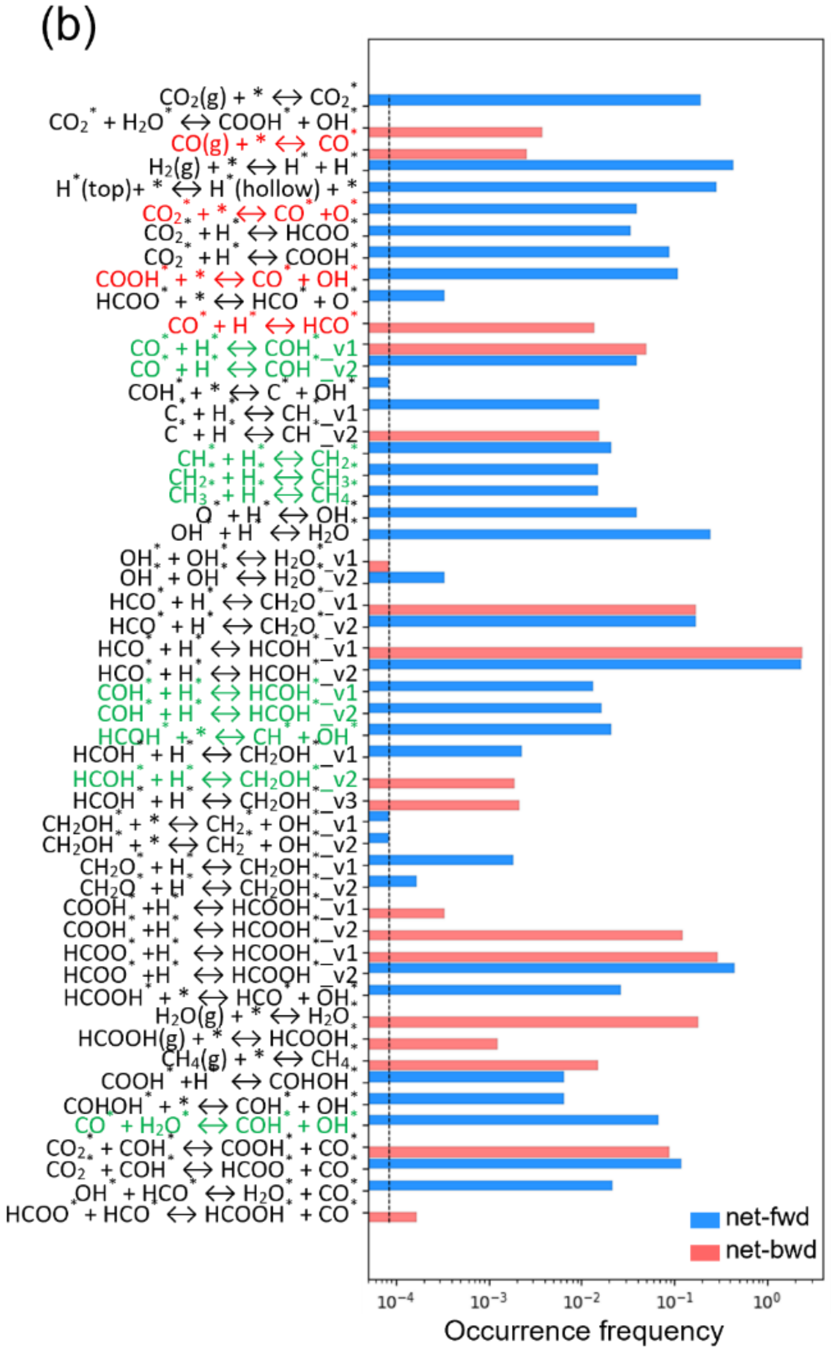
(b)
CO2(g) + * ↔ CO2*
CO2* + H2O* ↔ COOH* + OH*
CO(g) + * ↔ CO*
H2(g) + * ↔ H* + H*
H*(top)+ * ↔ H*(hollow) + *
CO2* + * ↔ CO* +O*
CO2* + H* ↔ HCOO*
CO2* + H* ↔ COOH*
COOH* + * ↔ CO* + OH*
HCOO* + * ↔ HCO* + O*
CO* + H* ↔ HCO*
CO* + H* ↔ COH*_v1
CO* + H* ↔ COH*_v2
COH* + * ↔ C* + OH*
C* + H* ↔ CH*_v1
C* + H* ↔ CH*_v2
CH* + H* ↔ CH2*
CH2* + H* ↔ CH3*
CH3* + H* ↔ CH4*
O* + H* ↔ OH*
OH* + H* ↔ H2O*
OH* + OH* ↔ H2O*_v1
OH* + OH* ↔ H2O*_v2
HCO* + H* ↔ CH2O*_v1
HCO* + H* ↔ CH2O*_v2
HCO* + H* ↔ HCOH*_v1
HCO* + H* ↔ HCOH*_v2
COH* + H* ↔ HCOH*_v1
COH* + H* ↔ HCOH*_v2
HCOH* + * ↔ CH* + OH*
HCOH* + H* ↔ CH2OH*_v1
HCOH* + H* ↔ CH2OH*_v2
HCOH* + H* ↔ CH2OH*_v3
CH2OH* + * ↔ CH2* + OH*_v1
CH2OH* + * ↔ CH2* + OH*_v2
CH2O* + H* ↔ CH2OH*_v1
CH2O* + H* ↔ CH2OH*_v2
COOH* +H* ↔ HCOOH*_v1
COOH* +H* ↔ HCOOH*_v2
HCOO* +H* ↔ HCOOH*_v1
HCOO* +H* ↔ HCOOH*_v2
HCOOH* + * ↔ HCO* + OH*
H2O(g) + * ↔ H2O*
HCOOH(g) + * ↔ HCOOH*
CH4(g) + * ↔ CH4*
COOH* +H* ↔ COHOH*
COHOH* + * ↔ COH* + OH*
CO* + H2O* ↔ COH* + OH*
CO2* + COH* ↔ COOH* + CO*
CO2* + COH* ↔ HCOO* + CO*
OH* + HCO* ↔ H2O* + CO*
HCOO* + HCO* ↔ HCOOH* + CO*
net-fwd
net-bwd
10^-4
10^-3
10^-2
10^-1
10^0
Occurrence frequency

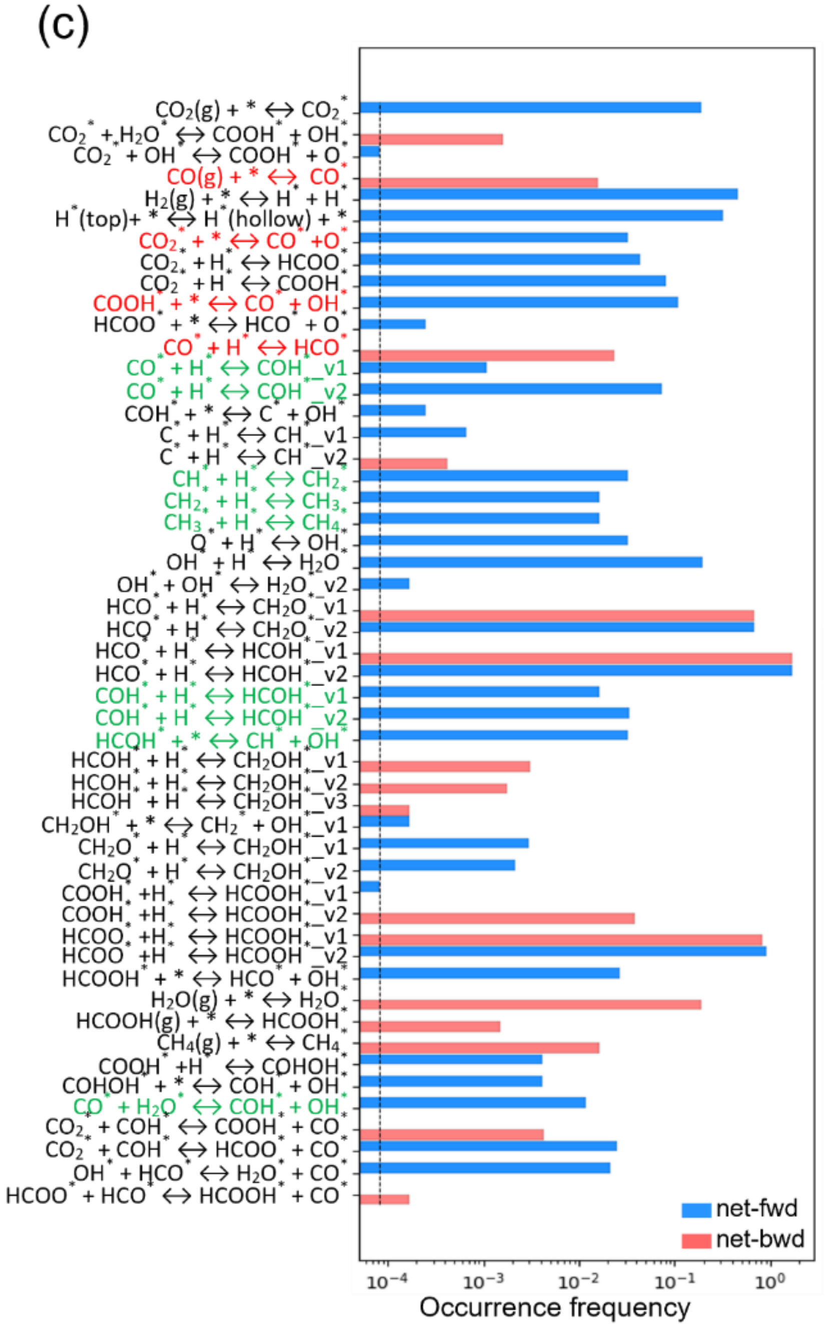

(c)
CO2(g) + * ↔ CO2*
CO2* + H2O* ↔ COOH* + OH*
CO2* + OH* ↔ COOH* + O*
CO(g) + * ↔ CO*
H2(g) + * ↔ H* + H*
H*(top)+ * ↔ H*(hollow) + *
CO2* + * ↔ CO* +O*
CO2* + H* ↔ HCOO*
CO2* + H* ↔ COOH*
COOH* + * ↔ CO* + OH*
HCOO* + * ↔ HCO* + O*
CO* + H* ↔ HCO*
CO* + H* ↔ COH*_v1
CO* + H* ↔ COH*_v2
COH* + * ↔ C* + OH*
C* + H* ↔ CH*_v1
C* + H* ↔ CH*_v2
CH* + H* ↔ CH2*
CH2* + H* ↔ CH3*
CH3* + H* ↔ CH4*
O* + H* ↔ OH*
OH* + H* ↔ H2O*
OH* + OH* ↔ H2O*_v2
HCO* + H* ↔ CH2O*_v1
HCO* + H* ↔ CH2O*_v2
HCO* + H* ↔ HCOH*_v1
HCO* + H* ↔ HCOH*_v2
COH* + H* ↔ HCOH*_v1
COH* + H* ↔ HCOH*_v2
HCOH* + * ↔ CH* + OH*
HCOH* + H* ↔ CH2OH*_v1
HCOH* + H* ↔ CH2OH*_v2
HCOH* + H* ↔ CH2OH*_v3
CH2OH* + * ↔ CH2* + OH*_v1
CH2O* + H* ↔ CH2OH*_v1
CH2O* + H* ↔ CH2OH*_v2
COOH* +H* ↔ HCOOH*_v1
COOH* +H* ↔ HCOOH*_v2
HCOO* +H* ↔ HCOOH*_v1
HCOO* +H* ↔ HCOOH*_v2
HCOOH* + * ↔ HCO* + OH*
H2O(g) + * ↔ H2O*
HCOOH(g) + * ↔ HCOOH*
CH4(g) + * ↔ CH4*
COOH* +H* ↔ COHOH*
COHOH* + * ↔ COH* + OH*
CO* + H2O* ↔ COH* + OH*
CO2* + COH* ↔ COOH* + CO*
CO2* + COH* ↔ HCOO* + CO*
OH* + HCO* ↔ H2O* + CO*
HCOO* + HCO* ↔ HCOOH* + CO*
net-fwd
net-bwd
10^-4
10^-3
10^-2
10^-1
10^0
Occurrence frequency

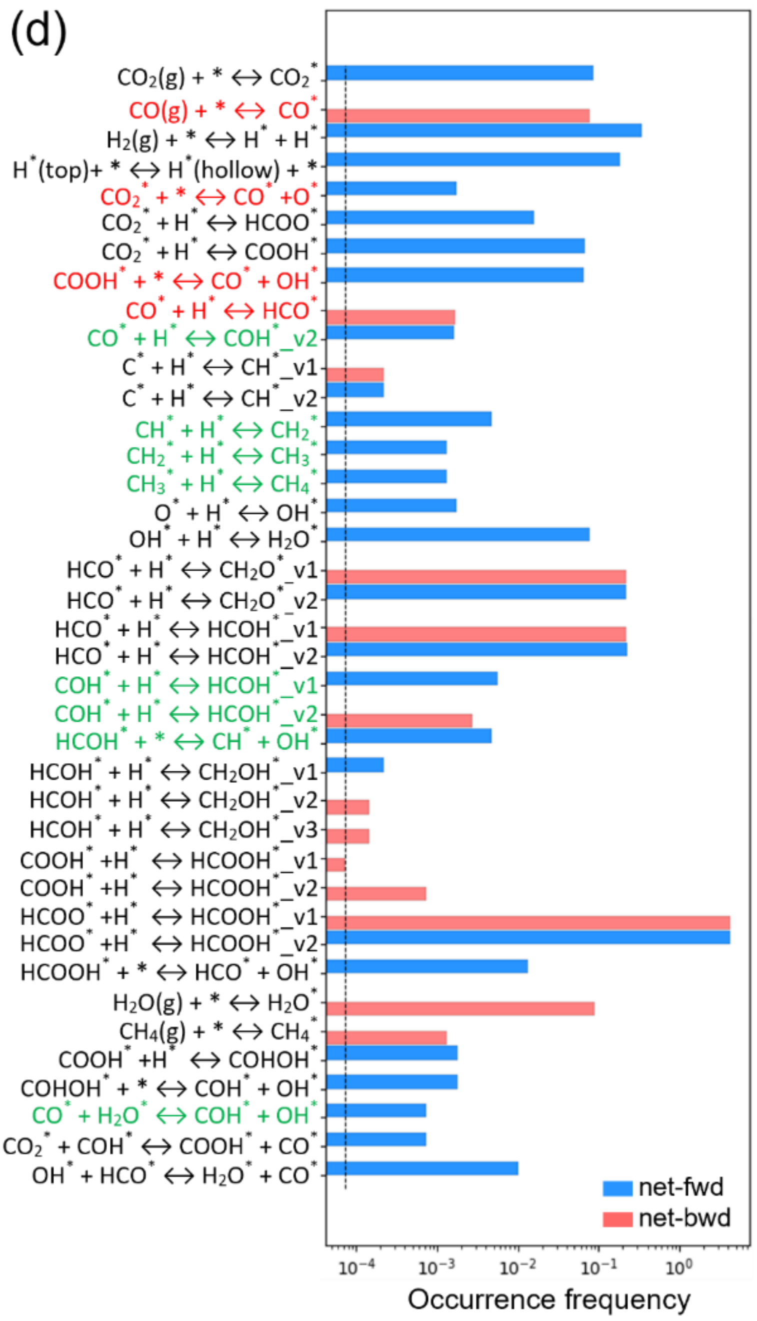

Figure S7. Occurrence frequencies for the elementary steps (excluding events with zero frequency) for different CO adsorption energies of (a) -2.28 eV, (b) -1.99 eV, (c) -1.88 eV, and (d) -1.45 eV from the beginning of the simulation to the steady state at a temperature of 473.15 K and a pressure of 1 bar (P_{H_2}=0.8 bar, P_{CO_2}=0.2 bar). Net rates of the reversible events are calculated by subtracting the reverse rates from the forward rates. The positive net rates are denoted as 'net-fwd', while the negative ones are labelled as 'net-bwd'. Pathways for the RWGS reaction are highlighted in red, while pathways leading to methane formation are marked in green. Labels v1 and v2 represent the sets of neighbouring sites with different types of site connectivity defined in kMC simulations, on which the same elementary process takes place.

S8. Steady-state adlayer configurations

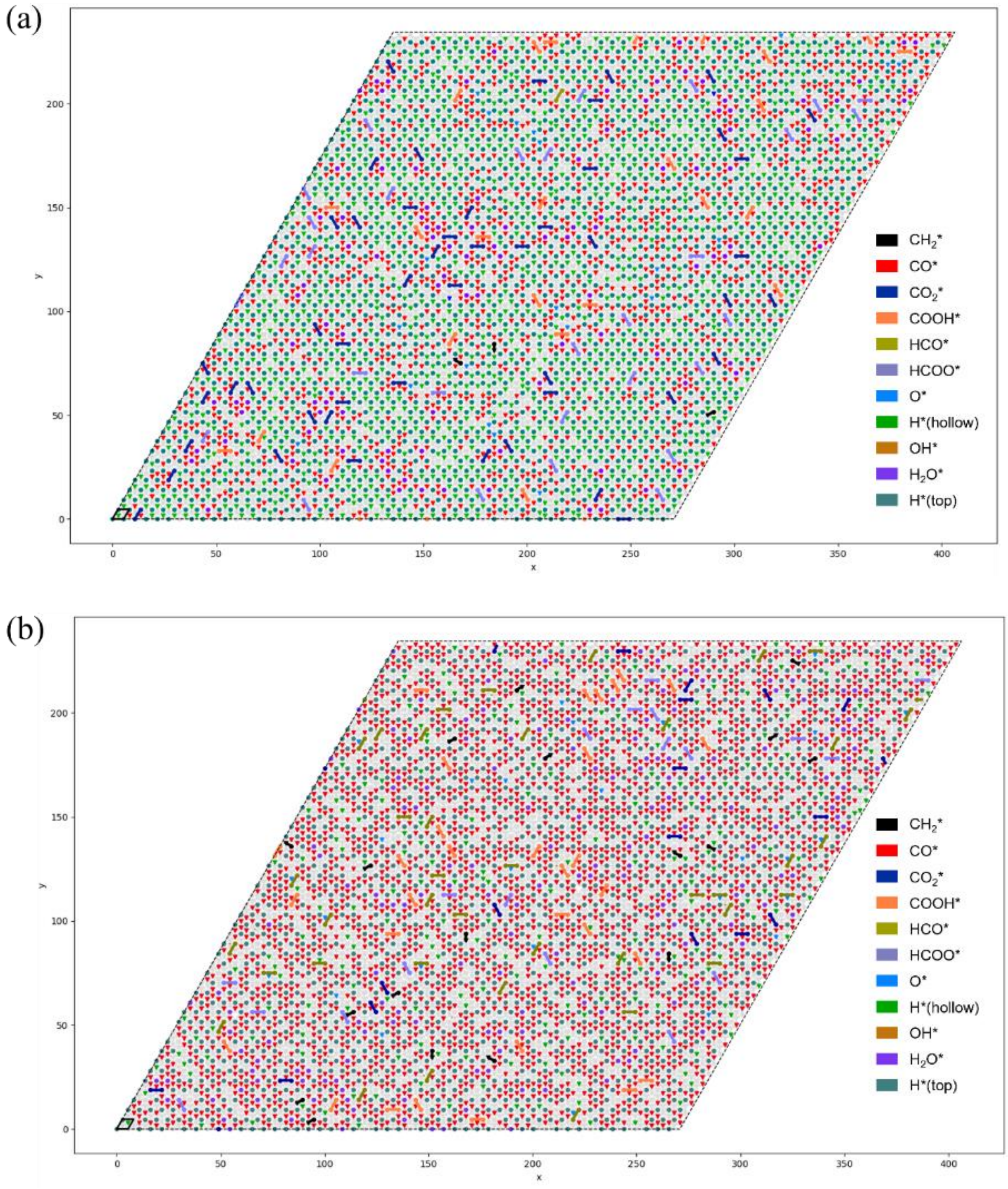

Figure S8. The adlayer configurations for the 50 × 50 lattice at the time of 5900 s at temperatures of (a) 473.15 K and (b) 573.15 K. The partial pressures for H_2 and CO_2 are 0.8 bar and 0.2 bar, respectively.

S9. Event frequency at 473.15 K

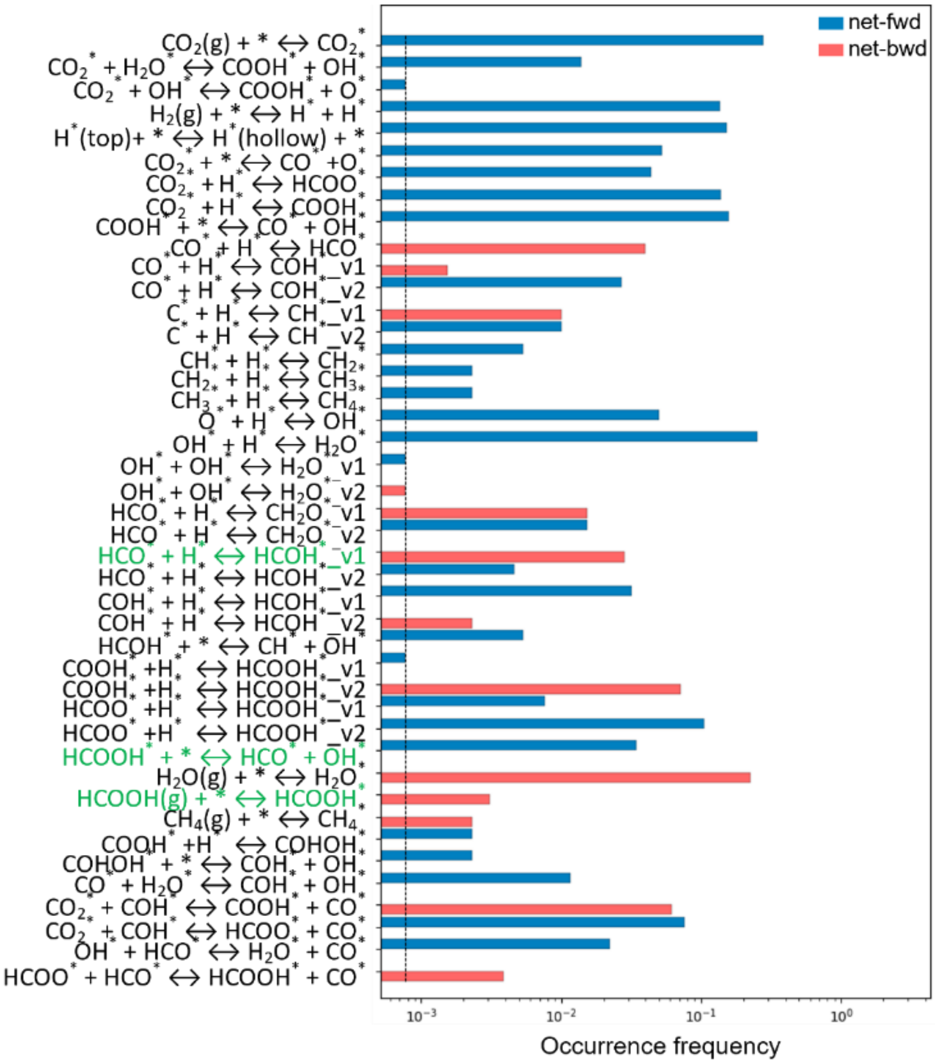

Figure S9. The occurrence frequency of the elementary steps (excluding events with zero frequency) over the time interval of 1300-2600 s at a temperature of 473.15 K and a pressure of 1 bar (P_{H_2}=0.8 bar, P_{CO_2}=0.2 bar). Pathways highlighted in green need to be noted.

S10. Event frequency at 573.15 K

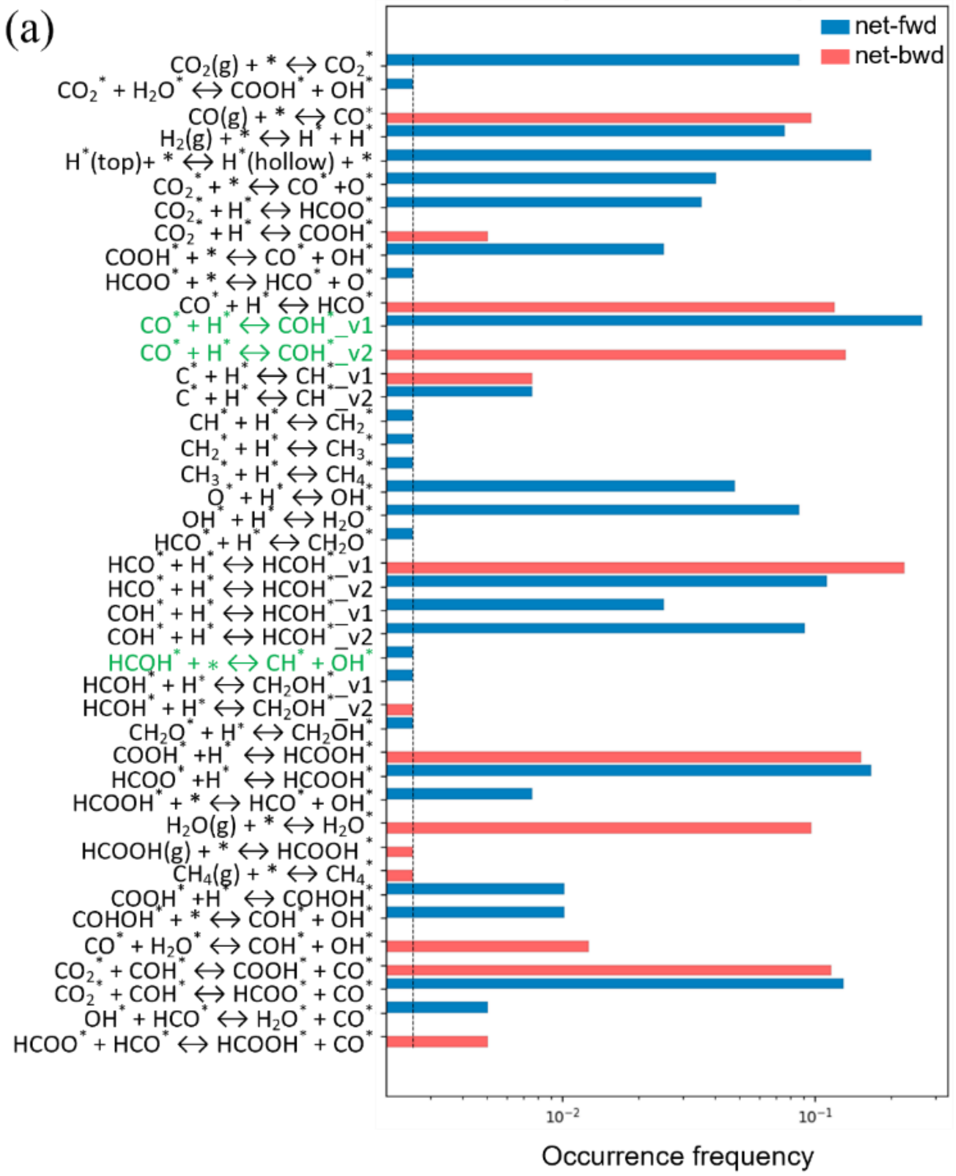

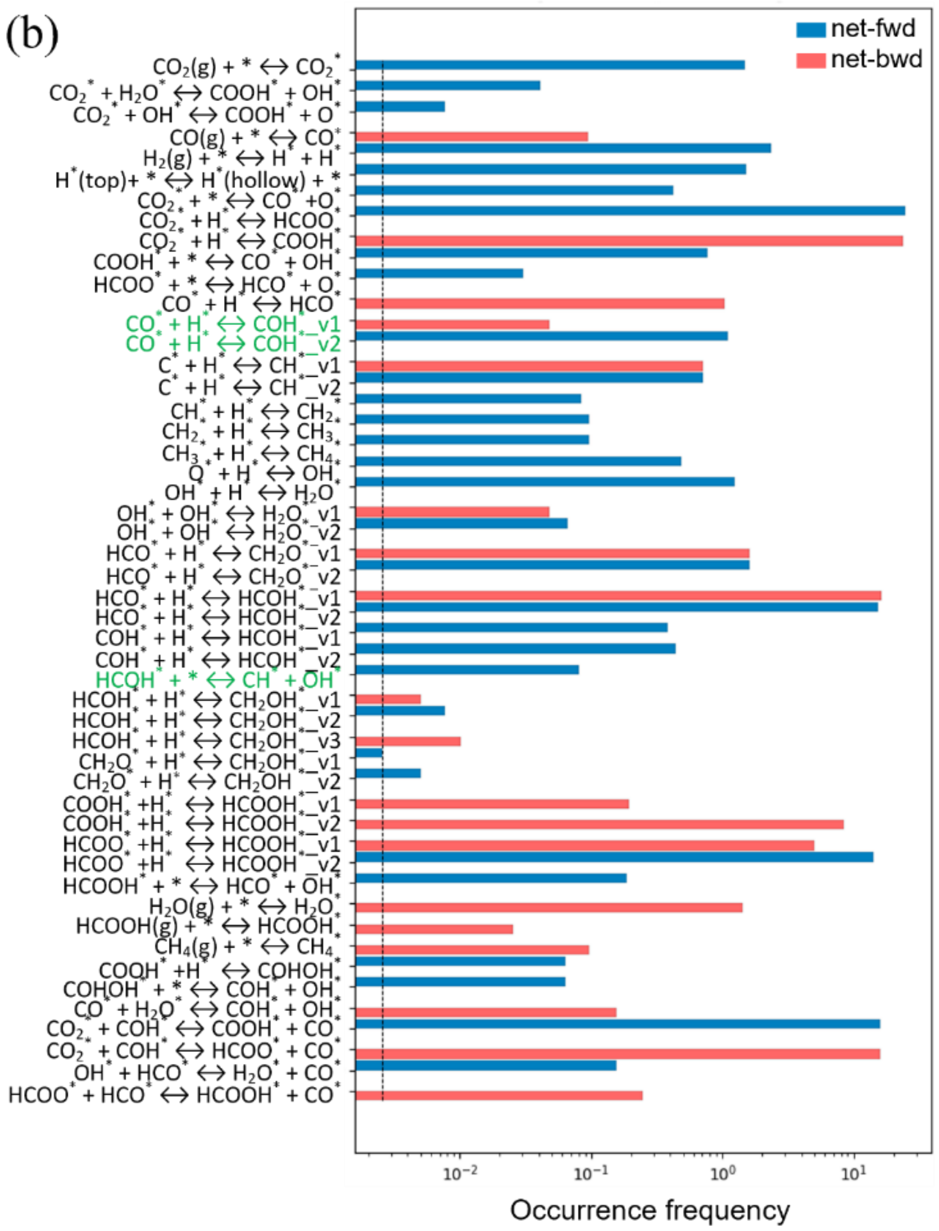

Figure S10. The occurrence frequency of the elementary steps (excluding events with zero frequency) over the time intervals of (a) 1660-2055 s and (b) 2055-2450 s at a temperature of 573.15 K and a pressure of 1 bar (P_{H_2}=0.8 bar, P_{CO_2}=0.2 bar). Pathways highlighted in green need to be noted.

S11. Adlayer configurations

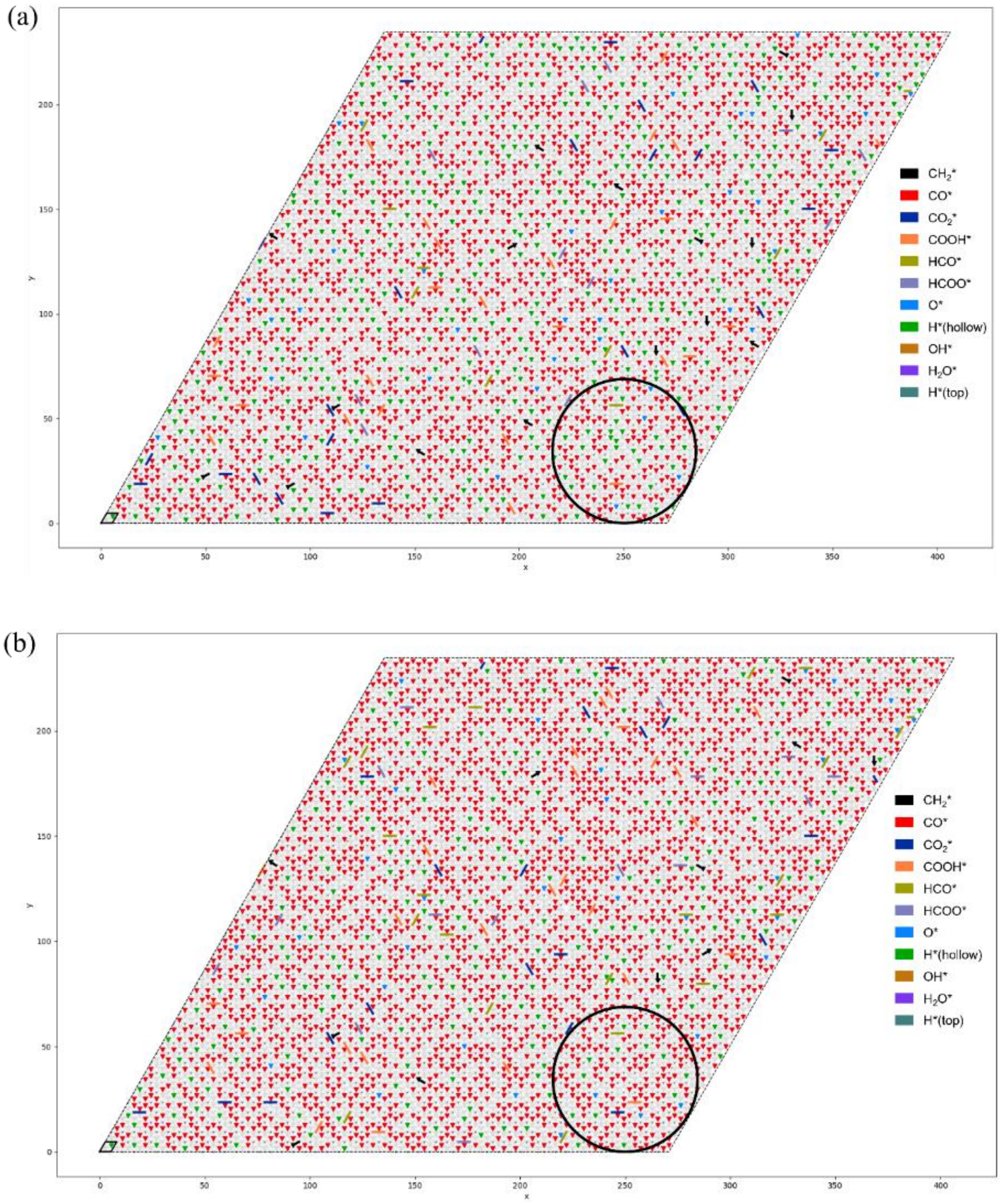

Figure S11. The adlayer configurations for the 50 × 50 lattice at the time of (a) 2055 s and (b) 2450 s at a temperature of 573.15 K. The partial pressures for H_2 and CO_2 are 0.8 bar and 0.2 bar, respectively. The black circles represent a random selection of the same area and position, aimed at facilitating a clearer comparison of the coverage of surface species.

S12. Impact of pressure on selectivity

Table S12. The selectivity of the carbon-based gas products, and the coverage of the surface species over the Rh(111) surface under the different reaction conditions.

	**T=473.15 K, P_{H_2}=0.8 bar, P_{CO_2}=0.2 bar**	**T=473.15 K, P_{H_2}=8 bar, P_{CO_2}=2 bar**	**T=573.15 K, P_{H_2}=0.8 bar, P_{CO_2}=0.2 bar**	**T=573.15 K, P_{H_2}=8 bar, P_{CO_2}=2 bar**
Selectivity	CH_4: 0.759<br>HCOOH: 0.241	CH_4: 0.912<br>HCOOH: 0.088	CH_4: 0.147<br>CO: 0.812	CH_4: 0.156<br>CO: 0.758
θ_{CO}	0.319	0.154	0.554	0.515
θ_{CO_2}	0.009	0.004	0.007	0.005
θ_{COOH}	0.007	0.002	0.004	0.006
θ_{HCOO}	0.005	0.003	0.002	0.005
θ_{O}	0.004	0.002	0.006	0.006
θ_{H}(hollow)	0.210	0.351	0.029	0.036
θ_{OH}	0.002	0.001	0.005	0.008
θ_{H_2O}	0.026	0.009	0.045	0.053
θ_{H}(top)	0.417	0.467	0.330	0.349

S13. Apparent activation energy

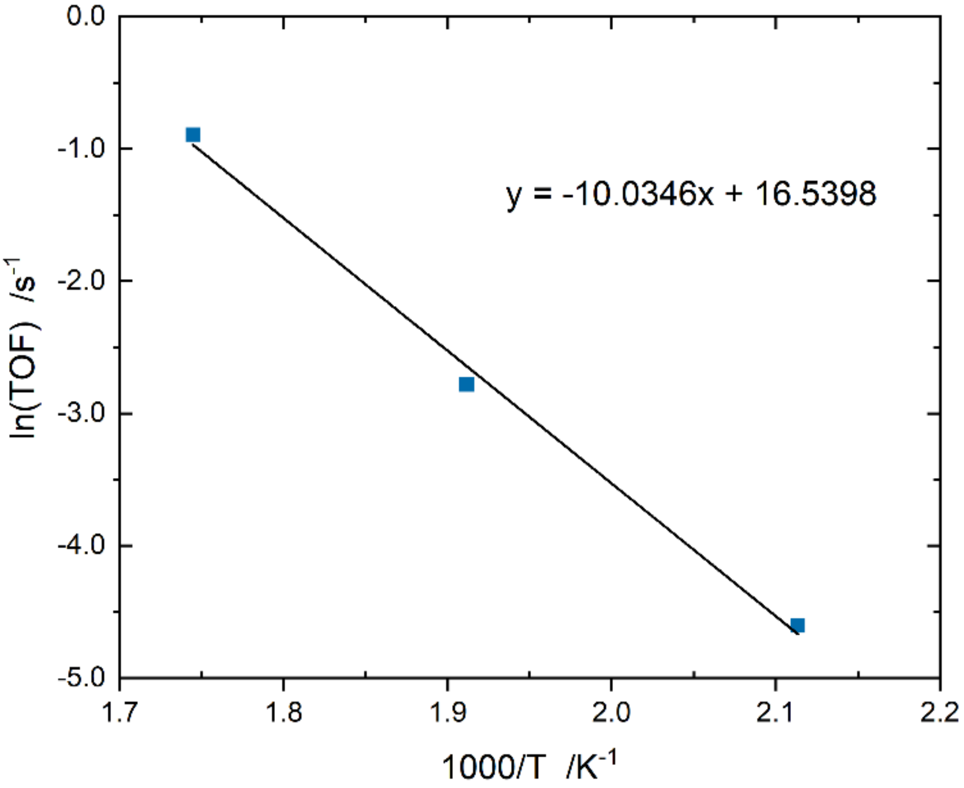

Figure S13. The apparent activation energy calculated via Arrhenius equation at a pressure of 1 bar, along with the 4:1 H_2/CO_2 mixture.

S14. Configurations used in cluster expansion model

Teal, grey, red and white spheres represent Rh, C, O and H atoms, respectively.

One-body terms:

C, CH, CH_2, CH_3, CH_4 and CO adsorption configurations

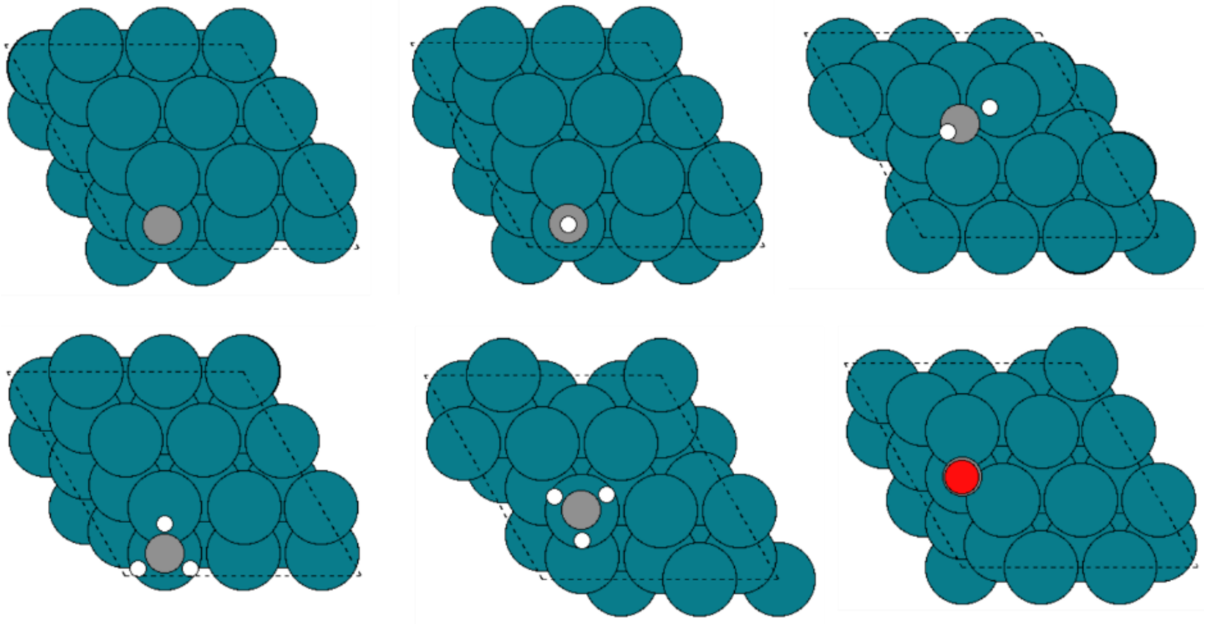

CO_2, COH, COOH, CH_2OH, CH_3OH, HCO, HCOH adsorption configurations

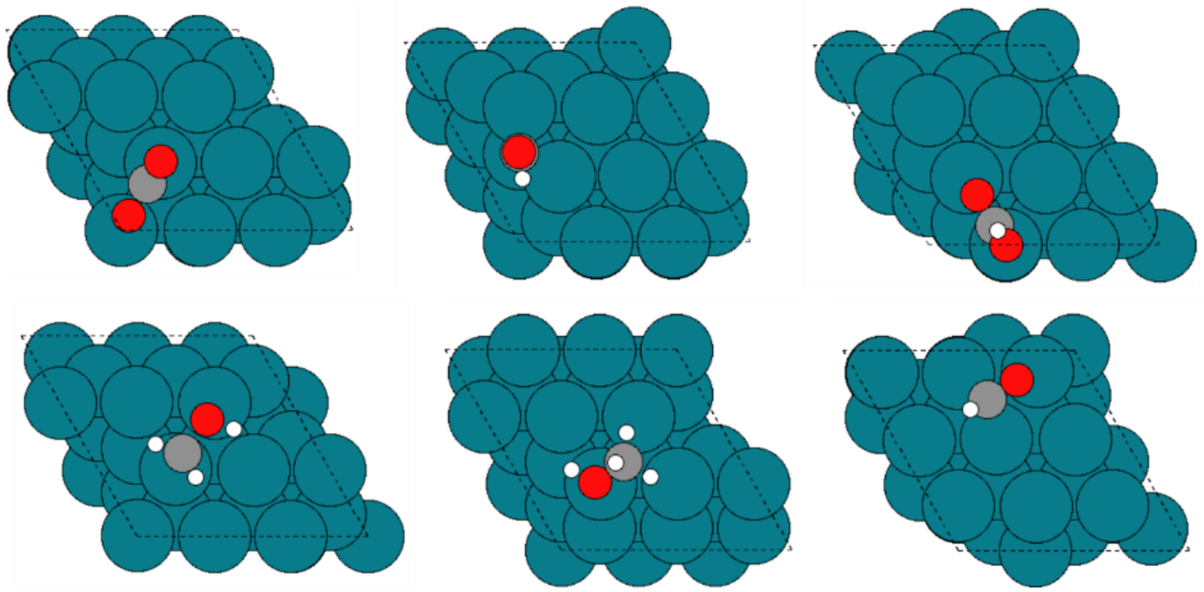

HCOO, HCOOH, H_2CO, H_2COO, H_2COOH, H_3CO adsorption configurations

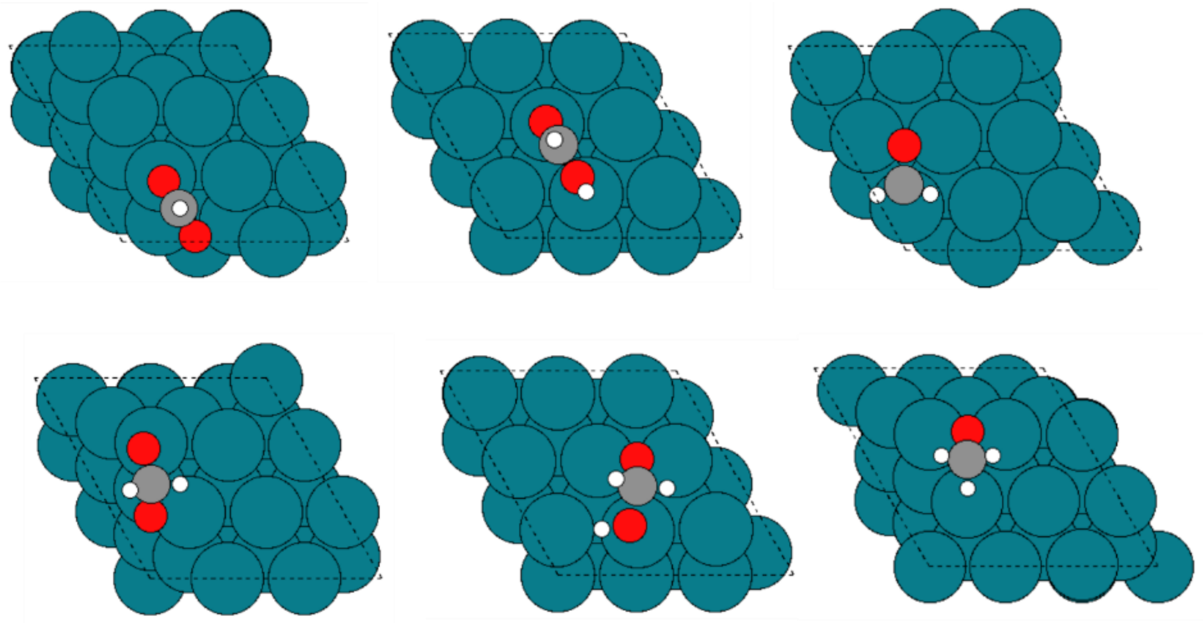

O, H, OH, H_2O, COHOH, 2H(top) adsorption configurations

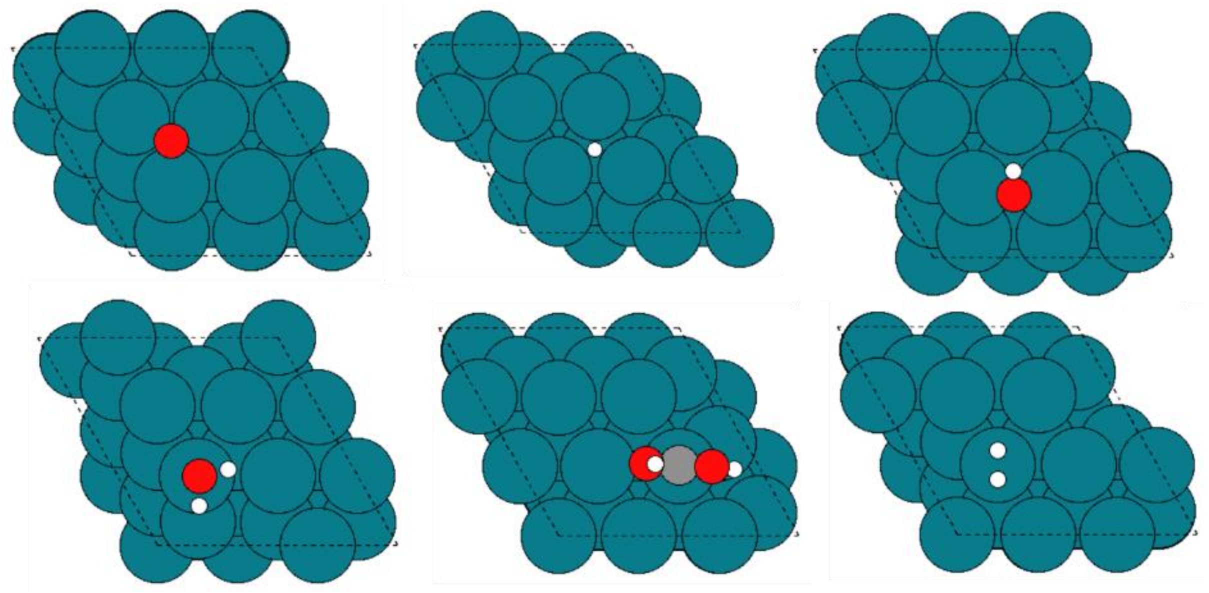

Two-body terms

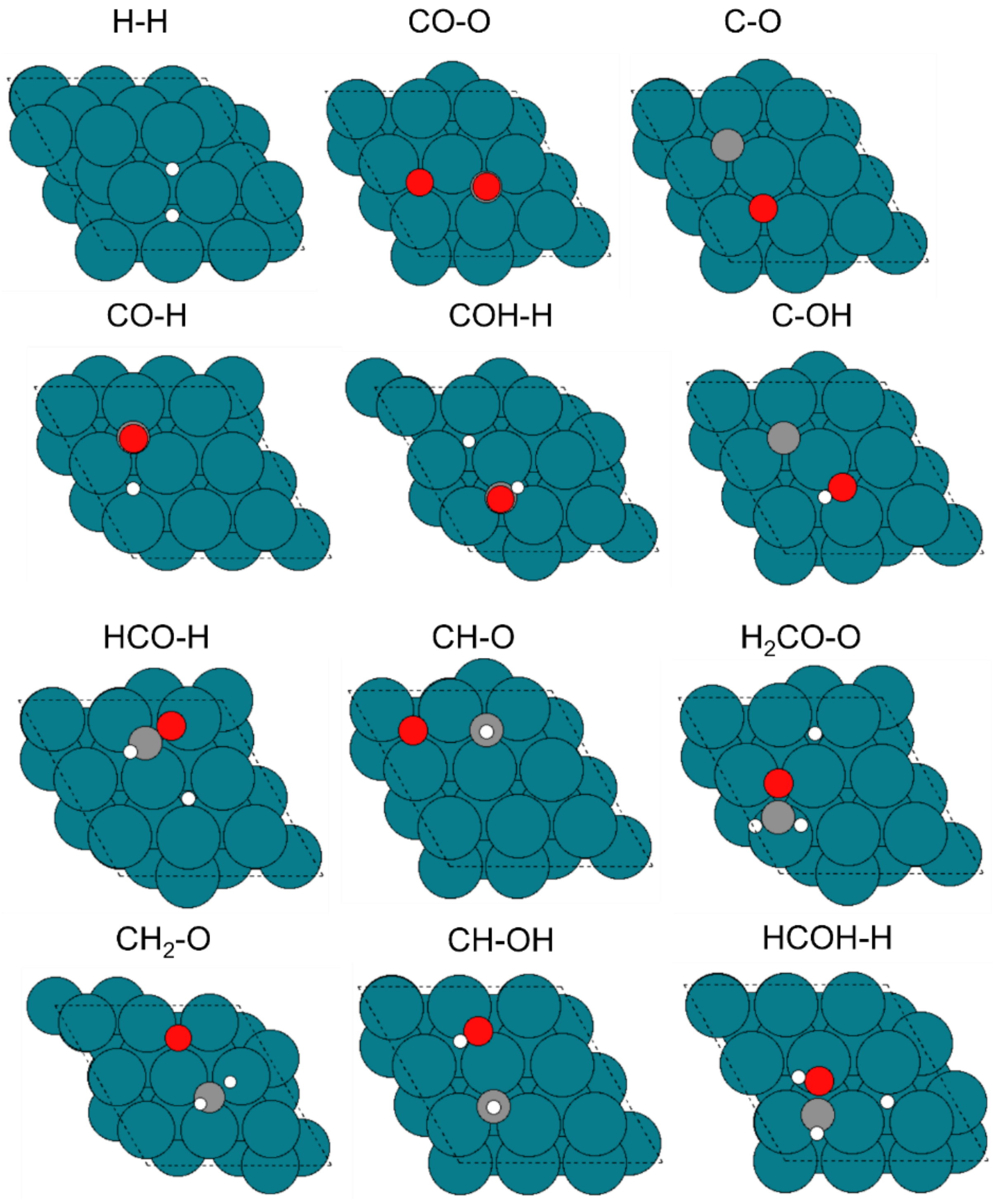

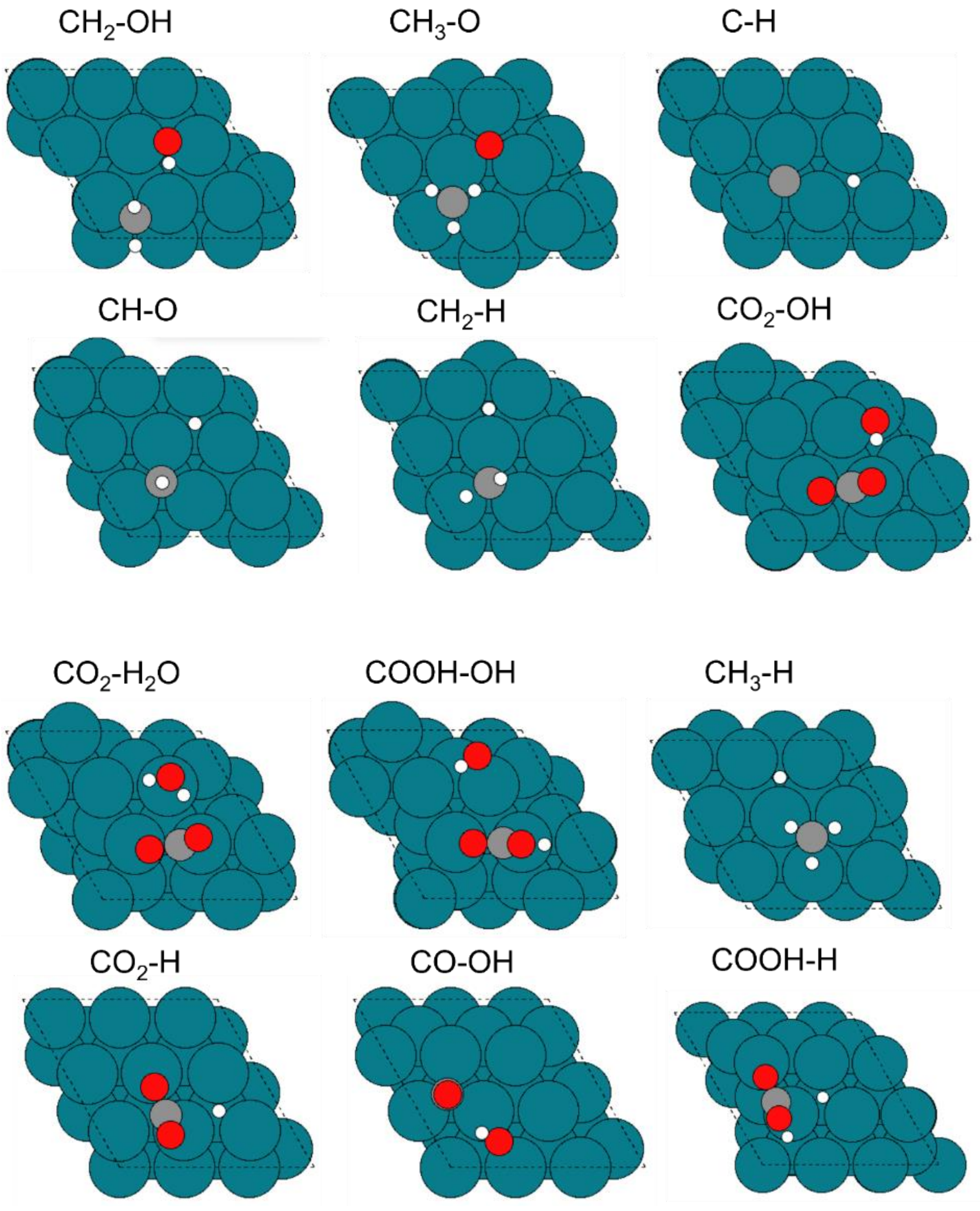
CH2-OH
CH3-O
C-H
CH-O
CH2-H
CO2-OH
CO2-H2O
COOH-OH
CH3-H
CO2-H
CO-OH
COOH-H

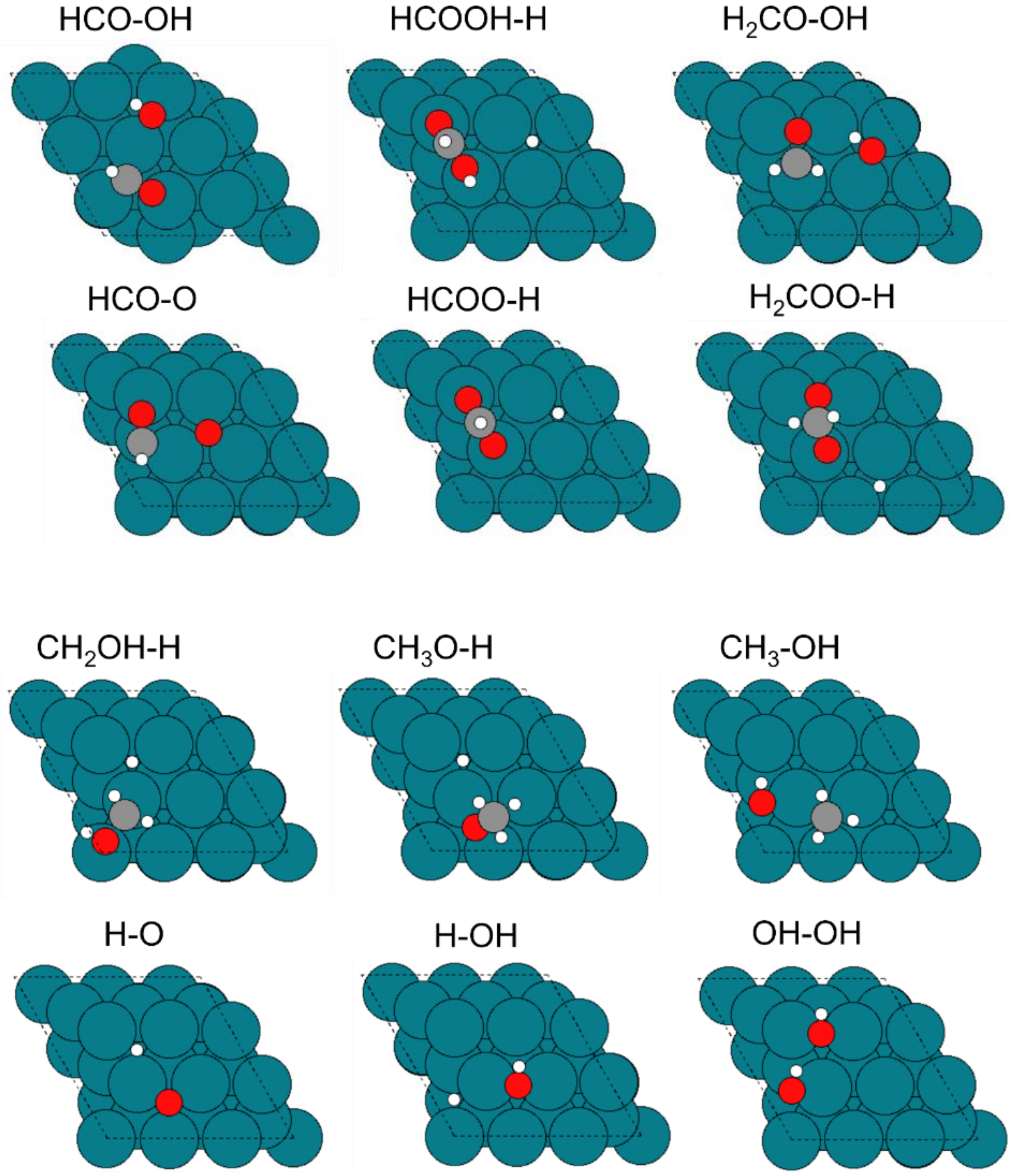
HCO-OH
HCOOH-H
H_2CO-OH
HCO-O
HCOO-H
H_2COO-H
CH_2OH-H
CH_3O-H
CH_3-OH
H-O
H-OH
OH-OH

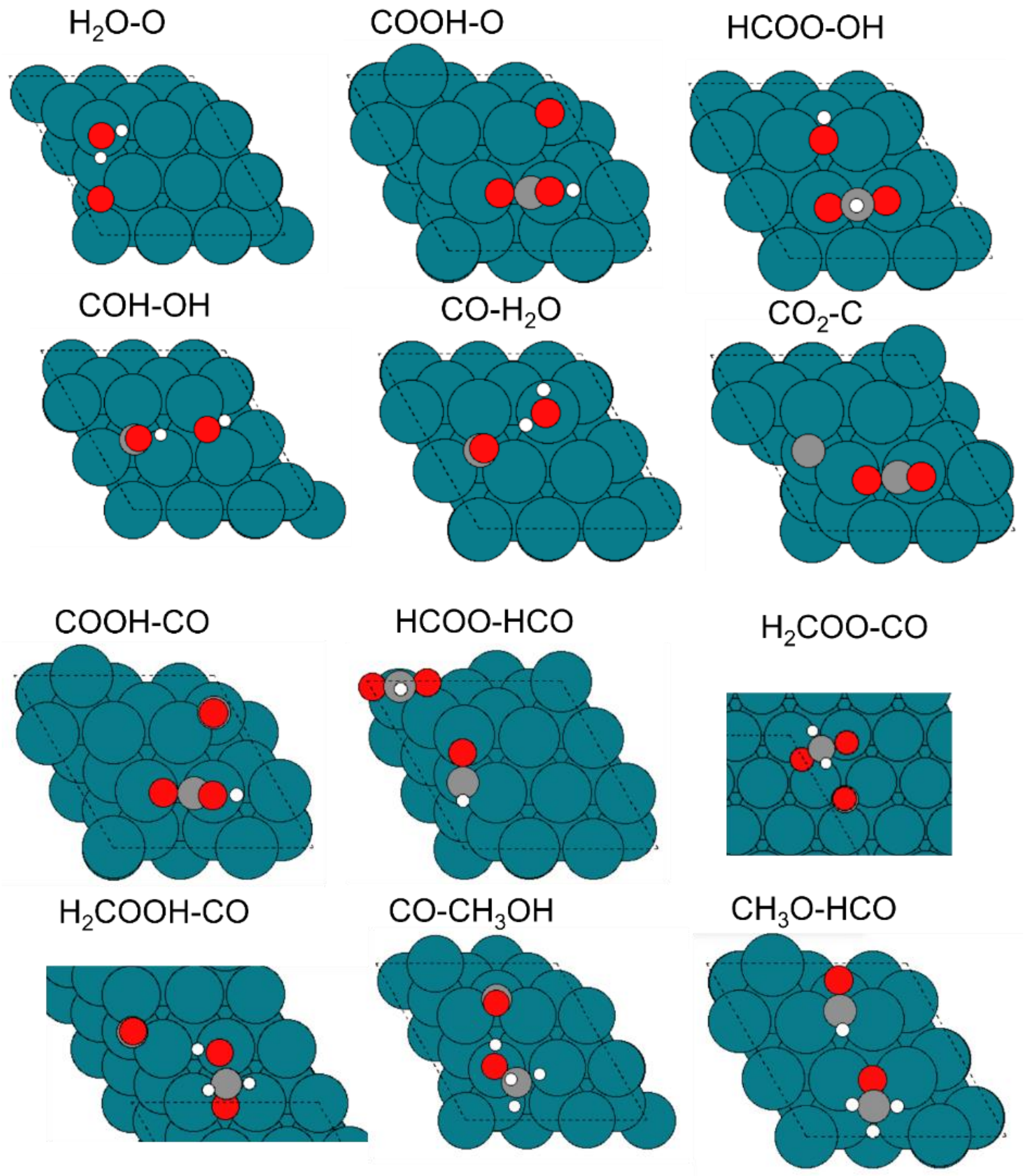
H_2O-O
COOH-O
HCOO-OH
COH-OH
CO-H_2O
CO_2-C
COOH-CO
HCOO-HCO
H_2COO-CO
H_2COOH-CO
CO-CH_3OH
CH_3O-HCO

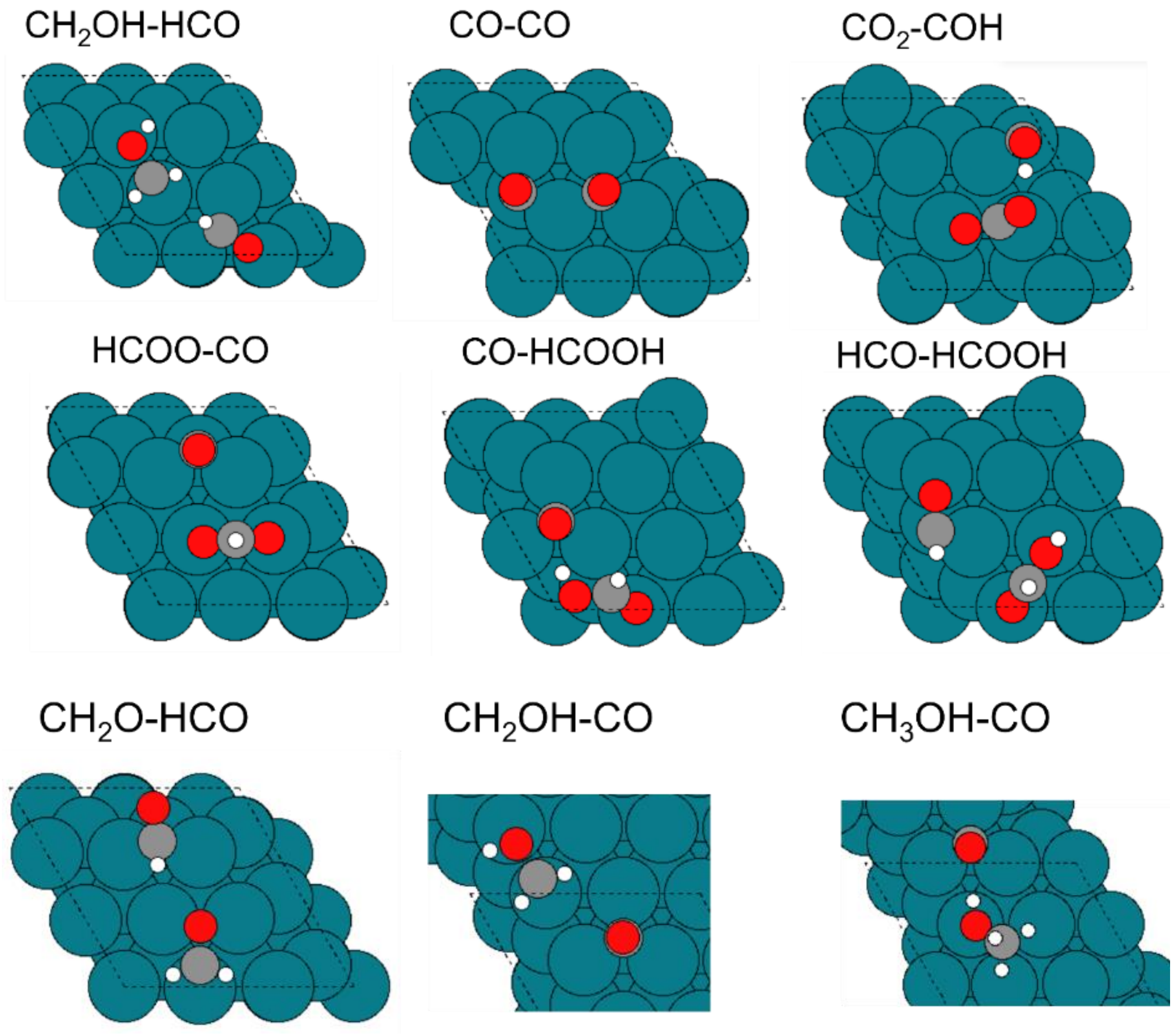
CH_2OH-HCO
CO-CO
CO_2-COH
HCOO-CO
CO-HCOOH
HCO-HCOOH
CH_2O-HCO
CH_2OH-CO
CH_3OH-CO

References

1. Polatoglou, H.; Methfessel, M.; Scheffler, M., Vacancy-formation energies at the (111) surface and in bulk Al, Cu, Ag, and Rh. *Physical Review B* **1993,** *48* (3), 1877.

2. Mittendorfer, F.; Seriani, N.; Dubay, O.; Kresse, G., Morphology of mesoscopic Rh and Pd nanoparticles under oxidizing conditions. *Physical Review B* **2007,** *76* (23), 233413.

3. Skriver, H. L.; Rosengaard, N., Surface energy and work function of elemental metals. *Physical Review B* **1992,** *46* (11), 7157.

4. Methfessel, M.; Hennig, D.; Scheffler, M., Trends of the surface relaxations, surface energies, and work functions of the 4d transition metals. *Physical Review B* **1992,** *46* (8), 4816.

5. Jian-Min, Z.; Fei, M.; Ke-Wei, X., Calculation of the surface energy of FCC metals with modified embedded-atom method. *Chinese Physics* **2004,** *13* (7), 1082.

6. Baskes, M. I., Modified embedded-atom potentials for cubic materials and impurities. *Physical review B* **1992,** *46* (5), 2727.

7. De Boer, F.; Boom, R.; Mattens, W.; Miedema, A.; Niessen, A., Cohesion in Metals North-Holland. Amsterdam: 1988.

8. Tyson, W.; Miller, W., Surface free energies of solid metals: Estimation from liquid surface tension measurements. *Surface Science* **1977,** *62* (1), 267-276.

9. Nielsen, J.; d'Avezac, M.; Hetherington, J.; Stamatakis, M., Parallel kinetic Monte Carlo simulation framework incorporating accurate models of adsorbate lateral interactions. *The Journal of chemical physics* **2013,** *139* (22), 224706.

10. Grabow, L. C.; Gokhale, A. A.; Evans, S. T.; Dumesic, J. A.; Mavrikakis, M., Mechanism of the water gas shift reaction on Pt: First principles, experiments, and microkinetic modeling. *The Journal of Physical Chemistry C* **2008,** *112* (12), 4608-4617.

11. Deimel, M.; Prats, H.; Seibt, M.; Reuter, K.; Andersen, M., Selectivity Trends and Role of Adsorbate–Adsorbate Interactions in CO Hydrogenation on Rhodium Catalysts. *ACS Catalysis* **2022,** *12* (13), 7907-7917.

12. Maintz, S.; Deringer, V. L.; Tchougréeff, A. L.; Dronskowski, R., Analytic projection from plane-wave and PAW wavefunctions and application to chemical-bonding analysis in solids. *Journal of computational chemistry* **2013,** *34* (29), 2557-2567.

13. Deringer, V. L.; Tchougréeff, A. L.; Dronskowski, R., Crystal orbital Hamilton population (COHP) analysis as projected from plane-wave basis sets. *The journal of physical chemistry A* **2011,** *115* (21), 5461-5466.

14. Dronskowski, R.; Blöchl, P. E., Crystal orbital Hamilton populations (COHP): energy-resolved visualization of chemical bonding in solids based on density-functional calculations. *The Journal of Physical Chemistry* **1993,** *97* (33), 8617-8624.

15. Abild-Pedersen, F.; Andersson, M. P., CO adsorption energies on metals with correction for high coordination adsorption sites – A density functional study. *Surface Science* **2007,** *601* (7), 1747-1753.

16. Wellendorff, J.; Silbaugh, T. L.; Garcia-Pintos, D.; Nørskov, J. K.; Bligaard, T.; Studt, F.; Campbell, C. T., A benchmark database for adsorption bond energies to transition

metal surfaces and comparison to selected DFT functionals. *Surface Science* **2015,** *640*, 36-44.
